# Supplementary material for: Structural brain network organization and aggressive behavior in schizophrenia: a mega-analysis of 2260 patients and 3058 healthy controls from the ENIGMA-Schizophrenia Working Group
Source: medRxiv. 2026 Oct 26:2026.10.22.26363656. Preprint. [Version 1] doi: 10.64898/2026.10.22.26363656 (PMC13734055; doi:10.64898/2026.10.22.26363656)
Supplement: Supplementary file 1 [file media-1.pdf]

APPENDICES

Lamsma et al. (2026). Structural brain network organization and aggressive behavior in schizophrenia: a mega-analysis of 2260 patients and 3058 healthy controls from the ENIGMA-Schizophrenia Working Group.

Table of contents

Appendix 1: Study and methodological details

Funding information for participating sites 4

Table A1.1. Scanner, acquisition, and processing details by site 8

Table A1.2. Items used to assess aggressive behavior and other clinical variables 15

Table A1.3. Definitions of canonical brain networks 16

Appendix 2: Sample characteristics

Table A2.1. Descriptive characteristics of participants by site 18

Table A2.2. Distribution of aggression scores in individuals with schizophrenia 19

Appendix 3: Primary analyses

Table A3.1. Group differences in global network topology 21

Table A3.2. Group differences in degree 22

Table A3.3. Group differences in betweenness centrality 24

Table A3.4. Group differences in harmonic centrality 26

Table A3.5. Group differences in within-module degree 28

Table A3.6. Group differences in participation coefficient 30

Table A3.7. Group differences in canonical brain network density 32

Appendix 4: Exploratory subgroup analyses

Table A4.1. Group differences in global network topology by illness stage 34

Table A4.2. Group differences in salience network-to-rest-of-brain density by delusion severity 35

Appendix 5: Sensitivity analyses

Analyses using a mild aggression cut-off

Table A5.1. Group differences in global network topology 36

Table A5.2. Group differences in degree 37

Table A5.3. Group differences in betweenness centrality 39

Table A5.4. Group differences in harmonic centrality 41

Table A5.5. Group differences in within-module degree 43

Table A5.6. Group differences in participation coefficient 45

Table A5.7. Group differences in canonical brain network density 47

Analyses using PANSS P7 'Hostility' scores only

Table A5.8. Group differences in global network topology 48

Table A5.9. Group differences in degree 49

Table A5.10. Group differences in betweenness centrality 51

Table A5.11. Group differences in harmonic centrality 53

Table A5.12. Group differences in within-module degree 55

Table A5.13. Group differences in participation coefficient 57

Table A5.14. Group differences in canonical brain network density 59

Analyses using regional volumes residualized for CPZ-equivalent dose

Table A5.15. Group differences in global network topology 60

Table A5.16. Group differences in degree 61

Table A5.17. Group differences in betweenness centrality 63

Table A5.18. Group differences in harmonic centrality 65

Table A5.19. Group differences in within-module degree 67

Table A5.20. Group differences in participation coefficient 69

Table A5.21. Group differences in canonical brain network density 71

Appendix 6: References

Appendix 1
Study and methodological details

Funding information for participating sites

CIAM

The CIAM study was supported by the South African Medical Research Council and the National Research Foundation of South Africa. FS is supported by the South African Medical Research Council through its Division of Research Capacity Development under the Early Investigators Program.

CLiNG

This work was supported by the German Research Foundation (grant GR1950/5-1 to OG).

COBRE

The COBRE study was supported by the National Institutes of Health (grants R01EB006841 and P20GM103472) and the National Science Foundation (grant 1539067). JAT and VDC are supported by the National Institutes of Health (grants 5R01MH094524 and 5R01MH121246).

ESO

The ESO study was supported by the Ministry of Health of the Czech Republic (grants NU21-08-00432 and NU22-04-00143).

FIDMAG

The FIDMAG study was supported by a Miguel Servet Research Contract (grant MS14/00041), the Plan Nacional de I+D+i 2013–2016 (grant PI14/00292), a Juan de la Cierva-Formación contract (grant FJCI-2015-25278), the Instituto de Salud Carlos III-Subdirección General de Evaluación y Fomento de la Investigación, the European Regional Development Fund, and the Centro de Investigación Biomédica en Red de Salud Mental.

FOR2107

This work was supported by grants from the German Research Foundation (DFG) awarded to TK through FOR2107 (KI 588/14-1 and KI 588/14-2) and SFB/TRR 393 (Trajectories of Affective Disorders; grants 521379614 and KI 588/23-1), to UD (DA 1151/5-1, DA 1151/5-2, DA 1151/9-1, DA 1151/10-1, DA 1151/11-1, and DA 1151/21-1), and to IN (NE 2254/1-2, NE 2254/2-1, NE 2254/3-1, and NE 2254/4-1). Additional funding was provided by Germany's Excellence Strategy (EXC 3066/1 The Adaptive Mind; grant 533717223), the DYNAMIC Center, funded by the LOEWE program of the Hessian Ministry of Science and Arts (grant LOEWE1/16/519/03/09.001(0009)/98), and the Wellcome Trust-funded DIALOG consortium (Understanding Disorganisation: A Language-Focused Global Initiative in Psychosis; grant 314138/Z/24/Z). UD was also supported by the Interdisciplinary Centre for Clinical Research of the University of Münster (grant Dan3/016/26).

GAP

The GAP study was supported by the NIHR Biomedical Research Centre at South London and Maudsley NHS Foundation Trust and King's College London.

IGP

The IGP study was supported by the Australian National Health and Medical Research Council (grants APP630471 and APP1081603), the Schizophrenia Research Institute Grant-in-Aid program, and the Australian Research Council Centre of Excellence in Cognition and its Disorders (grant CE110001021). MJG was supported by an Australian National Health and Medical Research Council R.D. Wright Biomedical Career Development Fellowship (grant

APP1061875). Recruitment through the Australian Schizophrenia Research Bank was supported by an Australian National Health and Medical Research Council Enabling Grant (grant 386500), the Pratt Foundation, Ramsay Health Care, the Viertel Charitable Foundation, and the Schizophrenia Research Institute.

IMH

This work was supported by grants from the National Healthcare Group (SIG/05004 and SIG/05028) and the Singapore Bioimaging Consortium (RP C009/2006), awarded to KS.

JBNU

The JBNU study was supported by the Korea Health Technology R&D Project through the Korea Health Industry Development Institute (KHIDI), funded by the Ministry of Health & Welfare (grant HR18C0016), and the National Research Foundation of Korea (NRF), funded by the Korean government (grants RS-2024-00335201 and RS-2025-16071835).

KaSP

The KaSP study was supported by the Swedish Research Council (grants 2024-02812, 2023-02827, 2017-00875, and 523-2014-3467), the Swedish Brain Foundation (grant FO2023-0333), Stockholm County Council (grants 20160328, 20180487, 20190175, and 20190447), the Torsten Söderberg Foundation, the Swedish Medical Society, the Erling Persson Foundation, and doctoral funding from Karolinska Institutet.

MCIC

The MCIC study was supported by the National Institutes of Health (grants P41RR14075 and R01EB005846 to VDC), the Department of Energy (grant DE-FG02-99ER62764), the Mind Research Network, the Morphometry (grant 1U24RR021382A) and Function (grants U24RR021992-01, MO1RR025758-01, and 1RC1MH089257 to VDC) testbeds of the Biomedical Informatics Research Network, the German Research Foundation (research fellowship to SE), the National Alliance for Research on Schizophrenia & Depression (Young Investigator Award to SE), and the Else Kröner-Fresenius Foundation (grant 2019-A118 to DA).

MPRC

The MPRC study was supported by the National Institutes of Health (grants U01MH108148, 2R01EB015611, R01MH112180, R01DA027680, R01MH085646, P50MH103222, and T32MH067533), a State of Maryland contract (M00B6400091), and the National Science Foundation (grant 1620457).

NU

The NU study was supported by the National Institutes of Health (grants P50MH071616, R01EB020062, R01MH056584, R01MH084803, and U01MH097435 to LW, and grant T32NS047987 to DC), and the National Science Foundation (grants 1636893 and 1734853 to LW).

Olin

The Olin study was supported by the National Institutes of Health (grant R01MH077945).

PAFIP

The PAFIP study was supported by the Instituto de Salud Carlos III (grant FIS PI14/00639), MINECO (grant SAF2013-46292-R), and the PSYSCAN project (European Commission grant agreement no. 603196). RRG and PS were supported by the Plan de Generación de Conocimiento (grant PID2021-122853OA-I00) and the Plan de Consolidación (grant CNS2023-143647), both

from the Agencia Estatal de Investigación, and by ERANET Neuron JTC 2023 (grant ERP-2023-23684211).

RomeSL
FP was supported by the Italian Ministry of Health (Ricerca Corrente 2023).

RSCZ
The RSCZ study was supported by the Russian Foundation for Basic Research (grant 20-013-00748).

SCORE
The SCORE study was supported by the Swiss National Science Foundation (grant 3232B0_119382). The FePsy Study Group from the University of Basel contributed to participant recruitment and was supported by the Swiss National Science Foundation (grant 3200-057216/1 and extensions 2 and 3).

SNUH
This work was supported by the National Research Foundation of Korea and Korea Brain Research Institute (grants 2019R1C1C1002457, 2020M3E5D9079910, RS-2023-00266120, and 21-BR-03-01).

SWIFT
The SWIFT study was supported by the Brain & Behavior Research Foundation (grant 28445) and the Novartis Foundation (grant 20A058).

UCISZ
The UCISZ study was supported by the National Institute of Mental Health (grant R21MH097196 to TvE). Data were processed with the high-performance computing cluster at the University of California, Irvine, supported by Joseph Farran, Harry Mangalam, Adam Brenner, the National Center for Research Resources, and the National Center for Advancing Translational Sciences (grant UL1TR000153).

UMCU
This work was supported by the Geestkracht program of the Netherlands Organization for Health Research and Development (grant 10-000-1001), the University Medical Centre Utrecht, participating pharmaceutical companies (Lundbeck, AstraZeneca, Eli Lilly, and Janssen Cilag), and participating mental healthcare institutions (Altrecht, GGZ Centraal, and Delta).

UNINA
FI, AdB, and AB were supported by NextGenerationEU, the Italian Ministry of University and Research, the National Recovery and Resilience Plan, and MNESYS – A Multiscale Integrated Approach to the Study of the Nervous System in Health and Disease (project PE0000006; Decreto n. 1553 11.10.2022).

VOICES
The VOICES study was supported by the National Health and Medical Research (NHMRC) Council (grant GNT1060664). SLR was supported by an NHMRC Senior Research Fellowship (grant GNT1154651). TEVR was supported by an Al and Val Rosenstrauss Fellowship from the Rebecca L. Cooper Medical Research Foundation and an NHMRC Early Career Fellowship. MH and WW were supported by the National Imaging Facility (NIF), and SC and PS by Australian Government Research Training Program Scholarships. The authors also acknowledge the

facilities and scientific assistance of the NIF, a National Collaborative Research Infrastructure Strategy capability, at the Swinburne Neuroimaging Facility, Swinburne University of Technology.

Zürich
The Zürich study was supported by the Swiss National Science Foundation (SNSF). MK is supported by the SNSF (grant 32003B_219240).

Table A1.1. Scanner, acquisition, and processing details by site.

Site	Scanner model	Field strength	Sequence	Parameters	Plane	FreeSurfer version	Operating system
CIAM	Siemens MAGNETOM Allegra	3T	MPRAGE	TE: 1.53/3.21/4.89/6.57 ms<br>TR: 2.53 s<br>Flip angle: 7°<br>Voxel size: 1.3 × 1 × 1.3 mm<br>FOV: 256 × 256 mm<br>Number of slices: 128	Sagittal	5.3.0	Linux
CLiNG	Siemens MAGNETOM TrioTim	3T	MPRAGE	TE: 3.26 ms<br>TR: 2.25 s<br>TI: 0.9 s<br>Flip angle: 9°<br>Voxel size: 1 × 1 × 1 mm<br>Matrix size: 256 × 256<br>Number of slices: 192	Sagittal	5.3.0	Ubuntu
COBRE	Siemens MAGNETOM Trio	3T	MPRAGE	TE: 1.64/3.5/5.36/7.22/9.08 ms<br>TR: 2.53 s<br>TI: 1.2 s<br>Flip angle: 7°<br>Voxel size: 1 × 1 × 1 mm<br>Matrix size: 256 × 256	Sagittal	5.3.0	RHEL
ESO	Siemens MAGNETOM Trio	3T	MPRAGE	TE: 4.63 ms<br>TR: 2.3 s<br>TI: 0.9 s<br>Voxel size: 1 × 1 × 1 mm<br>Matrix size: 256 × 256	Sagittal	5.3.0	Linux

FIDMAG	GE Signa	1.5T	MPRAGE	TE: 4 ms<br>TR: 2 s<br>Flip angle: 15°<br>Voxel size: 0.5 × 0.5 × 1 mm<br>Matrix size: 512 × 512<br>Number of slices: 180	Axial	5.3.0	Ubuntu
FOR2107	Siemens MAGNETOM TrioTim	3T	MPRAGE	TE: 2.26 ms<br>TR: 1.9 s<br>TI: 0.9 s<br>Flip angle: 9°<br>Voxel size: 1 × 1 × 1 mm<br>Matrix size: 256 × 256<br>Number of slices: 176	Sagittal	5.3.0	RHEL
	Siemens MAGNETOM Prisma	3T	MPRAGE	TE: 2.28 ms<br>TR: 2.13 s<br>TI: 0.9 s<br>Flip angle: 8°<br>Voxel size: 1 × 1 × 1 mm<br>Matrix size: 256 × 256<br>Number of slices: 192	Sagittal	5.3.0	RHEL
GAP	GE Signa HDx	3T	BRAVO	TE: 2.85 ms<br>TR: 6.99 s<br>TI: 0.65 s<br>Flip angle: 8°<br>Voxel size: 1.02 × 1.02 × 1.2 mm<br>Matrix size: 256 × 256<br>Number of slices: 166	Sagittal	5.3.0	Linux

IGP	Philips Achieva TX	3T	TFE	TE: 4.1 ms<br>TR: 8.9 ms<br>Flip angle: 8°<br>Voxel size: 0.83 × 0.83 × 0.9 mm<br>Matrix size: 288 × 288<br>Number of slices: 200	Sagittal	5.3.0	Mac OS X
IMH	Philips Achieva	3T	TFE	TE: 3.3 ms<br>TR: 7.2 s<br>Flip angle: 8°<br>Voxel size: 0.9 × 0.9 × 0.9 mm<br>Matrix size: 256 × 204<br>Number of slices: 180	Axial	5.3.0	Mac OS X
JBNU	Siemens MAGNETOM Verio	3T	MPRAGE	TE: 2.45 ms<br>TR: 1.9 s<br>Flip angle: 9°<br>Voxel size: 1 × 1 × 1 mm<br>FOV: 250 × 250 mm	Sagittal	7.1.0	Linux
KaSP	GE	3T	BRAVO	TE: 3.06 ms<br>TR: 7.90 ms<br>TI: 0.45 s<br>Flip angle: 12°<br>Voxel size: 0.93 × 0.93 × 1.2 mm<br>Matrix size: 256 × 256<br>Number of slices: 146	Sagittal	5.3.0	Linux
MCIC	Siemens	1.5T	FLASH	TE: 4.76 ms<br>TR: 12 ms<br>Flip angle: 20°<br>Voxel size: 0.63 × 0.63 × 1.5 mm<br>Matrix size: 256 × 256<br>Number of slices: 128	Coronal	4.0.1	Linux

	GE	3T	BRAVO	TE: 3.79 ms<br>TR: 2.53 s<br>TI: 1.1 s<br>Flip angle: 7°<br>Voxel size: 0.63 × 0.63 × 1.5 mm<br>Matrix size: 256 × 256<br>Number of slices: 128	Coronal	4.0.1	Linux
MPRC	Siemens MAGNETOM Allegra	3T	MPRAGE	TE: 4.3 ms<br>TR: 2.5 s<br>TI: 1 s<br>Flip angle: 8°<br>Voxel size: 1 × 1 × 1 mm	Sagittal	5.3.0	Linux
	Siemens MAGNETOM Trio	3T	MPRAGE	TE: 2.9 ms<br>TR: 2.3 s<br>TI: 0.9 s<br>Flip angle: 9°<br>Voxel size: 1 × 1 × 1 mm	Sagittal	5.3.0	Linux
NU	Siemens MAGNETOM Vision	1.5T	turbo-FLASH	TE: 5.4 ms<br>TR: 20 ms<br>Flip angle: 30°<br>Voxel size: 1 × 1 × 1 mm<br>Matrix size: 256 × 256<br>Number of slices: 180	Axial	5.3.0	CentOS
			MPRAGE	TE: 4 ms<br>TR: 9.7 ms<br>Flip angle: 10°<br>Voxel size: 1 × 1 × 1.25 mm<br>Matrix size: 256 × 256<br>Number of slices: 128	Axial	5.3.0	CentOS

Olin	Siemens MAGNETOM Allegra	3T	MPRAGE	TE: 4.13 ms<br>TR: 2.2 s<br>TI: 0.77 s<br>Flip angle: 13°<br>Voxel size: 0.8 × 0.8 × 0.8 mm<br>Matrix size: 240 × 320<br>Number of slices: 208	Axial	5.1.0	CentOS
PAFIP	GE Signa	1.5T	SPGR	TE: 5 ms<br>TR: 24 ms<br>Flip angle: 45°<br>Voxel size: 1.02 × 1.02 × 1.5 mm<br>Matrix size: 256 × 192	Coronal	6.0.0	Ubuntu
	Philips Achieva	3T	TFE	TE: 3.7 ms<br>TR: 8.2 ms<br>Flip angle: 8°<br>Voxel size: 0.94 × 0.94 × 1 mm<br>Matrix size: 256 × 256<br>Number of slices: 160	Sagittal	6.0.0	Ubuntu
RomeSL	Siemens MAGNETOM Allegra	3T	MDEFT	TE: 2.4 ms<br>TR: 7.92 ms<br>TI: 0.91 s<br>Flip angle: 15°<br>Voxel size: 1 × 1 × 1 mm<br>Matrix size: 224 × 256	Sagittal	6.0dev	Linux
RSCZ	Philips Achieva	3T	TFE	TE: 3.7 ms<br>TR: 8.2 s<br>TI: 1.02 s<br>Flip angle: 8°<br>Voxel size: 0.83 × 0.83 × 1 mm<br>FOV: 240 × 240 mm	Sagittal	5.3.0	CentOS

SCORE	Philips Achieva	3T	MPRAGE	TE: 3.4 ms<br>TR: 2 s<br>TI: 1 s<br>Flip angle: 8°<br>Voxel size: 1 × 1 × 1 mm<br>Matrix size: 256 × 256	Sagittal	7.4.1	macOS
SNUH	Siemens MAGNETOM Trio	3T	MPRAGE	TE: 1.89 ms<br>TR: 1.67 s<br>Flip angle: 9°<br>Voxel size: 1 × 1 × 1 mm<br>Matrix size: 250 × 250<br>Number of slices: 208	Sagittal	5.3.0	Mac OS X
SWIFT	Siemens MAGNETOM Verio	3T	MPRAGE	TE: 2.98 ms<br>TR: 2.3 s<br>TI: 0.9 s<br>Voxel size: 1 × 1 × 1 mm<br>Matrix size: 256 × 256<br>Number of slices: 160	Sagittal	7.1.1	Linux
UCISZ	Philips Achieva	3T	TFE	TE: 4.56 ms<br>TR: 11 ms<br>Flip angle: 18°<br>Voxel size: 0.75 × 0.75 × 0.75 mm<br>Matrix size: 320 × 274<br>Number of slices: 200	Sagittal	6.0dev	CentOS
UMCU	Philips Intera/Achieva	1.5T	FFE	TE: 4.6 ms<br>TR: 30 ms<br>Flip angle: 30°<br>Voxel size: 1 × 1 × 1.2 mm<br>Matrix size: 256 × 256<br>Number of slices: 160–180	Coronal	5.1.0	CentOS

UNINA	Siemens MAGNETOM TrioTim	3T	MPRAGE	TE: 3.4 ms<br>TR: 1.9 s<br>TI: 0.9 s<br>Flip angle: 9°<br>Voxel size: 1 × 1 × 1 mm<br>Number of slices: 160	Axial	6.0.1	Ubuntu
VOICES	Siemens MAGNETOM TrioTim	3T	MPRAGE	TE: 2.52 ms<br>TR: 1.9 s<br>Voxel size: 1 × 1 × 1 mm<br>Matrix size: 256 × 256<br>Number of slices: 176	Sagittal	6.0.0	macOS
Zürich	Philips	3T	TFE	TE: 3.8 ms<br>TR: 8.4 ms<br>Flip angle: 8°<br>Voxel size: 1 × 1 × 1 mm<br>Matrix size: 240 × 240<br>Number of slices: 160	Sagittal	6.0.0	Linux

Abbreviations: MPRAGE, Magnetization-Prepared Rapid Gradient Echo; TE, echo time; TR, repetition time; FOV, field of view; TI, inversion time; RHEL, Red Hat Enterprise Linux; GE, General Electric; BRAVO, Brain Volume Imaging; FLASH, Fast Low-Angle Shot; MDEFT, Modified Driven Equilibrium Fourier Transform; SPGR, spoiled gradient recalled echo; TFE, Turbo Field Echo; FFE, Fast Field Echo.

Table A1.2. Items used to assess aggressive behavior and other clinical variables.

Variable	Item
Aggressive behavior	BPRS item 10 'Hostility'<br>PANSS item P7 'Hostility'<br>SAPS item B3 'Aggressive and agitated behavior'
Delusions	PANSS item P1 'Delusions'<br>SAPS item D13 'Global rating of severity of delusions'
Hallucinations	BPRS item 12 'Hallucinatory behavior'<br>PANSS item P3 'Hallucinatory behavior'<br>SAPS item H7 'Global rating of severity of hallucinations'
Disorganized thinking	BPRS item 4 'Conceptual disorganization'<br>PANSS item P2 'Conceptual disorganization'<br>SAPS item P9 'Global rating of positive formal thought disorder'
Negative symptoms	Mean score on the negative symptoms subscale of the BPRS, PANSS, or SAPS
Poor impulse control	PANSS item P14 'Poor impulse control'
Lack of illness insight	PANSS item G12 'Lack of judgment and insight'

Abbreviations: BPRS, Brief Psychiatric Rating Scale; PANSS, Positive and Negative Syndrome Scale; SAPS, Scale for the Assessment of Positive Symptoms.

Table A1.3. Definitions of large-scale brain networks used in the present study.

Network	Constituent regions
Salience network (8)	INS, CACC
Central executive network (8)	RMFG, CMFG, IPL, SPL
Default mode network (93)	PCC, ISTH, PREC, MOFC, IPL, BANKS, MTG, TPOL, PHG, ENTH, HIPPO, NACC THAL
Language network (94)	POP, PTRI, STG, MTG, IPL
Reward network (95,96)	MOFC, LOFC, RACC, AMYG, NACC CAUD,PUT, THAL
Affective control network (10–12)	MOFC, LOFC, POP, PTRI, PORB, RMFG CMFG, SFG, RACC, CACC, INS, IPL, AMYG, NACC, THAL

Networks were selected based on their relevance to schizophrenia and aggression, as outlined in the Introduction, and operationalized using regions from the Desikan–Killiany atlas (44). Abbreviations: INS, insular cortex; CACC, caudal anterior cingulate cortex; RMFG, rostral middle frontal gyrus; CMFG, caudal middle frontal gyrus; IPL, inferior parietal lobule; SPL, superior parietal lobule; PCC, posterior cingulate cortex; ISTH, isthmus of the cingulate cortex; PREC, precuneus; MOFC, medial orbitofrontal cortex; IPL, inferior parietal lobule; BANKS, banks of the superior temporal sulcus; MTG, middle temporal gyrus; TPOL, temporal pole; PHG, parahippocampal gyrus; ENTH, entorhinal cortex; HIPPO, hippocampus; NACC, nucleus accumbens; THAL, thalamus; POP, pars opercularis; PTRI, pars triangularis; STG, superior temporal gyrus; MTG, middle temporal gyrus; IPL, inferior parietal lobule; MOFC, medial orbitofrontal cortex; LOFC, lateral orbitofrontal cortex; RACC, rostral anterior cingulate cortex; AMYG, amygdala; NACC, nucleus accumbens; CAUD, caudate nucleus; PUT, putamen; THAL, thalamus; MOFC, medial orbitofrontal cortex; LOFC, lateral orbitofrontal cortex; POP, pars opercularis; PTRI, pars triangularis; PORB, pars orbitalis; RMFG, rostral middle frontal gyrus; CMFG, caudal middle frontal gyrus; SFG, superior frontal gyrus; RACC, rostral anterior cingulate cortex; CACC, caudal anterior cingulate cortex; INS, insular cortex; IPL, inferior parietal lobule; AMYG, amygdala; NACC, nucleus accumbens; THAL, thalamus.

Appendix 2
Sample characteristics

Table A2.1. Descriptive characteristics of participants by site.

		SZ (*N* = 2260)			HC (*N* = 3058)		
Site	Country	*n*	Male, *n* (%)	Age, M (SD)	*n*	Male, *n* (%)	Age, M (SD)
CIAM	South Africa	18	11 (61)	31 (7)	31	17 (55)	27 (5)
CLiNG	Germany	48	36 (75)	33 (9)	322	132 (41)	25 (5)
COBRE	USA	62	51 (82)	37 (13)	63	44 (70)	37 (12)
ESO	Czechia	26	12 (46)	30 (7)	24	11 (46)	29 (7)
FIDMAG	Spain	124	95 (77)	38 (11)	123	54 (44)	38 (10)
FOR2107	Germany	93	45 (48)	38 (12)	733	250 (34)	34 (13)
GAP	UK	53	10 (19)	27 (6)	94	57 (61)	26 (7)
IGP	Australia	30	20 (67)	43 (9)	39	24 (62)	36 (11)
IMH	Singapore	160	108 (68)	34 (9)	102	59 (58)	33 (10)
JBNU	South Korea	370	184 (50)	38 (14)	354	171 (48)	41 (14)
KaSP	Sweden	81	50 (62)	29 (7)	56	28 (50)	27 (5)
MCIC	USA	107	81 (76)	34 (11)	92	63 (68)	33 (12)
MPRC	USA	104	79 (76)	34 (12)	175	76 (43)	37 (14)
NU	USA	105	72 (69)	34 (13)	77	43 (56)	31 (14)
Olin	USA	45	34 (76)	29 (10)	54	20 (37)	35 (12)
PAFIP	Spain	214	123 (57)	30 (9)	194	120 (62)	29 (8)
RomeSL	Italy	130	84 (65)	39 (11)	101	63 (62)	37 (11)
RSCZ	Russia	36	36 (100)	22 (3)	37	37 (100)	22 (3)
SCORE	Germany	63	45 (71)	27 (7)	44	17 (39)	26 (4)
SNUH	South Korea	39	18 (46)	23 (6)	40	20 (50)	23 (4)
SWIFT	Switzerland	23	16 (70)	35 (11)	13	5 (38)	29 (4)
UCISZ	USA	27	22 (81)	43 (11)	30	23 (77)	41 (12)
UMCU	Netherlands	167	134 (80)	27 (6)	138	67 (49)	27 (9)
UNINA	Italy	45	31 (69)	38 (10)	54	29 (54)	43 (15)
VOICES	Australia	30	9 (30)	42 (10)	40	18 (45)	32 (12)
Zürich	Switzerland	60	45 (75)	31 (8)	28	18 (64)	33 (9)

Abbreviations: SZ, schizophrenia; HC, healthy controls; M, mean; SD, standard deviation.

Table A2.2. Distribution of aggression scores in individuals with schizophrenia.

Score	BPRS item 10	PANSS item P7	SAPS item B3	Total
0 ‘None’	88 (53)	1111 (71)	318 (61)	1517 (67)
1 ‘Minimal’	28 (17)	232 (15)	54 (10)	314 (14)
2 ‘Mild’	20 (12)	134 (9)	44 (8)	198 (9)
3 ‘Moderate’	4 (2)	72 (5)	19 (4)	95 (4)
4 ‘Marked’	14 (8)	18 (1)	39 (8)	71 (3)
5 ‘Severe’	13 (8)	7 (<1)	45 (9)	65 (3)

Data are presented as *n* (%). Abbreviations: BPRS, Brief Psychiatric Rating Scale; PANSS, Positive and Negative Syndrome Scale; SAPS, Scale for the Assessment of Positive Symptoms.

Appendix 3

Primary analyses

Table A3.1. Group differences in global network topology.

	M (95% CI)			ΔAUC, p_{BH}		
Metric	SZ+A (*N* = 231)	SZ-A (*N* = 2029)	HC (*N* = 3058)	SZ+A vs SZ-A	SZ+A vs HC	SZ-A vs HC
Transitivity	0.47 (0.40, 0.51)	0.56 (0.51, 0.58)	0.50 (0.46, 0.52)	−0.014, .134	−0.005, .938	**0.009, .015**
Modularity	0.39 (0.31, 0.42)	0.30 (0.27, 0.33)	0.39 (0.36, 0.42)	**0.013, .012**	−0.001, .938	**−0.015, < .001**
Efficiency	0.47 (0.44, 0.51)	0.39 (0.37, 0.44)	0.45 (0.44, 0.48)	0.012, .084	0.003, .938	**−0.009, .001**
Small-worldness	1.57 (1.32, 1.81)	1.14 (1.11, 1.29)	1.47 (1.33, 1.65)	**0.064, .012**	0.017, .938	**−0.047, .005**

Means were calculated across a range of density thresholds (K = 0.10–0.25, ΔK = 0.01) and are reported with 95% bootstrap confidence intervals (CIs; 5000 resamples). Group differences in area under the curve (ΔAUC) were assessed using permutation testing (10 000 permutations). Bold values indicate statistically significant group differences after Benjamini–Hochberg correction for multiple comparisons (p_{BH} < .05). Abbreviations: SZ+A, schizophrenia with aggression; SZ−A, schizophrenia without aggression; HC, healthy controls.

Table A3.2. Group differences in degree.

Node	M (95% CI)			ΔAUC, p_{BH}		
	SZ+A (*N* = 231)	SZ-A (*N* = 2029)	HC (*N* = 3058)	SZ+A vs SZ-A	SZ+A vs HC	SZ-A vs HC
FPOL	2.1 (0.0, 7.8)	0.0 (0.0, 0.4)	1.0 (0.0, 2.1)	0.324, .434	0.180, .889	−0.145, .361
POP	3.5 (1.4, 9.5)	3.7 (1.8, 7.2)	3.9 (2.4, 6.4)	−0.025, .985	−0.070, .929	−0.045, .898
PTRI	5.0 (2.2, 8.5)	4.6 (3.0, 6.2)	5.1 (3.3, 6.1)	0.050, .950	−0.024, .948	−0.074, .883
PORB	6.0 (2.8, 8.6)	4.4 (3.1, 5.8)	4.7 (3.4, 7.2)	0.245, .759	0.200, .889	−0.045, .883
MOFC	11.8 (7.0, 16.4)	11.6 (8.4, 14.5)	9.0 (6.5, 11.2)	0.051, .976	0.432, .889	0.381, .580
LOFC	11.9 (7.6, 15.5)	18.4 (14.5, 20.6)	18.2 (13.4, 20.4)	−1.017, .672	−0.981, .889	0.036, .924
CMFG	6.8 (3.4, 11.0)	7.5 (5.2, 9.4)	8.2 (6.8, 10.0)	−0.136, .913	−0.215, .889	−0.079, .883
RMFG	12.4 (7.2, 16.1)	18.1 (13.9, 20.7)	14.9 (10.5, 17.8)	−0.840, .672	−0.361, .889	0.479, .580
SFG	16.6 (12.1, 19.1)	20.4 (15.8, 23.2)	17.9 (13.6, 21.3)	−0.573, .672	−0.202, .889	0.371, .779
PARL	5.7 (2.1, 9.9)	7.4 (4.8, 9.6)	8.1 (5.4, 10.4)	−0.246, .856	−0.346, .889	−0.099, .883
PREG	7.8 (4.8, 13.4)	11.5 (7.6, 15.2)	9.3 (6.9, 12.4)	−0.565, .759	−0.230, .889	0.335, .709
CACC	3.6 (2.4, 8.0)	2.5 (2.0, 3.4)	3.1 (2.1, 4.4)	0.159, .759	0.070, .889	−0.090, .580
RACC	8.9 (5.1, 11.9)	11.5 (8.6, 13.3)	8.5 (6.4, 11.2)	−0.381, .759	0.085, .929	0.466, .381
PCC	8.8 (3.3, 13.1)	11.9 (8.2, 15.1)	10.8 (7.9, 14.8)	−0.469, .759	−0.298, .889	0.171, .883
ISTH	4.6 (0.8, 9.4)	3.2 (1.6, 6.0)	3.6 (2.6, 6.6)	0.204, .897	0.149, .889	−0.054, .883
INS	16.7 (9.5, 19.4)	11.8 (8.4, 15.2)	7.9 (6.2, 10.7)	0.738, .672	1.327, .328	0.589, .231
TPOL	2.2 (0.0, 4.9)	0.0 (0.0, 0.5)	1.0 (0.9, 1.9)	0.345, .434	0.195, .889	**−0.150, .039**
ENTH	2.4 (0.2, 4.1)	0.2 (0.0, 1.2)	2.0 (1.9, 2.6)	0.329, .434	0.065, .889	**−0.265, .003**
PHG	4.4 (1.8, 6.9)	1.8 (1.0, 3.1)	3.4 (2.9, 5.4)	0.384, .672	0.145, .889	**−0.239, .039**
TVTG	7.0 (4.8, 11.2)	5.9 (3.6, 8.9)	8.2 (5.6, 9.8)	0.165, .913	−0.179, .889	−0.345, .490
ITG	10.1 (5.5, 15.0)	6.2 (3.9, 8.8)	8.1 (5.4, 11.1)	0.575, .672	0.284, .889	−0.291, .580
MTG	8.2 (4.9, 12.9)	11.3 (8.2, 13.7)	6.9 (5.4, 9.1)	−0.461, .759	0.195, .889	0.655, .071
STG	15.8 (9.9, 19.9)	13.9 (11.1, 15.8)	9.8 (7.8, 12.4)	0.261, .881	0.901, .889	0.640, .093
BANKS	3.6 (2.0, 5.8)	3.2 (3.0, 4.6)	3.6 (3.0, 4.7)	0.070, .913	0.015, .948	−0.055, .850
FUSI	13.4 (6.9, 17.8)	12.2 (8.9, 15.6)	10.7 (6.8, 14.6)	0.206, .913	0.424, .889	0.218, .883

POSG	12.2 (6.8, 16.4)	9.0 (6.0, 12.7)	8.2 (5.9, 11.2)	0.502, .759	0.598, .889	0.096, .883
PREC	10.7 (5.2, 14.6)	17.6 (12.4, 20.6)	18.9 (15.2, 21.0)	−1.051, .672	−1.252, .889	−0.201, .850
SMG	8.2 (4.8, 12.0)	7.6 (4.9, 10.8)	8.6 (6.2, 10.6)	0.082, .950	−0.066, .929	−0.148, .850
IPL	4.7 (2.8, 9.2)	9.7 (6.9, 12.4)	9.2 (7.0, 11.7)	−0.738, .672	−0.679, .889	0.060, .888
SPL	5.0 (2.8, 9.8)	5.5 (3.2, 8.8)	6.8 (4.2, 9.8)	−0.085, .950	−0.262, .889	−0.177, .850
LING	3.6 (2.1, 6.4)	3.2 (3.0, 3.9)	4.4 (4.0, 5.8)	0.055, .913	−0.120, .889	**−0.175, .039**
CUN	3.8 (2.9, 7.2)	3.0 (3.0, 4.2)	5.1 (4.1, 6.0)	0.115, .763	−0.201, .889	**−0.315, .039**
PERI	2.9 (2.0, 4.3)	2.1 (2.0, 2.7)	3.4 (2.9, 3.9)	0.135, .672	−0.075, .889	**−0.210, .003**
LOC	3.8 (1.6, 9.4)	5.7 (4.2, 8.0)	6.1 (4.4, 8.8)	−0.293, .759	−0.350, .889	−0.057, .883
HIPPO	3.0 (2.1, 7.4)	3.1 (2.2, 4.9)	4.4 (3.4, 5.5)	−0.005, .985	−0.220, .889	−0.215, .319
AMYG	5.9 (2.5, 10.3)	2.9 (2.1, 5.3)	4.2 (3.2, 6.2)	0.434, .672	0.244, .889	−0.190, .580
CAUD	2.9 (1.5, 4.0)	2.8 (2.1, 3.6)	3.6 (2.8, 4.4)	0.035, .950	−0.100, .889	**−0.135, .039**
PUT	6.8 (3.6, 9.4)	4.4 (3.4, 5.4)	5.8 (4.9, 6.4)	0.365, .434	0.150, .889	−0.215, .231
NACC	6.2 (2.6, 11.2)	1.9 (1.1, 2.8)	4.4 (3.3, 5.2)	0.635, .246	0.265, .889	**−0.370, .003**
PALL	3.4 (2.2, 7.1)	2.6 (2.0, 3.0)	2.0 (1.6, 2.0)	0.120, .759	0.205, .889	**0.085, .004**
THAL	4.6 (2.1, 8.4)	2.6 (1.5, 4.9)	3.9 (2.4, 5.6)	0.305, .759	0.105, .889	−0.200, .580

Means were calculated across a range of density thresholds (K = 0.10–0.25, ΔK = 0.01) and are reported with 95% bootstrap confidence intervals (CIs; 5000 resamples). Group differences in area under the curve (ΔAUC) were assessed using permutation testing (10 000 permutations). Bold values indicate statistically significant group differences after Benjamini–Hochberg correction for multiple comparisons ($p_{BH} < .05$). Abbreviations: SZ+A, schizophrenia with aggression; SZ−A, schizophrenia without aggression; HC, healthy controls; FPOL, frontal pole; POP, pars opercularis; PTRI, pars triangularis; PORB, pars orbitalis; MOFC, medial orbitofrontal cortex; LOFC, lateral orbitofrontal cortex; CMFG, caudal middle frontal gyrus; RMFG, rostral middle frontal gyrus; SFG, superior frontal gyrus; PARL, paracentral lobule; PREG, precentral gyrus; CACC, caudal anterior cingulate cortex; RACC, rostral anterior cingulate cortex; PCC, posterior cingulate cortex; ISTH, isthmus of the cingulate cortex; INS, insular cortex; TPOL, temporal pole; ENTH, entorhinal cortex; PHG, parahippocampal gyrus; TVTG, transverse temporal gyrus; ITG, inferior temporal gyrus; MTG, middle temporal gyrus; STG, superior temporal gyrus; BANKS, banks of the superior temporal sulcus; FUSI, fusiform gyrus; POSG, postcentral gyrus; PREC, precuneus; SMG, supramarginal gyrus; IPL, inferior parietal lobule; SPL, superior parietal lobule; LING, lingual gyrus; CUN, cuneus; PERI, pericalcarine cortex; LOC, lateral occipital cortex; HIPPO, hippocampus; AMYG, amygdala; CAUD, caudate nucleus; PUT, putamen; NACC, nucleus accumbens; PALL, globus pallidus; THAL, thalamus.

Table A3.3. Group differences in betweenness centrality.

	M (95% CI)			ΔAUC, p_{BH}		
Node	SZ+A (*N* = 231)	SZ-A (*N* = 2029)	HC (*N* = 3058)	SZ+A vs SZ-A	SZ+A vs HC	SZ-A vs HC
FPOL	0.000 (0.000, 0.013)	0.000 (0.000, 0.000)	0.000 (0.000, 0.000)	0.00000, 1.000	0.00000, 1.000	0.00000, 1.000
POP	0.001 (0.000, 0.034)	0.001 (0.000, 0.009)	0.001 (0.000, 0.007)	−0.00002, 1.000	−0.00001, 1.000	0.00001, 1.000
PTRI	0.015 (0.001, 0.044)	0.008 (0.001, 0.029)	0.004 (0.001, 0.022)	0.00111, .942	0.00166, .848	0.00055, 1.000
PORB	0.005 (0.000, 0.033)	0.010 (0.000, 0.035)	0.002 (0.000, 0.027)	−0.00043, .942	0.00047, .990	0.00090, .805
MOFC	0.038 (0.009, 0.131)	0.006 (0.002, 0.028)	0.018 (0.002, 0.043)	0.00496, .723	0.00311, .848	−0.00185, .670
LOFC	0.040 (0.011, 0.137)	0.067 (0.029, 0.124)	0.117 (0.037, 0.192)	−0.00428, .942	−0.01150, .848	−0.00722, .694
CMFG	0.003 (0.000, 0.050)	0.001 (0.000, 0.005)	0.016 (0.002, 0.028)	0.00022, .942	−0.00186, .848	−0.00208, .116
RMFG	0.054 (0.012, 0.117)	0.080 (0.031, 0.151)	0.069 (0.022, 0.131)	−0.00384, .942	−0.00237, .990	0.00147, 1.000
SFG	0.086 (0.033, 0.156)	0.174 (0.034, 0.286)	0.081 (0.030, 0.210)	−0.01257, .784	0.00135, .990	0.01392, .670
PARL	0.002 (0.000, 0.024)	0.001 (0.000, 0.004)	0.004 (0.001, 0.012)	0.00012, .942	−0.00038, .990	−0.00049, .259
PREG	0.004 (0.001, 0.083)	0.009 (0.002, 0.061)	0.011 (0.004, 0.068)	−0.00079, .942	−0.00085, .990	−0.00007, 1.000
CACC	0.001 (0.000, 0.027)	0.000 (0.000, 0.000)	0.000 (0.000, 0.000)	0.00017, .777	0.00017, .962	0.00000, 1.000
RACC	0.050 (0.005, 0.090)	0.016 (0.008, 0.030)	0.011 (0.005, 0.040)	0.00491, .553	0.00571, .848	0.00079, .895
PCC	0.031 (0.003, 0.103)	0.019 (0.009, 0.046)	0.036 (0.012, 0.119)	0.00209, .942	−0.00066, .990	−0.00275, .670
ISTH	0.064 (0.000, 0.125)	0.007 (0.000, 0.026)	0.022 (0.007, 0.056)	0.00885, .553	0.00687, .848	−0.00199, .521
INS	0.139 (0.024, 0.231)	0.012 (0.004, 0.118)	0.005 (0.002, 0.052)	0.01953, .553	0.02067, .848	0.00115, .670
TPOL	0.004 (0.000, 0.043)	0.000 (0.000, 0.000)	0.000 (0.000, 0.013)	0.00062, .723	0.00062, .886	0.00000, 1.000
ENTH	0.003 (0.000, 0.035)	0.000 (0.000, 0.018)	0.049 (0.018, 0.050)	0.00054, .784	−0.00686, .848	**−0.00740, .008**
PHG	0.027 (0.002, 0.111)	0.081 (0.000, 0.184)	0.297 (0.131, 0.346)	−0.00883, 1.000	−0.04093, .848	−0.03210, .116
TVTG	0.002 (0.001, 0.032)	0.001 (0.000, 0.009)	0.008 (0.002, 0.026)	0.00010, .956	−0.00096, .990	−0.00106, .521
ITG	0.050 (0.013, 0.158)	0.000 (0.000, 0.011)	0.086 (0.006, 0.173)	0.00749, .642	−0.00515, .990	−0.01264, .122
MTG	0.031 (0.007, 0.107)	0.016 (0.007, 0.083)	0.012 (0.004, 0.050)	0.00227, .942	0.00283, .848	0.00057, 1.000
STG	0.078 (0.019, 0.228)	0.032 (0.019, 0.072)	0.037 (0.017, 0.088)	0.00681, .723	0.00609, .848	−0.00072, 1.000
BANKS	0.001 (0.000, 0.015)	0.000 (0.000, 0.001)	0.001 (0.000, 0.002)	0.00012, .942	0.00003, .990	−0.00008, .116
FUSI	0.233 (0.069, 0.304)	0.152 (0.056, 0.279)	0.322 (0.157, 0.382)	0.01303, .942	−0.01294, .990	−0.02597, .238

POSG	0.036 (0.008, 0.128)	0.005 (0.002, 0.018)	0.013 (0.003, 0.035)	0.00465, .642	0.00344, .848	−0.00121, .670
PREC	0.084 (0.008, 0.159)	0.083 (0.038, 0.195)	0.255 (0.128, 0.336)	0.00003, 1.000	−0.02495, .848	−0.02498, .122
SMG	0.009 (0.002, 0.048)	0.003 (0.001, 0.014)	0.011 (0.004, 0.027)	0.00091, .942	−0.00027, .990	−0.00118, .238
IPL	0.008 (0.001, 0.053)	0.013 (0.006, 0.030)	0.029 (0.013, 0.111)	−0.00067, .942	−0.00310, .886	−0.00243, .536
SPL	0.024 (0.000, 0.074)	0.001 (0.000, 0.021)	0.019 (0.003, 0.045)	0.00342, .723	0.00117, .990	−0.00225, .515
LING	0.031 (0.000, 0.083)	0.021 (0.007, 0.027)	0.010 (0.002, 0.056)	0.00163, .942	0.00365, .848	0.00202, .339
CUN	0.014 (0.001, 0.092)	0.018 (0.007, 0.034)	0.020 (0.005, 0.039)	−0.00058, .956	−0.00059, .990	−0.00001, 1.000
PERI	0.000 (0.000, 0.014)	0.000 (0.000, 0.000)	0.000 (0.000, 0.001)	0.00001, .742	−0.00002, .990	**−0.00003, .037**
LOC	0.039 (0.001, 0.130)	0.113 (0.067, 0.131)	0.051 (0.019, 0.100)	−0.01105, .777	−0.00214, .990	0.00891, .116
HIPPO	0.002 (0.000, 0.073)	0.139 (0.003, 0.186)	0.173 (0.054, 0.228)	−0.02082, .784	−0.02605, .848	−0.00523, .933
AMYG	0.025 (0.002, 0.129)	0.046 (0.007, 0.145)	0.104 (0.015, 0.176)	−0.00352, .942	−0.01099, .900	−0.00747, .694
CAUD	0.001 (0.000, 0.016)	0.001 (0.000, 0.010)	0.001 (0.001, 0.014)	0.00014, .942	0.00007, .990	−0.00007, 1.000
PUT	0.058 (0.010, 0.108)	0.043 (0.010, 0.097)	0.074 (0.042, 0.092)	0.00221, .942	−0.00250, .990	−0.00471, .410
NACC	0.067 (0.005, 0.147)	0.001 (0.000, 0.010)	0.013 (0.003, 0.061)	0.00932, .553	0.00753, .848	−0.00179, .670
PALL	0.002 (0.000, 0.045)	0.008 (0.000, 0.030)	0.000 (0.000, 0.000)	−0.00102, .942	0.00028, .848	**0.00129, .004**
THAL	0.032 (0.001, 0.097)	0.030 (0.000, 0.112)	0.019 (0.002, 0.115)	0.00028, 1.000	0.00202, .990	0.00174, 1.000

Means were calculated across a range of density thresholds (K = 0.10–0.25, ΔK = 0.01) and are reported with 95% bootstrap confidence intervals (CIs; 5000 resamples). Group differences in area under the curve (ΔAUC) were assessed using permutation testing (10 000 permutations). Bold values indicate statistically significant group differences after Benjamini–Hochberg correction for multiple comparisons (p_{BH} < .05). Abbreviations: SZ+A, schizophrenia with aggression; SZ−A, schizophrenia without aggression; HC, healthy controls; FPOL, frontal pole; POP, pars opercularis; PTRI, pars triangularis; PORB, pars orbitalis; MOFC, medial orbitofrontal cortex; LOFC, lateral orbitofrontal cortex; CMFG, caudal middle frontal gyrus; RMFG, rostral middle frontal gyrus; SFG, superior frontal gyrus; PARL, paracentral lobule; PREG, precentral gyrus; CACC, caudal anterior cingulate cortex; RACC, rostral anterior cingulate cortex; PCC, posterior cingulate cortex; ISTH, isthmus of the cingulate cortex; INS, insular cortex; TPOL, temporal pole; ENTH, entorhinal cortex; PHG, parahippocampal gyrus; TVTG, transverse temporal gyrus; ITG, inferior temporal gyrus; MTG, middle temporal gyrus; STG, superior temporal gyrus; BANKS, banks of the superior temporal sulcus; FUSI, fusiform gyrus; POSG, postcentral gyrus; PREC, precuneus; SMG, supramarginal gyrus; IPL, inferior parietal lobule; SPL, superior parietal lobule; LING, lingual gyrus; CUN, cuneus; PERI, pericalcarine cortex; LOC, lateral occipital cortex; HIPPO, hippocampus; AMYG, amygdala; CAUD, caudate nucleus; PUT, putamen; NACC, nucleus accumbens; PALL, globus pallidus; THAL, thalamus.

Table A3.4. Group differences in harmonic centrality.

	M (95% CI)			ΔAUC, p_{BH}		
Node	SZ+A (*N* = 231)	SZ-A (*N* = 2029)	HC (*N* = 3058)	SZ+A vs SZ-A	SZ+A vs HC	SZ-A vs HC
FPOL	0.384 (0.000, 0.519)	0.000 (0.000, 0.160)	0.204 (0.000, 0.377)	0.059, .465	0.029, .928	−0.030, .337
POP	0.408 (0.309, 0.542)	0.388 (0.305, 0.469)	0.412 (0.359, 0.472)	0.003, .904	−0.001, .976	−0.003, .764
PTRI	0.463 (0.346, 0.531)	0.410 (0.354, 0.458)	0.441 (0.393, 0.469)	0.008, .787	0.003, .928	−0.005, .484
PORB	0.482 (0.408, 0.539)	0.418 (0.390, 0.457)	0.445 (0.423, 0.496)	0.010, .588	0.006, .928	−0.004, .371
MOFC	0.590 (0.507, 0.661)	0.525 (0.486, 0.575)	0.520 (0.480, 0.562)	0.010, .588	0.011, .858	0.001, .940
LOFC	0.595 (0.520, 0.654)	0.620 (0.572, 0.666)	0.655 (0.590, 0.696)	−0.004, .828	−0.009, .909	−0.005, .484
CMFG	0.490 (0.432, 0.566)	0.463 (0.435, 0.501)	0.495 (0.479, 0.538)	0.004, .828	−0.001, .976	−0.005, .306
RMFG	0.586 (0.507, 0.650)	0.622 (0.568, 0.670)	0.602 (0.539, 0.654)	−0.005, .828	−0.002, .976	0.003, .764
SFG	0.638 (0.579, 0.692)	0.657 (0.587, 0.718)	0.648 (0.592, 0.711)	−0.003, .869	−0.001, .976	0.001, .925
PARL	0.470 (0.400, 0.549)	0.457 (0.423, 0.505)	0.508 (0.465, 0.547)	0.002, .869	−0.005, .928	−0.008, .253
PREG	0.498 (0.450, 0.609)	0.524 (0.472, 0.590)	0.527 (0.492, 0.580)	−0.004, .869	−0.004, .928	−0.000, .965
CACC	0.426 (0.389, 0.528)	0.363 (0.338, 0.406)	0.406 (0.370, 0.440)	0.009, .698	0.003, .928	−0.006, .217
RACC	0.552 (0.468, 0.600)	0.523 (0.484, 0.562)	0.508 (0.475, 0.565)	0.005, .828	0.007, .928	0.002, .745
PCC	0.553 (0.409, 0.619)	0.529 (0.480, 0.582)	0.553 (0.505, 0.622)	0.004, .828	0.000, .976	−0.004, .643
ISTH	0.379 (0.159, 0.552)	0.401 (0.359, 0.464)	0.448 (0.425, 0.498)	−0.003, .903	−0.010, .928	−0.007, .266
INS	0.659 (0.549, 0.708)	0.528 (0.485, 0.598)	0.505 (0.482, 0.560)	0.020, .465	0.023, .623	0.004, .609
TPOL	0.344 (0.000, 0.472)	0.000 (0.000, 0.114)	0.245 (0.226, 0.352)	0.053, .465	0.016, .928	**−0.037, .023**
ENTH	0.345 (0.040, 0.445)	0.076 (0.000, 0.259)	0.320 (0.300, 0.377)	0.042, .465	0.005, .928	**−0.037, .023**
PHG	0.440 (0.314, 0.505)	0.362 (0.290, 0.413)	0.434 (0.405, 0.481)	0.012, .694	0.001, .976	**−0.011, .040**
TVTG	0.495 (0.467, 0.576)	0.442 (0.403, 0.504)	0.517 (0.470, 0.547)	0.008, .787	−0.003, .928	−0.011, .115
ITG	0.560 (0.479, 0.645)	0.450 (0.407, 0.505)	0.529 (0.471, 0.589)	0.017, .465	0.004, .928	−0.012, .150
MTG	0.529 (0.458, 0.611)	0.524 (0.479, 0.576)	0.493 (0.457, 0.541)	0.001, .928	0.005, .928	0.005, .509
STG	0.644 (0.560, 0.713)	0.559 (0.528, 0.603)	0.538 (0.506, 0.585)	0.013, .465	0.016, .734	0.003, .574
BANKS	0.428 (0.361, 0.494)	0.371 (0.362, 0.424)	0.408 (0.390, 0.448)	0.009, .763	0.003, .928	−0.005, .253
FUSI	0.623 (0.506, 0.692)	0.554 (0.505, 0.619)	0.574 (0.516, 0.643)	0.011, .694	0.008, .928	−0.003, .745

POSG	0.576 (0.496, 0.652)	0.484 (0.445, 0.552)	0.520 (0.478, 0.567)	0.014, .516	0.009, .928	−0.006, .475
PREC	0.584 (0.467, 0.642)	0.616 (0.549, 0.675)	0.676 (0.632, 0.716)	−0.005, .828	−0.014, .827	−0.009, .220
SMG	0.511 (0.454, 0.588)	0.461 (0.422, 0.520)	0.520 (0.484, 0.557)	0.008, .792	−0.001, .976	−0.009, .160
IPL	0.462 (0.390, 0.556)	0.502 (0.464, 0.558)	0.538 (0.507, 0.585)	−0.006, .828	−0.011, .827	−0.006, .337
SPL	0.470 (0.396, 0.560)	0.436 (0.395, 0.499)	0.490 (0.447, 0.545)	0.005, .828	−0.003, .976	−0.008, .337
LING	0.313 (0.203, 0.484)	0.332 (0.309, 0.367)	0.401 (0.372, 0.467)	−0.003, .883	−0.013, .909	**−0.010, .046**
CUN	0.313 (0.220, 0.500)	0.327 (0.309, 0.398)	0.446 (0.391, 0.480)	−0.002, .904	−0.020, .623	**−0.018, .023**
PERI	0.290 (0.188, 0.423)	0.258 (0.242, 0.319)	0.362 (0.338, 0.392)	0.005, .828	−0.011, .827	**−0.016, .008**
LOC	0.349 (0.226, 0.560)	0.454 (0.413, 0.509)	0.478 (0.417, 0.541)	−0.016, .516	−0.020, .827	−0.004, .607
HIPPO	0.367 (0.310, 0.522)	0.276 (0.145, 0.439)	0.377 (0.335, 0.453)	0.014, .828	−0.001, .976	−0.015, .217
AMYG	0.455 (0.334, 0.573)	0.235 (0.150, 0.416)	0.366 (0.310, 0.468)	0.033, .588	0.014, .928	−0.019, .175
CAUD	0.357 (0.261, 0.415)	0.188 (0.129, 0.287)	0.278 (0.254, 0.347)	0.026, .465	0.012, .928	−0.014, .118
PUT	0.475 (0.352, 0.536)	0.236 (0.155, 0.348)	0.346 (0.309, 0.406)	0.036, .312	0.020, .827	−0.017, .118
NACC	0.491 (0.359, 0.582)	0.180 (0.116, 0.285)	0.323 (0.286, 0.400)	0.047, .160	0.025, .827	**−0.021, .033**
PALL	0.370 (0.295, 0.489)	0.198 (0.127, 0.296)	0.258 (0.230, 0.291)	0.026, .465	0.017, .827	−0.009, .217
THAL	0.437 (0.301, 0.540)	0.223 (0.137, 0.399)	0.328 (0.290, 0.439)	0.032, .588	0.017, .928	−0.016, .276

Means were calculated across a range of density thresholds ($K = 0.10–0.25$, $\Delta K = 0.01$) and are reported with 95% bootstrap confidence intervals (CIs; 5000 resamples). Group differences in area under the curve (ΔAUC) were assessed using permutation testing (10 000 permutations). Bold values indicate statistically significant group differences after Benjamini–Hochberg correction for multiple comparisons ($p_{BH} < .05$). Abbreviations: SZ+A, schizophrenia with aggression; SZ−A, schizophrenia without aggression; HC, healthy controls; FPOL, frontal pole; POP, pars opercularis; PTRI, pars triangularis; PORB, pars orbitalis; MOFC, medial orbitofrontal cortex; LOFC, lateral orbitofrontal cortex; CMFG, caudal middle frontal gyrus; RMFG, rostral middle frontal gyrus; SFG, superior frontal gyrus; PARL, paracentral lobule; PREG, precentral gyrus; CACC, caudal anterior cingulate cortex; RACC, rostral anterior cingulate cortex; PCC, posterior cingulate cortex; ISTH, isthmus of the cingulate cortex; INS, insular cortex; TPOL, temporal pole; ENTH, entorhinal cortex; PHG, parahippocampal gyrus; TVTG, transverse temporal gyrus; ITG, inferior temporal gyrus; MTG, middle temporal gyrus; STG, superior temporal gyrus; BANKS, banks of the superior temporal sulcus; FUSI, fusiform gyrus; POSG, postcentral gyrus; PREC, precuneus; SMG, supramarginal gyrus; IPL, inferior parietal lobule; SPL, superior parietal lobule; LING, lingual gyrus; CUN, cuneus; PERI, pericalcarine cortex; LOC, lateral occipital cortex; HIPPO, hippocampus; AMYG, amygdala; CAUD, caudate nucleus; PUT, putamen; NACC, nucleus accumbens; PALL, globus pallidus; THAL, thalamus.

Table A3.5. Group differences in within-module degree.

	M (95% CI)			ΔAUC, p_{BH}		
Node	SZ+A (*N* = 231)	SZ-A (*N* = 2029)	HC (*N* = 3058)	SZ+A vs SZ-A	SZ+A vs HC	SZ-A vs HC
FPOL	−1.52 (−1.85, 0.00)	0.00 (0.00, 0.00)	−1.76 (−1.87, 0.00)	−0.226, .827	0.037, .991	0.263, .198
POP	−1.22 (−1.61, 0.36)	−1.15 (−1.43, −0.23)	−1.07 (−1.46, −0.50)	−0.011, .991	−0.024, .991	−0.013, .917
PTRI	−0.77 (−1.43, 0.21)	−0.94 (−1.18, 0.72)	−0.78 (−1.24, −0.50)	0.023, .917	−0.001, .991	−0.024, .896
PORB	−0.51 (−1.34, 0.16)	−0.99 (−1.20, −0.36)	−0.87 (−1.20, −0.35)	0.070, .861	0.052, .991	−0.018, .896
MOFC	0.87 (−0.36, 1.90)	0.40 (−0.20, 1.01)	0.08 (−0.50, 0.62)	0.073, .908	0.119, .991	0.047, .896
LOFC	0.81 (−0.22, 1.62)	1.78 (0.97, 2.20)	2.10 (1.13, 2.68)	−0.152, .827	−0.199, .977	−0.047, .896
CMFG	−0.39 (−1.15, 0.68)	−0.38 (−0.79, 0.06)	−0.02 (−0.36, 0.37)	−0.006, .991	−0.054, .991	−0.048, .683
RMFG	1.07 (−0.26, 1.78)	1.57 (0.80, 2.10)	1.25 (0.38, 1.90)	−0.073, .908	−0.026, .991	0.047, .896
SFG	2.04 (1.07, 2.66)	1.99 (1.24, 2.50)	2.22 (1.31, 2.85)	0.012, .991	−0.023, .991	−0.035, .896
PARL	−0.64 (−1.44, 0.39)	−0.43 (−0.86, 0.06)	−0.11 (−0.67, 0.46)	−0.030, .917	−0.077, .991	−0.047, .896
PREG	−0.09 (−0.79, 1.18)	0.38 (−0.34, 1.11)	0.18 (−0.36, 0.85)	−0.074, .908	−0.043, .991	0.031, .896
CACC	−1.20 (−1.51, −0.02)	−1.34 (−1.53, −1.09)	−1.24 (−1.53, −0.93)	0.019, .917	0.004, .991	−0.016, .896
RACC	−0.09 (−0.92, 0.74)	0.41 (−0.14, 0.81)	−0.02 (−0.49, 0.62)	−0.073, .908	−0.006, .991	0.067, .683
PCC	0.06 (−1.30, 1.00)	0.49 (−0.22, 1.14)	0.56 (−0.13, 1.33)	−0.060, .908	−0.070, .991	−0.009, .943
ISTH	−0.67 (−1.59, 0.71)	−1.34 (−1.58, −0.81)	−1.38 (−1.63, −0.52)	0.099, .885	0.110, .991	0.011, .915
INS	1.78 (0.22, 2.34)	0.42 (−0.21, 1.05)	−0.11 (−0.52, 0.48)	0.205, .827	0.287, .377	0.082, .683
TPOL	0.00 (−1.74, 0.00)	0.00 (0.00, 0.00)	−0.58 (−0.61, 0.00)	0.000, 1.000	0.087, .991	0.087, .683
ENTH	0.00 (−1.88, 0.87)	0.00 (−1.52, 0.00)	1.15 (0.00, 1.15)	0.000, 1.000	−0.173, .991	−0.173, .453
PHG	−1.01 (−1.69, 0.98)	0.00 (−1.78, 0.01)	−0.58 (−1.04, −0.43)	−0.153, .908	−0.066, .991	0.087, .683
TVTG	−0.29 (−0.80, 0.74)	−0.69 (−1.12, −0.09)	−0.11 (−0.71, 0.27)	0.060, .908	−0.030, .991	−0.089, .453
ITG	0.12 (−0.81, 1.50)	−0.64 (−1.09, −0.10)	−0.20 (−0.77, 0.37)	0.114, .827	0.047, .991	−0.067, .683
MTG	−0.11 (−0.75, 0.98)	0.38 (−0.21, 0.84)	−0.36 (−0.71, 0.12)	−0.074, .908	0.036, .991	0.110, .329
STG	1.74 (0.35, 2.50)	0.90 (0.40, 1.29)	0.31 (−0.14, 0.88)	0.121, .827	0.214, .715	0.093, .453
BANKS	−1.14 (−1.52, 0.30)	−1.20 (−1.33, −0.89)	−1.12 (−1.33, −0.85)	0.010, .991	−0.002, .991	−0.012, .896
FUSI	0.07 (−0.96, 1.91)	0.17 (−0.44, 0.93)	−0.01 (−0.77, 0.77)	−0.009, .991	0.013, .991	0.023, .917

POSG	0.89 (−0.36, 1.82)	−0.12 (−0.63, 0.65)	−0.10 (−0.62, 0.54)	0.155, .827	0.150, .977	−0.006, .944
PREC	0.35 (−0.96, 1.30)	1.46 (0.51, 1.97)	1.78 (1.11, 2.51)	−0.171, .827	−0.218, .977	−0.047, .896
SMG	0.00 (−0.82, 0.95)	−0.39 (−0.87, 0.29)	−0.01 (−0.51, 0.48)	0.058, .908	−0.001, .991	−0.059, .683
IPL	−0.84 (−1.22, 0.88)	0.05 (−0.48, 0.59)	0.14 (−0.34, 0.68)	−0.132, .827	−0.149, .977	−0.017, .915
SPL	−0.97 (−1.43, 0.28)	−0.79 (−1.22, −0.20)	−0.73 (−1.30, −0.01)	−0.030, .934	−0.036, .991	−0.006, .943
LING	0.61 (−0.87, 1.17)	0.81 (0.00, 1.02)	0.92 (−0.74, 1.10)	−0.032, .917	−0.048, .991	−0.016, .896
CUN	0.56 (−0.62, 1.31)	0.81 (0.00, 0.87)	−0.20 (−0.63, 0.38)	−0.042, .917	0.117, .977	0.159, .056
PERI	0.06 (−1.16, 0.78)	−0.81 (−0.87, 0.00)	0.40 (−1.07, 0.65)	0.135, .827	−0.055, .991	−0.189, .056
LOC	−0.56 (−1.46, 0.61)	−0.81 (−0.89, 0.11)	0.26 (−0.40, 0.62)	0.042, .908	−0.124, .981	−0.166, .153
HIPPO	−0.62 (−1.29, 1.11)	0.29 (−1.07, 1.31)	−0.31 (−0.80, 0.37)	−0.136, .908	−0.049, .991	0.087, .846
AMYG	0.84 (−1.01, 1.35)	−0.14 (−0.54, 0.78)	−0.18 (−0.45, 0.46)	0.151, .827	0.160, .977	0.009, .943
CAUD	−0.68 (−1.52, 0.55)	0.60 (−0.41, 0.81)	−0.13 (−0.48, 0.36)	−0.190, .827	−0.077, .991	0.113, .453
PUT	1.71 (−0.22, 2.02)	0.98 (0.89, 1.92)	1.59 (1.27, 1.84)	0.113, .827	0.019, .991	−0.095, .453
NACC	−0.27 (−1.34, 1.24)	−0.98 (−1.31, −0.26)	0.46 (−0.05, 0.81)	0.103, .827	−0.112, .991	−0.215, .056
PALL	−0.27 (−0.95, 1.13)	−0.60 (−0.82, 0.29)	−1.45 (−1.73, −1.03)	0.048, .908	0.177, .977	0.130, .056
THAL	0.31 (−1.24, 1.07)	−0.14 (−0.74, 0.69)	0.02 (−0.82, 0.45)	0.065, .908	0.035, .991	−0.030, .896

Means were calculated across a range of density thresholds (K = 0.10–0.25, ΔK = 0.01) and are reported with 95% bootstrap confidence intervals (CIs; 5000 resamples). Group differences in area under the curve (ΔAUC) were assessed using permutation testing (10 000 permutations). Abbreviations: SZ+A, schizophrenia with aggression; SZ−A, schizophrenia without aggression; HC, healthy controls; FPOL, frontal pole; POP, pars opercularis; PTRI, pars triangularis; PORB, pars orbitalis; MOFC, medial orbitofrontal cortex; LOFC, lateral orbitofrontal cortex; CMFG, caudal middle frontal gyrus; RMFG, rostral middle frontal gyrus; SFG, superior frontal gyrus; PARL, paracentral lobule; PREG, precentral gyrus; CACC, caudal anterior cingulate cortex; RACC, rostral anterior cingulate cortex; PCC, posterior cingulate cortex; ISTH, isthmus of the cingulate cortex; INS, insular cortex; TPOL, temporal pole; ENTH, entorhinal cortex; PHG, parahippocampal gyrus; TVTG, transverse temporal gyrus; ITG, inferior temporal gyrus; MTG, middle temporal gyrus; STG, superior temporal gyrus; BANKS, banks of the superior temporal sulcus; FUSI, fusiform gyrus; POSG, postcentral gyrus; PREC, precuneus; SMG, supramarginal gyrus; IPL, inferior parietal lobule; SPL, superior parietal lobule; LING, lingual gyrus; CUN, cuneus; PERI, pericalcarine cortex; LOC, lateral occipital cortex; HIPPO, hippocampus; AMYG, amygdala; CAUD, caudate nucleus; PUT, putamen; NACC, nucleus accumbens; PALL, globus pallidus; THAL, thalamus.

Table A3.6. Group differences in participation coefficient.

	M (95% CI)			ΔAUC, p_{BH}		
Node	SZ+A (*N* = 231)	SZ-A (*N* = 2029)	HC (*N* = 3058)	SZ+A vs SZ-A	SZ+A vs HC	SZ-A vs HC
FPOL	0.00 (0.00, 0.16)	0.00 (0.00, 0.00)	0.00 (0.00, 0.00)	0.000, 1.000	0.000, 1.000	0.000, 1.000
POP	0.00 (0.00, 0.28)	0.00 (0.00, 0.20)	0.00 (0.00, 0.00)	0.000, 1.000	0.000, 1.000	0.000, 1.000
PTRI	0.00 (0.00, 0.36)	0.00 (0.00, 0.33)	0.00 (0.00, 0.00)	0.000, 1.000	0.000, 1.000	0.000, 1.000
PORB	0.00 (0.00, 0.22)	0.00 (0.00, 0.45)	0.02 (0.00, 0.16)	0.000, 1.000	−0.001, .968	−0.001, .722
MOFC	0.00 (0.00, 0.41)	0.00 (0.00, 0.05)	0.01 (0.00, 0.11)	0.000, 1.000	−0.001, 1.000	−0.001, 1.000
LOFC	0.04 (0.00, 0.40)	0.00 (0.00, 0.17)	0.06 (0.00, 0.11)	0.005, .762	−0.004, 1.000	−0.009, .722
CMFG	0.01 (0.00, 0.24)	0.00 (0.00, 0.00)	0.00 (0.00, 0.07)	0.001, .562	0.001, .968	0.000, 1.000
RMFG	0.00 (0.00, 0.36)	0.06 (0.00, 0.20)	0.09 (0.00, 0.18)	−0.010, .693	−0.013, .968	−0.003, 1.000
SFG	0.02 (0.00, 0.21)	0.09 (0.01, 0.19)	0.00 (0.00, 0.10)	−0.010, .762	0.003, 1.000	0.013, .722
PARL	0.00 (0.00, 0.21)	0.00 (0.00, 0.00)	0.00 (0.00, 0.00)	0.000, 1.000	0.000, 1.000	0.000, 1.000
PREG	0.02 (0.00, 0.27)	0.00 (0.00, 0.07)	0.00 (0.00, 0.08)	0.002, .822	0.002, .968	0.000, 1.000
CACC	0.13 (0.00, 0.44)	0.00 (0.00, 0.00)	0.00 (0.00, 0.00)	0.019, .138	0.019, .698	0.000, 1.000
RACC	0.21 (0.00, 0.48)	0.00 (0.00, 0.00)	0.02 (0.00, 0.12)	0.031, .138	0.029, .698	−0.002, .351
PCC	0.04 (0.00, 0.47)	0.00 (0.00, 0.03)	0.00 (0.00, 0.10)	0.005, .635	0.005, 1.000	0.000, 1.000
ISTH	0.35 (0.00, 0.53)	0.13 (0.00, 0.38)	0.49 (0.00, 0.50)	0.035, .635	−0.019, 1.000	−0.054, .219
INS	0.11 (0.00, 0.38)	0.00 (0.00, 0.13)	0.00 (0.00, 0.09)	0.017, .635	0.017, .968	0.000, 1.000
TPOL	0.38 (0.00, 0.56)	0.00 (0.00, 0.00)	0.00 (0.00, 0.38)	0.058, .257	0.058, .698	0.000, 1.000
ENTH	0.49 (0.00, 0.58)	0.00 (0.00, 0.20)	0.00 (0.00, 0.50)	0.075, .138	0.075, .698	0.000, 1.000
PHG	0.53 (0.00, 0.63)	0.32 (0.00, 0.55)	0.66 (0.60, 0.72)	0.031, .850	−0.018, 1.000	**−0.050, .002**
TVTG	0.00 (0.00, 0.23)	0.00 (0.00, 0.09)	0.00 (0.00, 0.02)	0.000, 1.000	0.000, 1.000	0.000, 1.000
ITG	0.17 (0.00, 0.63)	0.00 (0.00, 0.04)	0.05 (0.00, 0.24)	0.026, .591	0.019, .968	−0.007, .722
MTG	0.09 (0.00, 0.59)	0.00 (0.00, 0.08)	0.00 (0.00, 0.10)	0.014, .635	0.014, .968	0.000, 1.000
STG	0.08 (0.00, 0.40)	0.00 (0.00, 0.07)	0.00 (0.00, 0.08)	0.012, .635	0.012, .968	0.000, 1.000
BANKS	0.00 (0.00, 0.48)	0.00 (0.00, 0.00)	0.00 (0.00, 0.00)	0.000, 1.000	0.000, 1.000	0.000, 1.000
FUSI	0.51 (0.01, 0.69)	0.30 (0.13, 0.40)	0.33 (0.22, 0.45)	0.032, .635	0.027, .968	−0.005, 1.000

POSG	0.05 (0.00, 0.39)	0.00 (0.00, 0.00)	0.00 (0.00, 0.11)	0.006, .635	0.006, .968	0.000, 1.000
PREC	0.14 (0.00, 0.51)	0.08 (0.01, 0.20)	0.26 (0.00, 0.33)	0.008, .939	−0.018, 1.000	−0.027, .258
SMG	0.00 (0.00, 0.25)	0.00 (0.00, 0.00)	0.00 (0.00, 0.05)	0.000, 1.000	0.000, 1.000	0.000, 1.000
IPL	0.00 (0.00, 0.55)	0.00 (0.00, 0.06)	0.00 (0.00, 0.14)	0.000, 1.000	0.000, 1.000	0.000, 1.000
SPL	0.24 (0.00, 0.47)	0.00 (0.00, 0.16)	0.32 (0.00, 0.43)	0.036, .591	−0.013, 1.000	−0.049, .237
LING	0.02 (0.00, 0.55)	0.07 (0.00, 0.45)	0.11 (0.00, 0.52)	−0.008, 1.000	−0.014, 1.000	−0.006, 1.000
CUN	0.11 (0.00, 0.49)	0.00 (0.00, 0.45)	0.46 (0.00, 0.48)	0.015, .939	−0.055, .968	**−0.070, .015**
PERI	0.00 (0.00, 0.30)	0.00 (0.00, 0.00)	0.00 (0.00, 0.19)	0.000, 1.000	0.000, 1.000	0.000, 1.000
LOC	0.25 (0.00, 0.58)	0.44 (0.40, 0.49)	0.43 (0.00, 0.51)	−0.028, .635	−0.029, .968	−0.001, 1.000
HIPPO	0.00 (0.00, 0.55)	0.32 (0.00, 0.48)	0.35 (0.03, 0.48)	−0.048, .635	−0.053, .698	−0.004, 1.000
AMYG	0.21 (0.00, 0.59)	0.44 (0.00, 0.54)	0.18 (0.00, 0.42)	−0.036, .850	0.006, 1.000	0.042, .722
CAUD	0.00 (0.00, 0.39)	0.02 (0.00, 0.12)	0.00 (0.00, 0.00)	−0.002, .756	0.000, 1.000	0.002, .381
PUT	0.27 (0.00, 0.54)	0.38 (0.00, 0.42)	0.00 (0.00, 0.11)	−0.017, .939	0.042, .968	0.059, .237
NACC	0.49 (0.00, 0.63)	0.11 (0.00, 0.22)	0.00 (0.00, 0.15)	0.057, .366	0.073, .698	0.016, .674
PALL	0.00 (0.00, 0.46)	0.25 (0.00, 0.31)	0.00 (0.00, 0.00)	−0.038, .641	0.000, 1.000	**0.038, .002**
THAL	0.20 (0.00, 0.57)	0.26 (0.00, 0.41)	0.00 (0.00, 0.26)	−0.009, 1.000	0.030, 1.000	0.040, .351

Means were calculated across a range of density thresholds (K = 0.10–0.25, ΔK = 0.01) and are reported with 95% bootstrap confidence intervals (CIs; 5000 resamples). Group differences in area under the curve (ΔAUC) were assessed using permutation testing (10 000 permutations). Bold values indicate statistically significant group differences after Benjamini–Hochberg correction for multiple comparisons ($p_{BH} < .05$). Abbreviations: SZ+A, schizophrenia with aggression; SZ−A, schizophrenia without aggression; HC, healthy controls; FPOL, frontal pole; POP, pars opercularis; PTRI, pars triangularis; PORB, pars orbitalis; MOFC, medial orbitofrontal cortex; LOFC, lateral orbitofrontal cortex; CMFG, caudal middle frontal gyrus; RMFG, rostral middle frontal gyrus; SFG, superior frontal gyrus; PARL, paracentral lobule; PREG, precentral gyrus; CACC, caudal anterior cingulate cortex; RACC, rostral anterior cingulate cortex; PCC, posterior cingulate cortex; ISTH, isthmus of the cingulate cortex; INS, insular cortex; TPOL, temporal pole; ENTH, entorhinal cortex; PHG, parahippocampal gyrus; TVTG, transverse temporal gyrus; ITG, inferior temporal gyrus; MTG, middle temporal gyrus; STG, superior temporal gyrus; BANKS, banks of the superior temporal sulcus; FUSI, fusiform gyrus; POSG, postcentral gyrus; PREC, precuneus; SMG, supramarginal gyrus; IPL, inferior parietal lobule; SPL, superior parietal lobule; LING, lingual gyrus; CUN, cuneus; PERI, pericalcarine cortex; LOC, lateral occipital cortex; HIPPO, hippocampus; AMYG, amygdala; CAUD, caudate nucleus; PUT, putamen; NACC, nucleus accumbens; PALL, globus pallidus; THAL, thalamus.

Table A3.7. Group differences in canonical brain network density.

Metric	M (95% CI)			ΔAUC, p_{BH}		
	SZ+A (*N* = 231)	SZ-A (*N* = 2029)	HC (*N* = 3058)	SZ+A vs SZ-A	SZ+A vs HC	SZ-A vs HC
Salience network						
Network-rest	0.26 (0.17, 0.31)	0.18 (0.14, 0.23)	0.14 (0.12, 0.18)	0.011, .268	0.018, .083	0.006, .281
Central executive network						
Within-network	0.26 (0.06, 0.48)	0.51 (0.36, 0.62)	0.29 (0.17, 0.51)	−0.037, .621	−0.003, 1.000	0.034, .272
Network-rest	0.17 (0.14, 0.23)	0.23 (0.20, 0.26)	0.24 (0.21, 0.26)	−0.009, .268	−0.010, .243	−0.001, .852
Default mode network						
Within-network	0.13 (0.10, 0.20)	0.15 (0.13, 0.18)	0.18 (0.15, 0.20)	−0.003, 1.000	−0.006, 1.000	−0.004, .341
Network-rest	0.15 (0.12, 0.17)	0.15 (0.14, 0.16)	0.15 (0.14, 0.16)	0.000, .960	0.000, .998	0.000, .852
Language network						
Within-network	0.36 (0.24, 0.52)	0.38 (0.30, 0.41)	0.31 (0.30, 0.38)	−0.003, 1.000	0.007, 1.000	0.010, .341
Network-rest	0.17 (0.14, 0.22)	0.20 (0.17, 0.23)	0.16 (0.14, 0.18)	−0.005, .521	0.001, .998	0.006, .160
Reward network						
Within-network	0.39 (0.25, 0.46)	0.28 (0.22, 0.34)	0.39 (0.33, 0.43)	0.016, .621	0.000, 1.000	−0.016, .081
Network-rest	0.14 (0.12, 0.17)	0.15 (0.13, 0.17)	0.14 (0.12, 0.15)	−0.002, .675	0.001, .998	0.003, .348
Affective control network						
Within-network	0.33 (0.25, 0.38)	0.32 (0.28, 0.36)	0.32 (0.28, 0.34)	0.001, 1.000	0.001, 1.000	0.000, 1.000
Network-rest	0.14 (0.13, 0.17)	0.16 (0.15, 0.17)	0.14 (0.14, 0.15)	−0.003, .217	0.000, .998	**0.003, .023**

Means were calculated across a range of density thresholds (*K* = 0.10–0.25, Δ*K* = 0.01) and are reported with 95% bootstrap confidence intervals (CIs; 5000 resamples). Group differences in area under the curve (ΔAUC) were assessed using permutation testing (10 000 permutations). Bold values indicate statistically significant group differences after Benjamini–Hochberg correction for multiple comparisons (p_{BH} < .05). Abbreviations: SZ+A, schizophrenia with aggression; SZ−A, schizophrenia without aggression; HC, healthy controls.

Appendix 4

Exploratory subgroup analyses

Table A4.1. Group differences in global network topology by illness stage.

(a) Early-course schizophrenia

	M (95% CI)			ΔAUC, p_{BH}		
Metric	SZ+A (*N* = 162)	SZ-A (*N* = 964)	HC (*N* = 3058)	SZ+A vs SZ-A	SZ+A vs HC	SZ-A vs HC
Transitivity	0.45 (0.39, 0.51)	0.56 (0.50, 0.59)	0.50 (0.46, 0.52)	−0.017, .075	−0.008, .856	**0.009, .037**
Modularity	0.37 (0.30, 0.42)	0.28 (0.25, 0.32)	0.39 (0.36, 0.42)	**0.014, .023**	−0.003, .856	**−0.017, < .001**
Efficiency	0.48 (0.44, 0.51)	0.38 (0.35, 0.44)	0.45 (0.44, 0.48)	0.015, .051	0.004, .856	**−0.011, .004**
Small-worldness	1.49 (1.30, 1.80)	1.13 (1.06, 1.27)	1.47 (1.33, 1.65)	**0.054, .023**	0.005, .856	**−0.048, .019**

(b) Chronic schizophrenia

	M (95% CI)			ΔAUC, p_{BH}		
Metric	SZ+A (*N* = 60)	SZ-A (*N* = 892)	HC (*N* = 3058)	SZ+A vs SZ-A	SZ+A vs HC	SZ-A vs HC
Transitivity	0.40 (0.37, 0.54)	0.50 (0.45, 0.54)	0.50 (0.46, 0.52)	−0.016, .246	−0.015, .615	0.001, .931
Modularity	0.38 (0.27, 0.45)	0.33 (0.28, 0.35)	0.39 (0.36, 0.42)	0.007, .359	−0.003, .953	−0.010, .147
Efficiency	0.50 (0.43, 0.51)	0.45 (0.42, 0.48)	0.45 (0.44, 0.48)	0.008, .246	0.006, .615	−0.001, .855
Small-worldness	1.57 (1.23, 2.06)	1.39 (1.23, 1.51)	1.47 (1.33, 1.65)	0.027, .314	0.017, .615	−0.010, .855

Means were calculated across a range of density thresholds (K = 0.10–0.25, ΔK = 0.01) and are reported with 95% bootstrap confidence intervals (CIs; 5000 resamples). Group differences in area under the curve (ΔAUC) were assessed using permutation testing (10 000 permutations). Bold values indicate statistically significant group differences after Benjamini–Hochberg correction for multiple comparisons ($p_{BH} < .05$). Abbreviations: SZ+A, schizophrenia with aggression; SZ−A, schizophrenia without aggression; HC, healthy controls.

Table A4.2. Group differences in salience network-to-rest-of-brain density by delusion severity.

(a) Low-severity delusions

M (95% CI)			ΔAUC, *p*		
SZ+A (*N* = 31)	SZ-A (*N* = 1149)	HC (*N* = 3058)	SZ+A vs SZ-A	SZ+A vs HC	SZ-A vs HC
0.18 (0.07, 0.26)	0.19 (0.15, 0.24)	0.14 (0.12, 0.18)	−0.002, .822	0.006, .580	0.008, .104

(b) High-severity delusions

M (95% CI)			ΔAUC, *p*		
SZ+A (*N* = 169)	SZ-A (*N* = 739)	HC (*N* = 3058)	SZ+A vs SZ-A	SZ+A vs HC	SZ-A vs HC
0.28 (0.18, 0.34)	0.20 (0.14, 0.28)	0.14 (0.12, 0.18)	0.012, .237	**0.021, .007**	0.009, .093

Means were calculated across a range of density thresholds (K = 0.10–0.25, ΔK = 0.01) and are reported with 95% bootstrap confidence intervals (CIs; 5000 resamples). Group differences in area under the curve (ΔAUC) were assessed using permutation testing (10 000 permutations). Bold values indicate statistically significant group differences ($p < .05$). Abbreviations: SZ+A, schizophrenia with aggression; SZ−A, schizophrenia without aggression; HC, healthy controls.

Appendix 5

Sensitivity analyses

Table A5.1. Group differences in global network topology using a mild aggression cut-off.

	M (95% CI)			ΔAUC, p_{BH}		
Metric	SZ+A (*N* = 429)	SZ-A (*N* = 1831)	HC (*N* = 3058)	SZ+A vs SZ-A	SZ+A vs HC	SZ-A vs HC
Transitivity	0.50 (0.43, 0.54)	0.56 (0.50, 0.58)	0.50 (0.46, 0.52)	−0.009, .233	−0.001, .944	**0.008, .028**
Modularity	0.35 (0.30, 0.39)	0.30 (0.27, 0.33)	0.39 (0.36, 0.42)	0.008, .095	−0.006, .944	**−0.014, .001**
Efficiency	0.46 (0.42, 0.50)	0.39 (0.37, 0.44)	0.45 (0.44, 0.48)	0.011, .095	0.002, .944	**−0.010, .002**
Small-worldness	1.41 (1.26, 1.64)	1.18 (1.12, 1.30)	1.47 (1.33, 1.65)	0.037, .095	−0.005, .944	**−0.041, .020**

Means were calculated across a range of density thresholds (K = 0.10–0.25, ΔK = 0.01) and are reported with 95% bootstrap confidence intervals (CIs; 5000 resamples). Group differences in area under the curve (ΔAUC) were assessed using permutation testing (10 000 permutations). Bold values indicate statistically significant group differences after Benjamini–Hochberg correction for multiple comparisons (p_{BH} < .05). Abbreviations: SZ+A, schizophrenia with aggression; SZ−A, schizophrenia without aggression; HC, healthy controls.

Table A5.2. Group differences in degree using a mild aggression cut-off.

	M (95% CI)			ΔAUC, p_{BH}		
Node	SZ+A (*N* = 429)	SZ-A (*N* = 1831)	HC (*N* = 3058)	SZ+A vs SZ-A	SZ+A vs HC	SZ-A vs HC
FPOL	1.1 (0.0, 2.8)	0.0 (0.0, 0.6)	1.0 (0.0, 2.1)	0.170, .700	0.026, .914	−0.145, .407
POP	3.4 (1.6, 8.1)	3.8 (1.7, 7.4)	3.9 (2.4, 6.4)	−0.051, .967	−0.079, .914	−0.029, .986
PTRI	5.4 (2.6, 8.1)	4.3 (2.9, 6.2)	5.1 (3.3, 6.1)	0.170, .789	0.045, .914	−0.125, .755
PORB	3.5 (2.1, 5.9)	4.6 (3.2, 6.2)	4.7 (3.4, 7.2)	−0.176, .753	−0.180, .914	−0.005, .986
MOFC	10.1 (5.4, 15.1)	12.1 (8.7, 14.8)	9.0 (6.5, 11.2)	−0.296, .789	0.166, .914	0.463, .445
LOFC	12.2 (9.0, 16.0)	18.7 (14.3, 20.9)	18.2 (13.4, 20.4)	−0.977, .700	−0.918, .914	0.059, .986
CMFG	5.6 (3.0, 9.3)	7.8 (5.5, 9.9)	8.2 (6.8, 10.0)	−0.345, .753	−0.384, .914	−0.040, .986
RMFG	16.9 (11.2, 19.5)	17.3 (12.8, 20.2)	14.9 (10.5, 17.8)	−0.056, .967	0.313, .914	0.370, .755
SFG	15.8 (12.7, 18.4)	20.9 (15.8, 23.8)	17.9 (13.6, 21.3)	−0.760, .702	−0.324, .914	0.436, .739
PARL	6.5 (3.0, 9.5)	7.5 (4.9, 9.8)	8.1 (5.4, 10.4)	−0.155, .914	−0.231, .914	−0.076, .969
PREG	8.4 (5.9, 13.4)	11.1 (7.2, 15.2)	9.3 (6.9, 12.4)	−0.409, .789	−0.140, .914	0.269, .755
CACC	4.0 (2.3, 7.2)	2.6 (2.0, 3.6)	3.1 (2.1, 4.4)	0.214, .702	0.135, .914	−0.079, .739
RACC	8.1 (5.1, 11.4)	11.6 (9.0, 13.4)	8.5 (6.4, 11.2)	−0.530, .702	−0.056, .914	0.474, .407
PCC	13.8 (8.6, 17.1)	10.9 (7.2, 14.1)	10.8 (7.9, 14.8)	0.420, .753	0.436, .914	0.016, .986
ISTH	6.6 (2.9, 11.4)	2.7 (1.2, 5.6)	3.6 (2.6, 6.6)	0.596, .702	0.456, .914	−0.140, .755
INS	14.6 (9.4, 18.2)	11.6 (8.0, 15.2)	7.9 (6.2, 10.7)	0.462, .753	1.010, .517	0.549, .353
TPOL	1.6 (0.0, 3.2)	0.0 (0.0, 0.5)	1.0 (0.9, 1.9)	0.235, .700	0.085, .914	−0.150, .054
ENTH	1.1 (0.0, 2.7)	0.2 (0.0, 1.2)	2.0 (1.9, 2.6)	0.135, .702	−0.130, .914	**−0.265, .004**
PHG	3.1 (1.3, 5.3)	1.7 (1.0, 3.1)	3.4 (2.9, 5.4)	0.210, .702	−0.049, .914	−0.260, .055
TVTG	6.9 (3.6, 10.5)	6.2 (3.9, 9.2)	8.2 (5.6, 9.8)	0.126, .914	−0.179, .914	−0.305, .594
ITG	9.2 (5.4, 13.3)	6.4 (3.7, 8.8)	8.1 (5.4, 11.1)	0.440, .748	0.179, .914	−0.260, .739
MTG	7.8 (5.3, 12.6)	11.8 (8.5, 14.0)	6.9 (5.4, 9.1)	−0.622, .702	0.114, .914	0.736, .054
STG	16.7 (12.1, 19.7)	13.4 (10.6, 15.6)	9.8 (7.8, 12.4)	0.479, .702	1.040, .406	0.561, .198
BANKS	3.6 (2.1, 5.5)	3.4 (3.0, 4.8)	3.6 (3.0, 4.7)	0.045, .914	0.015, .918	−0.030, .969
FUSI	12.8 (7.1, 16.8)	12.2 (8.8, 16.0)	10.7 (6.8, 14.6)	0.105, .967	0.320, .914	0.214, .937

POSG	11.3 (6.9, 14.3)	9.2 (5.8, 13.1)	8.2 (5.9, 11.2)	0.335, .789	0.460, .914	0.125, .969
PREC	13.7 (8.6, 17.3)	17.2 (11.4, 20.4)	18.9 (15.2, 21.0)	−0.552, .753	−0.808, .914	−0.256, .755
SMG	7.2 (3.4, 11.2)	8.1 (5.2, 11.1)	8.6 (6.2, 10.6)	−0.134, .914	−0.201, .914	−0.067, .969
IPL	7.3 (5.4, 10.9)	9.7 (6.5, 12.5)	9.2 (7.0, 11.7)	−0.349, .753	−0.284, .914	0.065, .969
SPL	7.5 (4.0, 10.8)	4.9 (2.7, 8.8)	6.8 (4.2, 9.8)	0.385, .753	0.123, .914	−0.262, .755
LING	4.6 (2.5, 5.1)	3.2 (2.9, 3.9)	4.4 (4.0, 5.8)	0.220, .700	0.045, .914	−0.175, .054
CUN	4.6 (3.1, 6.9)	3.0 (3.0, 3.9)	5.1 (4.1, 6.0)	0.235, .702	−0.080, .914	−0.315, .055
PERI	2.4 (2.0, 3.8)	2.1 (2.0, 2.7)	3.4 (2.9, 3.9)	0.055, .748	−0.154, .914	**−0.210, .004**
LOC	3.8 (2.2, 7.3)	6.2 (4.6, 8.9)	6.1 (4.4, 8.8)	−0.359, .702	−0.340, .914	0.019, .986
HIPPO	3.1 (2.6, 6.6)	3.1 (2.1, 5.3)	4.4 (3.4, 5.5)	−0.010, .967	−0.215, .914	−0.205, .407
AMYG	5.2 (2.5, 9.5)	3.0 (2.0, 5.4)	4.2 (3.2, 6.2)	0.324, .702	0.139, .914	−0.185, .606
CAUD	2.9 (1.8, 3.8)	2.8 (2.0, 3.6)	3.6 (2.8, 4.4)	0.015, .967	−0.110, .914	−0.125, .129
PUT	4.5 (3.2, 6.7)	4.5 (3.3, 5.5)	5.8 (4.9, 6.4)	−0.006, .967	−0.201, .914	−0.195, .407
NACC	3.4 (1.4, 6.2)	1.8 (1.1, 2.8)	4.4 (3.3, 5.2)	0.251, .700	−0.139, .914	**−0.390, .004**
PALL	2.9 (2.0, 4.7)	2.6 (2.0, 3.0)	2.0 (1.6, 2.0)	0.060, .847	0.145, .914	**0.085, .004**
THAL	3.6 (1.4, 6.9)	2.9 (1.6, 5.2)	3.9 (2.4, 5.6)	0.099, .914	−0.051, .914	−0.149, .755

Means were calculated across a range of density thresholds (K = 0.10–0.25, ΔK = 0.01) and are reported with 95% bootstrap confidence intervals (CIs; 5000 resamples). Group differences in area under the curve (ΔAUC) were assessed using permutation testing (10 000 permutations). Bold values indicate statistically significant group differences after Benjamini–Hochberg correction for multiple comparisons (p_{BH} < .05). Abbreviations: SZ+A, schizophrenia with aggression; SZ−A, schizophrenia without aggression; HC, healthy controls; FPOL, frontal pole; POP, pars opercularis; PTRI, pars triangularis; PORB, pars orbitalis; MOFC, medial orbitofrontal cortex; LOFC, lateral orbitofrontal cortex; CMFG, caudal middle frontal gyrus; RMFG, rostral middle frontal gyrus; SFG, superior frontal gyrus; PARL, paracentral lobule; PREG, precentral gyrus; CACC, caudal anterior cingulate cortex; RACC, rostral anterior cingulate cortex; PCC, posterior cingulate cortex; ISTH, isthmus of the cingulate cortex; INS, insular cortex; TPOL, temporal pole; ENTH, entorhinal cortex; PHG, parahippocampal gyrus; TVTG, transverse temporal gyrus; ITG, inferior temporal gyrus; MTG, middle temporal gyrus; STG, superior temporal gyrus; BANKS, banks of the superior temporal sulcus; FUSI, fusiform gyrus; POSG, postcentral gyrus; PREC, precuneus; SMG, supramarginal gyrus; IPL, inferior parietal lobule; SPL, superior parietal lobule; LING, lingual gyrus; CUN, cuneus; PERI, pericalcarine cortex; LOC, lateral occipital cortex; HIPPO, hippocampus; AMYG, amygdala; CAUD, caudate nucleus; PUT, putamen; NACC, nucleus accumbens; PALL, globus pallidus; THAL, thalamus.

Table A5.3. Group differences in betweenness centrality using a mild aggression cut-off.

	M (95% CI)			ΔAUC, p_{BH}		
Node	SZ+A (N = 428)	SZ-A (N = 1831)	HC (N = 3058)	SZ+A vs SZ-A	SZ+A vs HC	SZ-A vs HC
FPOL	0.000 (0.000, 0.000)	0.000 (0.000, 0.000)	0.000 (0.000, 0.000)	0.00000, 1.000	0.00000, 1.000	0.00000, 1.000
POP	0.002 (0.000, 0.015)	0.001 (0.000, 0.011)	0.001 (0.000, 0.007)	0.00002, 1.000	0.00012, .948	0.00010, .950
PTRI	0.009 (0.002, 0.035)	0.008 (0.000, 0.028)	0.004 (0.001, 0.022)	0.00016, 1.000	0.00068, .948	0.00052, .959
PORB	0.001 (0.000, 0.029)	0.015 (0.000, 0.036)	0.002 (0.000, 0.027)	−0.00188, .803	−0.00017, .948	0.00171, .553
MOFC	0.015 (0.001, 0.071)	0.008 (0.003, 0.036)	0.018 (0.002, 0.043)	0.00105, .873	−0.00052, .948	−0.00157, .816
LOFC	0.039 (0.019, 0.128)	0.065 (0.026, 0.136)	0.117 (0.037, 0.192)	−0.00361, .873	−0.01144, .948	−0.00783, .685
CMFG	0.001 (0.000, 0.014)	0.001 (0.000, 0.006)	0.016 (0.002, 0.028)	−0.00000, 1.000	−0.00205, .700	−0.00205, .117
RMFG	0.100 (0.036, 0.170)	0.071 (0.025, 0.140)	0.069 (0.022, 0.131)	0.00435, .873	0.00473, .948	0.00038, 1.000
SFG	0.069 (0.028, 0.130)	0.198 (0.038, 0.311)	0.081 (0.030, 0.210)	−0.01882, .770	−0.00115, .958	0.01768, .540
PARL	0.002 (0.000, 0.011)	0.001 (0.000, 0.004)	0.004 (0.001, 0.012)	0.00019, .873	−0.00033, .948	−0.00052, .318
PREG	0.004 (0.002, 0.044)	0.007 (0.002, 0.076)	0.011 (0.004, 0.068)	−0.00041, 1.000	−0.00085, .948	−0.00045, 1.000
CACC	0.000 (0.000, 0.007)	0.000 (0.000, 0.000)	0.000 (0.000, 0.000)	0.00003, .803	0.00003, .948	0.00000, 1.000
RACC	0.007 (0.002, 0.067)	0.017 (0.009, 0.032)	0.011 (0.005, 0.040)	−0.00140, .873	−0.00054, .948	0.00086, .817
PCC	0.051 (0.015, 0.146)	0.014 (0.007, 0.037)	0.036 (0.012, 0.119)	0.00536, .671	0.00203, .948	−0.00333, .540
ISTH	0.033 (0.002, 0.123)	0.006 (0.000, 0.021)	0.022 (0.007, 0.056)	0.00431, .671	0.00206, .948	−0.00226, .439
INS	0.086 (0.014, 0.205)	0.011 (0.004, 0.116)	0.005 (0.002, 0.052)	0.01114, .731	0.01215, .575	0.00101, .685
TPOL	0.016 (0.000, 0.046)	0.000 (0.000, 0.000)	0.000 (0.000, 0.013)	0.00249, .398	0.00249, .663	0.00000, 1.000
ENTH	0.001 (0.000, 0.034)	0.000 (0.000, 0.018)	0.049 (0.018, 0.050)	0.00015, .873	−0.00726, .563	**−0.00740, .006**
PHG	0.130 (0.000, 0.153)	0.031 (0.000, 0.154)	0.297 (0.131, 0.346)	0.01597, .873	−0.02427, .948	**−0.04025, .041**
TVTG	0.001 (0.000, 0.017)	0.001 (0.000, 0.012)	0.008 (0.002, 0.026)	−0.00002, 1.000	−0.00104, .948	−0.00102, .540
ITG	0.062 (0.007, 0.167)	0.000 (0.000, 0.008)	0.086 (0.006, 0.173)	0.00957, .398	−0.00306, .948	−0.01263, .115
MTG	0.034 (0.006, 0.128)	0.015 (0.008, 0.085)	0.012 (0.004, 0.050)	0.00258, .873	0.00314, .948	0.00055, .959
STG	0.103 (0.034, 0.194)	0.024 (0.016, 0.071)	0.037 (0.017, 0.088)	0.01208, .573	0.01014, .575	−0.00194, .766
BANKS	0.000 (0.000, 0.004)	0.000 (0.000, 0.001)	0.001 (0.000, 0.002)	0.00002, .873	−0.00005, .948	−0.00008, .135
FUSI	0.274 (0.047, 0.312)	0.090 (0.050, 0.249)	0.322 (0.157, 0.382)	0.02928, .731	−0.00666, .956	−0.03594, .115

POSG	0.020 (0.005, 0.055)	0.007 (0.002, 0.020)	0.013 (0.003, 0.035)	0.00202, .731	0.00098, .948	−0.00103, .685
PREC	0.077 (0.024, 0.179)	0.075 (0.036, 0.174)	0.255 (0.128, 0.336)	−0.00029, 1.000	−0.02648, .700	−0.02620, .115
SMG	0.013 (0.001, 0.038)	0.004 (0.001, 0.015)	0.011 (0.004, 0.027)	0.00141, .731	0.00039, .948	−0.00102, .434
IPL	0.022 (0.007, 0.071)	0.011 (0.005, 0.027)	0.029 (0.013, 0.111)	0.00149, .873	−0.00114, .948	−0.00263, .530
SPL	0.024 (0.002, 0.069)	0.001 (0.000, 0.014)	0.019 (0.003, 0.045)	0.00350, .671	0.00121, .948	−0.00230, .439
LING	0.026 (0.001, 0.054)	0.020 (0.006, 0.026)	0.010 (0.002, 0.056)	0.00103, .873	0.00289, .948	0.00186, .439
CUN	0.022 (0.004, 0.079)	0.018 (0.007, 0.032)	0.020 (0.005, 0.039)	0.00030, 1.000	0.00025, .956	−0.00005, 1.000
PERI	0.000 (0.000, 0.003)	0.000 (0.000, 0.000)	0.000 (0.000, 0.001)	0.00000, 1.000	−0.00003, .948	**−0.00003, .048**
LOC	0.044 (0.005, 0.094)	0.112 (0.075, 0.137)	0.051 (0.019, 0.100)	−0.01026, .675	−0.00163, .948	0.00863, .145
HIPPO	0.095 (0.003, 0.125)	0.098 (0.001, 0.168)	0.173 (0.054, 0.228)	−0.00014, 1.000	−0.01123, .948	−0.01108, .670
AMYG	0.072 (0.007, 0.191)	0.027 (0.004, 0.139)	0.104 (0.015, 0.176)	0.00742, .873	−0.00362, .948	−0.01104, .540
CAUD	0.003 (0.000, 0.017)	0.001 (0.000, 0.012)	0.001 (0.001, 0.014)	0.00032, .811	0.00025, .948	−0.00007, 1.000
PUT	0.016 (0.009, 0.097)	0.038 (0.009, 0.092)	0.074 (0.042, 0.092)	−0.00355, .873	−0.00883, .563	−0.00528, .434
NACC	0.042 (0.000, 0.140)	0.001 (0.000, 0.010)	0.013 (0.003, 0.061)	0.00657, .398	0.00470, .948	−0.00187, .685
PALL	0.009 (0.000, 0.053)	0.003 (0.000, 0.031)	0.000 (0.000, 0.000)	0.00082, .962	0.00135, .160	**0.00054, .004**
THAL	0.062 (0.000, 0.145)	0.019 (0.000, 0.114)	0.019 (0.002, 0.115)	0.00684, .873	0.00670, .948	−0.00014, 1.000

Means were calculated across a range of density thresholds (K = 0.10–0.25, ΔK = 0.01) and are reported with 95% bootstrap confidence intervals (CIs; 5000 resamples). Group differences in area under the curve (ΔAUC) were assessed using permutation testing (10 000 permutations). Bold values indicate statistically significant group differences after Benjamini–Hochberg correction for multiple comparisons (p_{BH} < .05). Abbreviations: SZ+A, schizophrenia with aggression; SZ−A, schizophrenia without aggression; HC, healthy controls; FPOL, frontal pole; POP, pars opercularis; PTRI, pars triangularis; PORB, pars orbitalis; MOFC, medial orbitofrontal cortex; LOFC, lateral orbitofrontal cortex; CMFG, caudal middle frontal gyrus; RMFG, rostral middle frontal gyrus; SFG, superior frontal gyrus; PARL, paracentral lobule; PREG, precentral gyrus; CACC, caudal anterior cingulate cortex; RACC, rostral anterior cingulate cortex; PCC, posterior cingulate cortex; ISTH, isthmus of the cingulate cortex; INS, insular cortex; TPOL, temporal pole; ENTH, entorhinal cortex; PHG, parahippocampal gyrus; TVTG, transverse temporal gyrus; ITG, inferior temporal gyrus; MTG, middle temporal gyrus; STG, superior temporal gyrus; BANKS, banks of the superior temporal sulcus; FUSI, fusiform gyrus; POSG, postcentral gyrus; PREC, precuneus; SMG, supramarginal gyrus; IPL, inferior parietal lobule; SPL, superior parietal lobule; LING, lingual gyrus; CUN, cuneus; PERI, pericalcarine cortex; LOC, lateral occipital cortex; HIPPO, hippocampus; AMYG, amygdala; CAUD, caudate nucleus; PUT, putamen; NACC, nucleus accumbens; PALL, globus pallidus; THAL, thalamus.

Table A5.4. Group differences in harmonic centrality using a mild aggression cut-off.

	M (95% CI)			ΔAUC, p_{BH}		
Node	SZ+A (*N* = 429)	SZ-A (*N* = 1831)	HC (*N* = 3058)	SZ+A vs SZ-A	SZ+A vs HC	SZ-A vs HC
FPOL	0.268 (0.000, 0.433)	0.000 (0.000, 0.210)	0.204 (0.000, 0.377)	0.041, .458	0.010, .908	−0.030, .359
POP	0.409 (0.317, 0.507)	0.385 (0.305, 0.475)	0.412 (0.359, 0.472)	0.004, .938	−0.000, .997	−0.004, .781
PTRI	0.460 (0.360, 0.513)	0.397 (0.348, 0.457)	0.441 (0.393, 0.469)	0.010, .483	0.003, .908	−0.007, .306
PORB	0.422 (0.378, 0.475)	0.420 (0.395, 0.467)	0.445 (0.423, 0.496)	0.000, .974	−0.003, .908	−0.004, .431
MOFC	0.558 (0.472, 0.630)	0.530 (0.492, 0.583)	0.520 (0.480, 0.562)	0.005, .738	0.006, .908	0.001, .844
LOFC	0.588 (0.531, 0.646)	0.621 (0.571, 0.675)	0.655 (0.590, 0.696)	−0.005, .738	−0.010, .908	−0.006, .486
CMFG	0.468 (0.415, 0.529)	0.467 (0.440, 0.509)	0.495 (0.479, 0.538)	0.000, .974	−0.004, .908	−0.004, .359
RMFG	0.649 (0.567, 0.690)	0.609 (0.554, 0.665)	0.602 (0.539, 0.654)	0.006, .722	0.007, .908	0.001, .900
SFG	0.623 (0.577, 0.672)	0.662 (0.592, 0.730)	0.648 (0.592, 0.711)	−0.005, .738	−0.003, .908	0.002, .844
PARL	0.478 (0.406, 0.538)	0.458 (0.424, 0.511)	0.508 (0.465, 0.547)	0.003, .824	−0.004, .908	−0.008, .254
PREG	0.511 (0.461, 0.593)	0.518 (0.467, 0.596)	0.527 (0.492, 0.580)	−0.001, .974	−0.002, .908	−0.002, .844
CACC	0.435 (0.390, 0.508)	0.363 (0.337, 0.407)	0.406 (0.370, 0.440)	0.011, .458	0.004, .908	−0.007, .207
RACC	0.522 (0.457, 0.582)	0.522 (0.489, 0.565)	0.508 (0.475, 0.565)	0.000, .974	0.003, .908	0.002, .790
PCC	0.608 (0.528, 0.662)	0.511 (0.466, 0.570)	0.553 (0.505, 0.622)	0.015, .458	0.008, .908	−0.007, .471
ISTH	0.489 (0.405, 0.581)	0.387 (0.347, 0.455)	0.448 (0.425, 0.498)	0.016, .458	0.007, .908	−0.009, .128
INS	0.620 (0.538, 0.684)	0.523 (0.479, 0.601)	0.505 (0.482, 0.560)	0.015, .458	0.018, .683	0.003, .768
TPOL	0.281 (0.000, 0.424)	0.000 (0.000, 0.113)	0.245 (0.226, 0.352)	0.043, .458	0.006, .908	**−0.037, .031**
ENTH	0.208 (0.000, 0.406)	0.076 (0.000, 0.265)	0.320 (0.300, 0.377)	0.021, .722	−0.016, .908	**−0.037, .031**
PHG	0.412 (0.232, 0.475)	0.353 (0.300, 0.413)	0.434 (0.405, 0.481)	0.010, .483	−0.002, .908	**−0.012, .033**
TVTG	0.493 (0.438, 0.555)	0.446 (0.408, 0.512)	0.517 (0.470, 0.547)	0.008, .603	−0.003, .908	−0.011, .117
ITG	0.543 (0.462, 0.616)	0.452 (0.404, 0.506)	0.529 (0.471, 0.589)	0.014, .458	0.002, .908	−0.012, .117
MTG	0.526 (0.462, 0.608)	0.526 (0.485, 0.580)	0.493 (0.457, 0.541)	−0.000, .994	0.005, .908	0.005, .486
STG	0.651 (0.581, 0.702)	0.549 (0.521, 0.601)	0.538 (0.506, 0.585)	0.016, .458	0.017, .656	0.002, .792
BANKS	0.431 (0.367, 0.484)	0.370 (0.361, 0.430)	0.408 (0.390, 0.448)	0.010, .483	0.004, .908	−0.006, .245
FUSI	0.612 (0.498, 0.676)	0.547 (0.500, 0.623)	0.574 (0.516, 0.643)	0.011, .483	0.006, .908	−0.005, .728

POSG	0.557 (0.484, 0.610)	0.487 (0.444, 0.557)	0.520 (0.478, 0.567)	0.011, .483	0.006, .908	−0.005, .486
PREC	0.615 (0.530, 0.668)	0.608 (0.540, 0.673)	0.676 (0.632, 0.716)	0.001, .974	−0.009, .908	−0.010, .175
SMG	0.502 (0.412, 0.560)	0.465 (0.428, 0.530)	0.520 (0.484, 0.557)	0.006, .727	−0.002, .908	−0.008, .198
IPL	0.516 (0.467, 0.579)	0.499 (0.456, 0.558)	0.538 (0.507, 0.585)	0.003, .872	−0.003, .908	−0.006, .339
SPL	0.500 (0.428, 0.567)	0.424 (0.385, 0.500)	0.490 (0.447, 0.545)	0.012, .483	0.002, .908	−0.010, .245
LING	0.409 (0.288, 0.451)	0.333 (0.311, 0.373)	0.401 (0.372, 0.467)	0.012, .458	0.002, .908	−0.010, .093
CUN	0.405 (0.309, 0.487)	0.329 (0.311, 0.395)	0.446 (0.391, 0.480)	0.012, .483	−0.006, .908	**−0.018, .033**
PERI	0.326 (0.255, 0.396)	0.258 (0.242, 0.327)	0.362 (0.338, 0.392)	0.011, .483	−0.005, .908	**−0.016, .029**
LOC	0.437 (0.326, 0.514)	0.464 (0.421, 0.525)	0.478 (0.417, 0.541)	−0.004, .840	−0.006, .908	−0.003, .781
HIPPO	0.360 (0.269, 0.497)	0.255 (0.139, 0.449)	0.377 (0.335, 0.453)	0.017, .722	−0.002, .920	−0.019, .135
AMYG	0.400 (0.281, 0.553)	0.222 (0.142, 0.427)	0.366 (0.310, 0.468)	0.028, .483	0.006, .908	−0.022, .117
CAUD	0.292 (0.208, 0.382)	0.180 (0.121, 0.290)	0.278 (0.254, 0.347)	0.018, .483	0.003, .908	−0.015, .117
PUT	0.344 (0.250, 0.477)	0.229 (0.146, 0.354)	0.346 (0.309, 0.406)	0.018, .483	0.000, .997	−0.018, .117
NACC	0.365 (0.217, 0.495)	0.170 (0.109, 0.287)	0.323 (0.286, 0.400)	0.031, .458	0.007, .908	**−0.023, .033**
PALL	0.307 (0.212, 0.397)	0.182 (0.120, 0.298)	0.258 (0.230, 0.291)	0.020, .483	0.008, .908	−0.012, .117
THAL	0.380 (0.227, 0.514)	0.209 (0.132, 0.410)	0.328 (0.290, 0.439)	0.027, .483	0.009, .908	−0.018, .245

Means were calculated across a range of density thresholds (K = 0.10–0.25, ΔK = 0.01) and are reported with 95% bootstrap confidence intervals (CIs; 5000 resamples). Group differences in area under the curve (ΔAUC) were assessed using permutation testing (10 000 permutations). Bold values indicate statistically significant group differences after Benjamini–Hochberg correction for multiple comparisons ($p_{BH} < .05$). Abbreviations: SZ+A, schizophrenia with aggression; SZ−A, schizophrenia without aggression; HC, healthy controls; FPOL, frontal pole; POP, pars opercularis; PTRI, pars triangularis; PORB, pars orbitalis; MOFC, medial orbitofrontal cortex; LOFC, lateral orbitofrontal cortex; CMFG, caudal middle frontal gyrus; RMFG, rostral middle frontal gyrus; SFG, superior frontal gyrus; PARL, paracentral lobule; PREG, precentral gyrus; CACC, caudal anterior cingulate cortex; RACC, rostral anterior cingulate cortex; PCC, posterior cingulate cortex; ISTH, isthmus of the cingulate cortex; INS, insular cortex; TPOL, temporal pole; ENTH, entorhinal cortex; PHG, parahippocampal gyrus; TVTG, transverse temporal gyrus; ITG, inferior temporal gyrus; MTG, middle temporal gyrus; STG, superior temporal gyrus; BANKS, banks of the superior temporal sulcus; FUSI, fusiform gyrus; POSG, postcentral gyrus; PREC, precuneus; SMG, supramarginal gyrus; IPL, inferior parietal lobule; SPL, superior parietal lobule; LING, lingual gyrus; CUN, cuneus; PERI, pericalcarine cortex; LOC, lateral occipital cortex; HIPPO, hippocampus; AMYG, amygdala; CAUD, caudate nucleus; PUT, putamen; NACC, nucleus accumbens; PALL, globus pallidus; THAL, thalamus.

Table A5.5. Group differences in within-module degree using a mild aggression cut-off.

Node	M (95% CI)			ΔAUC, p_{BH}		
	SZ+A (*N* = 429)	SZ-A (*N* = 1831)	HC (*N* = 3058)	SZ+A vs SZ-A	SZ+A vs HC	SZ-A vs HC
FPOL	−1.66 (−1.87, 0.00)	0.00 (−1.56, 0.00)	−1.76 (−1.87, 0.00)	−0.248, .988	0.015, .934	0.263, .320
POP	−1.14 (−1.53, 0.00)	−1.05 (−1.42, −0.11)	−1.07 (−1.46, −0.50)	−0.013, 1.000	−0.012, .934	0.001, .993
PTRI	−0.69 (−1.24, 1.08)	−0.92 (−1.21, 0.36)	−0.78 (−1.24, −0.50)	0.037, .988	0.016, .934	−0.022, .986
PORB	−1.10 (−1.48, −0.44)	−0.85 (−1.18, −0.48)	−0.87 (−1.20, −0.35)	−0.039, .988	−0.037, .934	0.002, .993
MOFC	0.33 (−0.71, 1.49)	0.53 (−0.13, 1.09)	0.08 (−0.50, 0.62)	−0.032, .989	0.035, .934	0.067, .876
LOFC	0.78 (0.01, 1.60)	1.83 (0.99, 2.28)	2.10 (1.13, 2.68)	−0.158, .988	−0.201, .791	−0.043, .986
CMFG	−0.65 (−1.23, 0.17)	−0.23 (−0.73, 0.15)	−0.02 (−0.36, 0.37)	−0.067, .988	−0.094, .791	−0.027, .986
RMFG	1.87 (0.49, 2.36)	1.42 (0.61, 2.01)	1.25 (0.38, 1.90)	0.069, .988	0.095, .934	0.026, .986
SFG	1.64 (1.00, 2.32)	2.05 (1.27, 2.58)	2.22 (1.31, 2.85)	−0.060, .988	−0.085, .934	−0.025, .986
PARL	−0.48 (−1.26, 0.26)	−0.34 (−0.83, 0.13)	−0.11 (−0.67, 0.46)	−0.023, .989	−0.055, .934	−0.032, .986
PREG	−0.04 (−0.56, 1.07)	0.36 (−0.38, 1.13)	0.18 (−0.36, 0.85)	−0.061, .988	−0.035, .934	0.026, .986
CACC	−1.02 (−1.48, −0.28)	−1.24 (−1.51, −1.05)	−1.24 (−1.53, −0.93)	0.033, .988	0.033, .934	0.000, .993
RACC	−0.08 (−0.84, 0.53)	0.51 (−0.03, 0.88)	−0.02 (−0.49, 0.62)	−0.087, .988	−0.008, .934	0.079, .655
PCC	1.13 (−0.06, 1.77)	0.33 (−0.39, 0.96)	0.56 (−0.13, 1.33)	0.118, .988	0.086, .934	−0.032, .986
ISTH	−0.84 (−1.42, 0.48)	−1.35 (−1.62, −0.89)	−1.38 (−1.63, −0.52)	0.079, .988	0.088, .934	0.009, .986
INS	1.21 (0.12, 1.94)	0.40 (−0.28, 1.08)	−0.11 (−0.52, 0.48)	0.122, .988	0.200, .454	0.078, .655
TPOL	0.00 (−1.74, 0.00)	0.00 (0.00, 0.00)	−0.58 (−0.61, 0.00)	0.000, 1.000	0.087, .934	0.087, .651
ENTH	0.00 (−1.83, 1.05)	0.00 (−1.53, 0.00)	1.15 (0.00, 1.15)	0.000, 1.000	−0.173, .934	−0.173, .395
PHG	−1.56 (−1.81, 0.87)	−1.53 (−1.73, 0.00)	−0.58 (−1.04, −0.43)	−0.010, 1.000	−0.154, .710	−0.143, .395
TVTG	−0.37 (−1.10, 0.43)	−0.57 (−1.06, −0.03)	−0.11 (−0.71, 0.27)	0.031, .989	−0.038, .934	−0.070, .655
ITG	−0.14 (−0.81, 0.97)	−0.53 (−1.11, −0.10)	−0.20 (−0.77, 0.37)	0.060, .988	0.010, .934	−0.050, .972
MTG	−0.38 (−0.79, 0.80)	0.53 (−0.13, 0.92)	−0.36 (−0.71, 0.12)	−0.141, .988	−0.007, .934	0.134, .141
STG	1.85 (0.73, 2.37)	0.85 (0.33, 1.25)	0.31 (−0.14, 0.88)	0.148, .988	0.232, .217	0.085, .502
BANKS	−1.08 (−1.47, −0.21)	−1.07 (−1.28, −0.84)	−1.12 (−1.33, −0.85)	0.000, 1.000	0.006, .934	0.006, .986
FUSI	0.27 (−0.94, 1.26)	0.41 (−0.44, 1.02)	−0.01 (−0.77, 0.77)	−0.014, 1.000	0.046, .934	0.060, .986

POSG	0.64 (−0.35, 1.29)	−0.03 (−0.66, 0.73)	−0.10 (−0.62, 0.54)	0.103, .988	0.111, .934	0.008, .986
PREC	0.94 (−0.34, 1.69)	1.43 (0.36, 2.00)	1.78 (1.11, 2.51)	−0.075, .988	−0.127, .934	−0.052, .986
SMG	−0.31 (−1.18, 0.60)	−0.22 (−0.78, 0.39)	−0.01 (−0.51, 0.48)	−0.014, 1.000	−0.046, .934	−0.032, .986
IPL	−0.24 (−0.68, 0.84)	0.10 (−0.53, 0.65)	0.14 (−0.34, 0.68)	−0.052, .988	−0.059, .934	−0.007, .986
SPL	−0.45 (−1.23, 0.32)	−0.82 (−1.30, −0.17)	−0.73 (−1.30, −0.01)	0.054, .988	0.043, .934	−0.011, .986
LING	0.60 (−0.89, 1.00)	0.81 (0.00, 1.14)	0.92 (−0.74, 1.10)	−0.031, .988	−0.047, .934	−0.016, .986
CUN	0.67 (−0.57, 1.22)	0.81 (0.00, 0.87)	−0.20 (−0.63, 0.38)	−0.025, .989	0.134, .710	0.159, .050
PERI	−0.60 (−1.28, 0.35)	−0.81 (−0.87, 0.00)	0.40 (−1.07, 0.65)	0.031, .988	−0.159, .934	−0.189, .050
LOC	−0.67 (−1.28, 0.24)	−0.81 (−0.89, 0.44)	0.26 (−0.40, 0.62)	0.025, .989	−0.141, .710	−0.166, .126
HIPPO	−0.12 (−1.17, 1.36)	0.14 (−1.03, 1.24)	−0.31 (−0.80, 0.37)	−0.036, 1.000	0.027, .934	0.063, .986
AMYG	1.11 (−0.84, 1.58)	−0.07 (−0.58, 0.76)	−0.18 (−0.45, 0.46)	0.177, .988	0.198, .219	0.021, .986
CAUD	−0.25 (−1.00, 0.87)	0.54 (−0.42, 0.81)	−0.13 (−0.48, 0.36)	−0.117, .988	−0.012, .934	0.105, .502
PUT	1.36 (0.46, 1.90)	1.00 (0.89, 1.94)	1.59 (1.27, 1.84)	0.049, .988	−0.042, .934	−0.091, .502
NACC	−0.25 (−1.30, 0.45)	−1.00 (−1.37, −0.33)	0.46 (−0.05, 0.81)	0.115, .988	−0.103, .934	−0.218, .050
PALL	−0.18 (−0.87, 0.93)	−0.54 (−0.84, 0.28)	−1.45 (−1.73, −1.03)	0.058, .988	0.196, .217	0.138, .050
THAL	−0.11 (−1.38, 0.65)	−0.07 (−0.71, 0.76)	0.02 (−0.82, 0.45)	−0.005, 1.000	−0.023, .934	−0.018, .986

Means were calculated across a range of density thresholds ($K = 0.10–0.25$, $\Delta K = 0.01$) and are reported with 95% bootstrap confidence intervals (CIs; 5000 resamples). Group differences in area under the curve (ΔAUC) were assessed using permutation testing (10 000 permutations). Abbreviations: SZ+A, schizophrenia with aggression; SZ−A, schizophrenia without aggression; HC, healthy controls; FPOL, frontal pole; POP, pars opercularis; PTRI, pars triangularis; PORB, pars orbitalis; MOFC, medial orbitofrontal cortex; LOFC, lateral orbitofrontal cortex; CMFG, caudal middle frontal gyrus; RMFG, rostral middle frontal gyrus; SFG, superior frontal gyrus; PARL, paracentral lobule; PREG, precentral gyrus; CACC, caudal anterior cingulate cortex; RACC, rostral anterior cingulate cortex; PCC, posterior cingulate cortex; ISTH, isthmus of the cingulate cortex; INS, insular cortex; TPOL, temporal pole; ENTH, entorhinal cortex; PHG, parahippocampal gyrus; TVTG, transverse temporal gyrus; ITG, inferior temporal gyrus; MTG, middle temporal gyrus; STG, superior temporal gyrus; BANKS, banks of the superior temporal sulcus; FUSI, fusiform gyrus; POSG, postcentral gyrus; PREC, precuneus; SMG, supramarginal gyrus; IPL, inferior parietal lobule; SPL, superior parietal lobule; LING, lingual gyrus; CUN, cuneus; PERI, pericalcarine cortex; LOC, lateral occipital cortex; HIPPO, hippocampus; AMYG, amygdala; CAUD, caudate nucleus; PUT, putamen; NACC, nucleus accumbens; PALL, globus pallidus; THAL, thalamus.

Table A5.6. Group differences in participation coefficient using a mild aggression cut-off.

Node	M (95% CI)			ΔAUC, p_{BH}		
	SZ+A (*N* = 429)	SZ-A (*N* = 1831)	HC (*N* = 3058)	SZ+A vs SZ-A	SZ+A vs HC	SZ-A vs HC
FPOL	0.00 (0.00, 0.00)	0.00 (0.00, 0.00)	0.00 (0.00, 0.00)	0.000, 1.000	0.000, 1.000	0.000, 1.000
POP	0.00 (0.00, 0.31)	0.00 (0.00, 0.18)	0.00 (0.00, 0.00)	0.000, 1.000	0.000, 1.000	0.000, 1.000
PTRI	0.00 (0.00, 0.32)	0.00 (0.00, 0.36)	0.00 (0.00, 0.00)	0.000, 1.000	0.000, 1.000	0.000, 1.000
PORB	0.00 (0.00, 0.48)	0.00 (0.00, 0.41)	0.02 (0.00, 0.16)	0.000, 1.000	−0.001, 1.000	−0.001, .748
MOFC	0.00 (0.00, 0.18)	0.00 (0.00, 0.05)	0.01 (0.00, 0.11)	0.000, 1.000	−0.001, 1.000	−0.001, 1.000
LOFC	0.00 (0.00, 0.28)	0.02 (0.00, 0.15)	0.06 (0.00, 0.11)	−0.002, .896	−0.009, 1.000	−0.007, .974
CMFG	0.00 (0.00, 0.08)	0.00 (0.00, 0.00)	0.00 (0.00, 0.07)	0.000, 1.000	0.000, 1.000	0.000, 1.000
RMFG	0.00 (0.00, 0.29)	0.08 (0.00, 0.19)	0.09 (0.00, 0.18)	−0.013, .640	−0.013, 1.000	−0.000, 1.000
SFG	0.00 (0.00, 0.10)	0.12 (0.01, 0.21)	0.00 (0.00, 0.10)	−0.018, .582	0.000, 1.000	0.018, .336
PARL	0.00 (0.00, 0.04)	0.00 (0.00, 0.00)	0.00 (0.00, 0.00)	0.000, 1.000	0.000, 1.000	0.000, 1.000
PREG	0.00 (0.00, 0.09)	0.00 (0.00, 0.10)	0.00 (0.00, 0.08)	0.000, 1.000	0.000, 1.000	0.000, 1.000
CACC	0.00 (0.00, 0.16)	0.00 (0.00, 0.00)	0.00 (0.00, 0.00)	0.000, 1.000	0.000, 1.000	0.000, 1.000
RACC	0.00 (0.00, 0.25)	0.00 (0.00, 0.01)	0.02 (0.00, 0.12)	0.000, 1.000	−0.002, 1.000	−0.002, .291
PCC	0.01 (0.00, 0.27)	0.00 (0.00, 0.02)	0.00 (0.00, 0.10)	0.000, .727	0.000, 1.000	0.000, 1.000
ISTH	0.31 (0.00, 0.55)	0.15 (0.00, 0.41)	0.49 (0.00, 0.50)	0.027, .896	−0.025, 1.000	−0.051, .218
INS	0.05 (0.00, 0.28)	0.00 (0.00, 0.14)	0.00 (0.00, 0.09)	0.008, .896	0.008, 1.000	0.000, 1.000
TPOL	0.30 (0.00, 0.50)	0.00 (0.00, 0.00)	0.00 (0.00, 0.38)	0.046, .465	0.046, 1.000	0.000, 1.000
ENTH	0.25 (0.00, 0.50)	0.00 (0.00, 0.22)	0.00 (0.00, 0.50)	0.037, .582	0.037, 1.000	0.000, 1.000
PHG	0.52 (0.00, 0.63)	0.26 (0.00, 0.54)	0.66 (0.60, 0.72)	0.043, .727	−0.017, 1.000	**−0.060, .004**
TVTG	0.00 (0.00, 0.07)	0.00 (0.00, 0.11)	0.00 (0.00, 0.02)	0.000, 1.000	0.000, 1.000	0.000, 1.000
ITG	0.17 (0.00, 0.55)	0.00 (0.00, 0.02)	0.05 (0.00, 0.24)	0.025, .582	0.019, 1.000	−0.007, .589
MTG	0.19 (0.00, 0.61)	0.00 (0.00, 0.08)	0.00 (0.00, 0.10)	0.029, .465	0.029, 1.000	0.000, 1.000
STG	0.02 (0.00, 0.30)	0.00 (0.00, 0.08)	0.00 (0.00, 0.08)	0.002, .896	0.002, 1.000	0.000, 1.000
BANKS	0.00 (0.00, 0.32)	0.00 (0.00, 0.00)	0.00 (0.00, 0.00)	0.000, 1.000	0.000, 1.000	0.000, 1.000
FUSI	0.37 (0.12, 0.66)	0.16 (0.01, 0.41)	0.33 (0.22, 0.45)	0.032, .597	0.005, 1.000	−0.026, .136

POSG	0.00 (0.00, 0.12)	0.00 (0.00, 0.02)	0.00 (0.00, 0.11)	0.000, 1.000	0.000, 1.000	0.000, 1.000
PREC	0.08 (0.00, 0.46)	0.08 (0.00, 0.22)	0.26 (0.00, 0.33)	−0.002, 1.000	−0.028, 1.000	−0.026, .223
SMG	0.00 (0.00, 0.23)	0.00 (0.00, 0.00)	0.00 (0.00, 0.05)	0.000, 1.000	0.000, 1.000	0.000, 1.000
IPL	0.00 (0.00, 0.51)	0.00 (0.00, 0.05)	0.00 (0.00, 0.14)	0.000, 1.000	0.000, 1.000	0.000, 1.000
SPL	0.18 (0.00, 0.44)	0.00 (0.00, 0.16)	0.32 (0.00, 0.43)	0.028, .586	−0.021, 1.000	−0.049, .223
LING	0.41 (0.00, 0.49)	0.07 (0.00, 0.46)	0.11 (0.00, 0.52)	0.053, .582	0.047, 1.000	−0.006, 1.000
CUN	0.35 (0.00, 0.49)	0.00 (0.00, 0.46)	0.46 (0.00, 0.48)	0.054, .641	−0.016, 1.000	**−0.070, .018**
PERI	0.07 (0.00, 0.30)	0.00 (0.00, 0.03)	0.00 (0.00, 0.19)	0.009, .582	0.009, 1.000	0.000, 1.000
LOC	0.47 (0.00, 0.54)	0.44 (0.38, 0.50)	0.43 (0.00, 0.51)	0.005, .896	0.003, 1.000	−0.002, 1.000
HIPPO	0.02 (0.00, 0.59)	0.26 (0.00, 0.49)	0.35 (0.03, 0.48)	−0.036, .793	−0.051, 1.000	−0.015, .971
AMYG	0.16 (0.00, 0.62)	0.41 (0.00, 0.53)	0.18 (0.00, 0.42)	−0.039, .896	−0.003, 1.000	0.037, .748
CAUD	0.00 (0.00, 0.17)	0.07 (0.00, 0.14)	0.00 (0.00, 0.00)	−0.009, .582	0.000, 1.000	0.009, .223
PUT	0.00 (0.00, 0.47)	0.37 (0.00, 0.40)	0.00 (0.00, 0.11)	−0.056, .597	0.000, 1.000	0.056, .218
NACC	0.21 (0.00, 0.58)	0.08 (0.00, 0.19)	0.00 (0.00, 0.15)	0.021, .641	0.032, 1.000	0.011, .480
PALL	0.00 (0.00, 0.44)	0.25 (0.00, 0.31)	0.00 (0.00, 0.00)	−0.038, .641	0.000, 1.000	**0.038, .004**
THAL	0.16 (0.00, 0.58)	0.26 (0.00, 0.41)	0.00 (0.00, 0.26)	−0.015, 1.000	0.025, 1.000	0.040, .291

Means were calculated across a range of density thresholds (K = 0.10–0.25, ΔK = 0.01) and are reported with 95% bootstrap confidence intervals (CIs; 5000 resamples). Group differences in area under the curve (ΔAUC) were assessed using permutation testing (10 000 permutations). Bold values indicate statistically significant group differences after Benjamini–Hochberg correction for multiple comparisons (p_{BH} < .05). Abbreviations: SZ+A, schizophrenia with aggression; SZ−A, schizophrenia without aggression; HC, healthy controls; FPOL, frontal pole; POP, pars opercularis; PTRI, pars triangularis; PORB, pars orbitalis; MOFC, medial orbitofrontal cortex; LOFC, lateral orbitofrontal cortex; CMFG, caudal middle frontal gyrus; RMFG, rostral middle frontal gyrus; SFG, superior frontal gyrus; PARL, paracentral lobule; PREG, precentral gyrus; CACC, caudal anterior cingulate cortex; RACC, rostral anterior cingulate cortex; PCC, posterior cingulate cortex; ISTH, isthmus of the cingulate cortex; INS, insular cortex; TPOL, temporal pole; ENTH, entorhinal cortex; PHG, parahippocampal gyrus; TVTG, transverse temporal gyrus; ITG, inferior temporal gyrus; MTG, middle temporal gyrus; STG, superior temporal gyrus; BANKS, banks of the superior temporal sulcus; FUSI, fusiform gyrus; POSG, postcentral gyrus; PREC, precuneus; SMG, supramarginal gyrus; IPL, inferior parietal lobule; SPL, superior parietal lobule; LING, lingual gyrus; CUN, cuneus; PERI, pericalcarine cortex; LOC, lateral occipital cortex; HIPPO, hippocampus; AMYG, amygdala; CAUD, caudate nucleus; PUT, putamen; NACC, nucleus accumbens; PALL, globus pallidus; THAL, thalamus.

Table A5.7. Group differences in canonical brain network density using a mild aggression cut-off.

Metric	M (95% CI)			ΔAUC, p_{BH}		
	SZ+A (*N* = 429)	SZ-A (*N* = 1831)	HC (*N* = 3058)	SZ+A vs SZ-A	SZ+A vs HC	SZ-A vs HC
Salience network						
Network-rest	0.24 (0.17, 0.29)	0.18 (0.14, 0.23)	0.14 (0.12, 0.18)	0.009, .453	0.015, .119	0.006, .260
Central executive network						
Within-network	0.49 (0.20, 0.65)	0.46 (0.30, 0.59)	0.29 (0.17, 0.51)	0.005, 1.000	0.031, .756	0.026, .427
Network-rest	0.21 (0.17, 0.25)	0.23 (0.19, 0.26)	0.24 (0.21, 0.26)	−0.003, .838	−0.004, .665	−0.001, .792
Default mode network						
Within-network	0.16 (0.13, 0.22)	0.15 (0.12, 0.17)	0.18 (0.15, 0.20)	0.001, 1.000	−0.003, .756	−0.004, .427
Network-rest	0.15 (0.13, 0.16)	0.15 (0.14, 0.16)	0.15 (0.14, 0.16)	−0.000, .959	0.000, .899	0.000, .792
Language network						
Within-network	0.32 (0.30, 0.49)	0.37 (0.30, 0.42)	0.31 (0.30, 0.38)	−0.008, 1.000	0.001, 1.000	0.009, .427
Network-rest	0.19 (0.16, 0.23)	0.20 (0.17, 0.23)	0.16 (0.14, 0.18)	−0.001, .953	0.005, .665	0.006, .190
Reward network						
Within-network	0.32 (0.22, 0.39)	0.29 (0.22, 0.35)	0.39 (0.33, 0.43)	0.005, 1.000	−0.010, .756	−0.015, .187
Network-rest	0.12 (0.10, 0.15)	0.16 (0.14, 0.17)	0.14 (0.12, 0.15)	−0.005, .263	−0.002, .665	0.003, .260
Affective control network						
Within-network	0.29 (0.24, 0.35)	0.32 (0.28, 0.36)	0.32 (0.28, 0.34)	−0.005, 1.000	−0.005, .756	0.001, 1.000
Network-rest	0.15 (0.14, 0.17)	0.17 (0.15, 0.17)	0.14 (0.14, 0.15)	−0.002, .263	0.001, .768	**0.003, .016**

Means were calculated across a range of density thresholds (K = 0.10–0.25, ΔK = 0.01) and are reported with 95% bootstrap confidence intervals (CIs; 5000 resamples). Group differences in area under the curve (ΔAUC) were assessed using permutation testing (10 000 permutations). Bold values indicate statistically significant group differences after Benjamini–Hochberg correction for multiple comparisons (p_{BH} < .05). Abbreviations: SZ+A, schizophrenia with aggression; SZ−A, schizophrenia without aggression; HC, healthy controls.

Table A5.8. Group differences in global network topology using PANSS P7 'Hostility' scores only

	M (95% CI)			ΔAUC, p_{BH}		
Metric	SZ+A (*N* = 97)	SZ-A (*N* = 1477)	HC (*N* = 3058)	SZ+A vs SZ-A	SZ+A vs HC	SZ-A vs HC
Transitivity	0.39 (0.36, 0.47)	0.57 (0.50, 0.59)	0.50 (0.46, 0.52)	**−0.026, .019**	−0.016, .358	**0.010, .014**
Modularity	0.37 (0.30, 0.43)	0.31 (0.27, 0.33)	0.39 (0.36, 0.42)	0.009, .148	−0.004, .926	**−0.013, < .001**
Efficiency	0.51 (0.46, 0.52)	0.37 (0.36, 0.44)	0.45 (0.44, 0.48)	**0.021, .004**	0.008, .358	**−0.013, < .001**
Small-worldness	1.52 (1.30, 1.94)	1.16 (1.13, 1.32)	1.47 (1.33, 1.65)	**0.053, .025**	0.009, .926	**−0.044, .014**

Means were calculated across a range of density thresholds (K = 0.10–0.25, ΔK = 0.01) and are reported with 95% bootstrap confidence intervals (CIs; 5000 resamples). Group differences in area under the curve (ΔAUC) were assessed using permutation testing (10 000 permutations). Bold values indicate statistically significant group differences after Benjamini–Hochberg correction for multiple comparisons ($p_{BH} < .05$). Abbreviations: SZ+A, schizophrenia with aggression; SZ−A, schizophrenia without aggression; HC, healthy controls.

Table A5.9. Group differences in degree using PANSS P7 'Hostility' scores only

	M (95% CI)			ΔAUC, p_{BH}		
Node	SZ+A (*N* = 97)	SZ-A (*N* = 1477)	HC (*N* = 3058)	SZ+A vs SZ-A	SZ+A vs HC	SZ-A vs HC
FPOL	2.4 (0.0, 9.5)	0.0 (0.0, 0.5)	1.0 (0.0, 2.1)	0.370, .506	0.225, .783	−0.145, .508
POP	6.1 (3.6, 11.8)	2.6 (1.1, 6.3)	3.9 (2.4, 6.4)	0.515, .583	0.311, .783	−0.204, .790
PTRI	5.9 (3.0, 11.6)	3.8 (2.2, 5.6)	5.1 (3.3, 6.1)	0.320, .583	0.116, .838	−0.204, .650
PORB	5.9 (2.2, 9.6)	4.2 (2.8, 5.7)	4.7 (3.4, 7.2)	0.261, .717	0.190, .821	−0.071, .914
MOFC	11.5 (4.9, 15.5)	12.0 (8.4, 15.2)	9.0 (6.5, 11.2)	−0.080, .937	0.376, .783	0.455, .570
LOFC	10.1 (6.6, 15.0)	18.4 (13.9, 21.1)	18.2 (13.4, 20.4)	−1.264, .506	−1.249, .783	0.015, .997
CMFG	8.8 (3.8, 13.3)	8.4 (5.9, 10.5)	8.2 (6.8, 10.0)	0.068, .937	0.114, .847	0.046, .968
RMFG	12.2 (5.2, 15.0)	18.0 (13.1, 20.9)	14.9 (10.5, 17.8)	−0.870, .583	−0.395, .802	0.474, .650
SFG	16.7 (9.4, 18.4)	19.5 (14.7, 23.1)	17.9 (13.6, 21.3)	−0.409, .583	−0.188, .826	0.221, .908
PARL	5.1 (1.5, 10.9)	8.5 (5.6, 10.2)	8.1 (5.4, 10.4)	−0.514, .583	−0.435, .783	0.079, .963
PREG	5.6 (2.0, 12.9)	11.8 (7.6, 15.7)	9.3 (6.9, 12.4)	−0.931, .583	−0.556, .783	0.375, .650
CACC	3.8 (1.0, 6.5)	2.5 (2.0, 3.6)	3.1 (2.1, 4.4)	0.195, .611	0.105, .821	−0.090, .650
RACC	4.4 (2.6, 10.0)	11.1 (8.4, 13.2)	8.5 (6.4, 11.2)	−1.011, .506	−0.610, .783	0.401, .596
PCC	7.4 (2.6, 13.5)	12.1 (9.1, 14.9)	10.8 (7.9, 14.8)	−0.740, .583	−0.529, .783	0.211, .893
ISTH	6.2 (1.4, 11.3)	4.8 (2.2, 8.3)	3.6 (2.6, 6.6)	0.220, .879	0.406, .783	0.186, .790
INS	19.5 (9.9, 21.1)	12.2 (8.7, 15.9)	7.9 (6.2, 10.7)	1.113, .506	1.744, .115	0.630, .339
TPOL	3.8 (0.6, 9.7)	0.0 (0.0, 0.7)	1.0 (0.9, 1.9)	0.554, .506	0.404, .783	−0.150, .085
ENTH	2.6 (0.8, 6.4)	0.2 (0.0, 1.2)	2.0 (1.9, 2.6)	0.360, .506	0.085, .821	**−0.275, .008**
PHG	5.2 (2.1, 10.2)	1.2 (0.4, 2.6)	3.4 (2.9, 5.4)	0.595, .506	0.260, .783	**−0.335, .024**
TVTG	4.9 (2.5, 10.5)	5.2 (3.2, 8.6)	8.2 (5.6, 9.8)	−0.054, .937	−0.494, .783	−0.440, .450
ITG	8.5 (3.3, 13.4)	5.6 (3.5, 8.4)	8.1 (5.4, 11.1)	0.450, .583	0.065, .921	−0.385, .596
MTG	4.0 (2.2, 9.1)	10.0 (7.2, 12.8)	6.9 (5.4, 9.1)	−0.905, .506	−0.440, .783	0.466, .339
STG	8.2 (2.8, 15.9)	12.6 (9.2, 15.1)	9.8 (7.8, 12.4)	−0.665, .583	−0.234, .821	0.431, .508
BANKS	5.6 (3.0, 9.6)	3.2 (3.0, 4.8)	3.6 (3.0, 4.7)	0.349, .583	0.294, .783	−0.055, .893
FUSI	13.3 (5.6, 18.3)	12.2 (8.1, 15.7)	10.7 (6.8, 14.6)	0.180, .937	0.399, .783	0.219, .908

POSG	11.6 (5.9, 16.8)	8.0 (5.1, 11.9)	8.2 (5.9, 11.2)	0.577, .583	0.527, .783	−0.050, .989
PREC	6.4 (2.8, 11.4)	18.6 (12.5, 21.6)	18.9 (15.2, 21.0)	−1.861, .506	−1.901, .745	−0.040, .996
SMG	11.3 (5.3, 15.2)	5.3 (2.2, 9.1)	8.6 (6.2, 10.6)	0.901, .506	0.404, .783	−0.497, .339
IPL	4.8 (2.0, 10.1)	10.5 (7.7, 13.2)	9.2 (7.0, 11.7)	−0.845, .506	−0.659, .783	0.186, .801
SPL	5.3 (2.7, 9.8)	6.1 (2.9, 10.7)	6.8 (4.2, 9.8)	−0.114, .937	−0.206, .821	−0.092, .963
LING	3.6 (2.0, 11.6)	3.3 (2.9, 4.1)	4.4 (4.0, 5.8)	0.050, .937	−0.105, .821	−0.155, .210
CUN	3.3 (2.0, 7.2)	4.1 (3.0, 5.9)	5.1 (4.1, 6.0)	−0.115, .879	−0.265, .783	−0.151, .650
PERI	2.7 (2.0, 6.8)	2.1 (2.0, 2.7)	3.4 (2.9, 3.9)	0.095, .583	−0.115, .783	**−0.210, .005**
LOC	3.0 (1.1, 8.1)	6.2 (4.3, 9.0)	6.1 (4.4, 8.8)	−0.486, .583	−0.467, .783	0.019, .996
HIPPO	10.6 (4.5, 14.2)	2.8 (2.1, 5.1)	4.4 (3.4, 5.5)	1.195, .506	0.930, .745	−0.265, .339
AMYG	6.9 (2.0, 12.4)	3.1 (2.2, 5.6)	4.2 (3.2, 6.2)	0.570, .583	0.399, .783	−0.171, .650
CAUD	2.4 (0.0, 5.2)	3.2 (2.2, 3.9)	3.6 (2.8, 4.4)	−0.124, .855	−0.190, .783	−0.066, .674
PUT	7.8 (3.0, 12.3)	5.0 (3.6, 6.0)	5.8 (4.9, 6.4)	0.410, .583	0.290, .783	−0.120, .650
NACC	9.9 (3.8, 14.3)	1.8 (1.0, 2.3)	4.4 (3.3, 5.2)	1.224, .246	0.829, .745	**−0.395, .005**
PALL	3.1 (1.3, 7.5)	3.0 (2.4, 3.2)	2.0 (1.6, 2.0)	0.005, .937	0.155, .783	**0.150, .004**
THAL	6.7 (3.1, 12.0)	3.9 (2.5, 6.7)	3.9 (2.4, 5.6)	0.410, .583	0.410, .783	−0.001, .997

Means were calculated across a range of density thresholds (K = 0.10–0.25, ΔK = 0.01) and are reported with 95% bootstrap confidence intervals (CIs; 5000 resamples). Group differences in area under the curve (ΔAUC) were assessed using permutation testing (10 000 permutations). Bold values indicate statistically significant group differences after Benjamini–Hochberg correction for multiple comparisons ($p_{BH} < .05$). Abbreviations: SZ+A, schizophrenia with aggression; SZ−A, schizophrenia without aggression; HC, healthy controls; FPOL, frontal pole; POP, pars opercularis; PTRI, pars triangularis; PORB, pars orbitalis; MOFC, medial orbitofrontal cortex; LOFC, lateral orbitofrontal cortex; CMFG, caudal middle frontal gyrus; RMFG, rostral middle frontal gyrus; SFG, superior frontal gyrus; PARL, paracentral lobule; PREG, precentral gyrus; CACC, caudal anterior cingulate cortex; RACC, rostral anterior cingulate cortex; PCC, posterior cingulate cortex; ISTH, isthmus of the cingulate cortex; INS, insular cortex; TPOL, temporal pole; ENTH, entorhinal cortex; PHG, parahippocampal gyrus; TVTG, transverse temporal gyrus; ITG, inferior temporal gyrus; MTG, middle temporal gyrus; STG, superior temporal gyrus; BANKS, banks of the superior temporal sulcus; FUSI, fusiform gyrus; POSG, postcentral gyrus; PREC, precuneus; SMG, supramarginal gyrus; IPL, inferior parietal lobule; SPL, superior parietal lobule; LING, lingual gyrus; CUN, cuneus; PERI, pericalcarine cortex; LOC, lateral occipital cortex; HIPPO, hippocampus; AMYG, amygdala; CAUD, caudate nucleus; PUT, putamen; NACC, nucleus accumbens; PALL, globus pallidus; THAL, thalamus.

Table A5.10. Group differences in betweenness centrality using PANSS P7 'Hostility' scores only

	M (95% CI)			ΔAUC, p_{BH}		
Node	SZ+A (*N* = 97)	SZ-A (*N* = 1477)	HC (*N* = 3058)	SZ+A vs SZ-A	SZ+A vs HC	SZ-A vs HC
FPOL	0.000 (0.000, 0.039)	0.000 (0.000, 0.000)	0.000 (0.000, 0.000)	0.00001, .523	0.00001, .910	0.00000, 1.000
POP	0.008 (0.001, 0.064)	0.001 (0.000, 0.011)	0.001 (0.000, 0.007)	0.00098, .815	0.00101, .910	0.00003, 1.000
PTRI	0.003 (0.000, 0.055)	0.023 (0.001, 0.038)	0.004 (0.001, 0.022)	−0.00313, .732	−0.00017, .955	0.00296, .414
PORB	0.001 (0.000, 0.042)	0.031 (0.001, 0.055)	0.002 (0.000, 0.027)	−0.00436, .523	−0.00010, .965	0.00427, .278
MOFC	0.031 (0.004, 0.113)	0.009 (0.002, 0.038)	0.018 (0.002, 0.043)	0.00329, .815	0.00191, .910	−0.00138, .797
LOFC	0.026 (0.016, 0.135)	0.065 (0.029, 0.160)	0.117 (0.037, 0.192)	−0.00575, .815	−0.01355, .820	−0.00781, .757
CMFG	0.030 (0.002, 0.087)	0.001 (0.000, 0.008)	0.016 (0.002, 0.028)	0.00435, .523	0.00230, .820	−0.00205, .347
RMFG	0.051 (0.004, 0.106)	0.079 (0.025, 0.161)	0.069 (0.022, 0.131)	−0.00414, .865	−0.00251, .910	0.00163, 1.000
SFG	0.095 (0.032, 0.159)	0.106 (0.019, 0.255)	0.081 (0.030, 0.210)	−0.00059, .982	0.00266, .910	0.00326, 1.000
PARL	0.001 (0.000, 0.070)	0.001 (0.000, 0.005)	0.004 (0.001, 0.012)	0.00001, .993	−0.00042, .910	−0.00043, .531
PREG	0.026 (0.000, 0.093)	0.007 (0.002, 0.111)	0.011 (0.004, 0.068)	0.00298, .815	0.00259, .910	−0.00039, 1.000
CACC	0.001 (0.000, 0.020)	0.000 (0.000, 0.000)	0.000 (0.000, 0.000)	0.00018, .815	0.00018, .910	0.00000, 1.000
RACC	0.004 (0.002, 0.075)	0.014 (0.008, 0.028)	0.011 (0.005, 0.040)	−0.00163, .815	−0.00109, .910	0.00054, .985
PCC	0.020 (0.002, 0.091)	0.018 (0.010, 0.054)	0.036 (0.012, 0.119)	0.00023, .982	−0.00254, .910	−0.00277, .754
ISTH	0.044 (0.000, 0.126)	0.007 (0.000, 0.034)	0.022 (0.007, 0.056)	0.00569, .523	0.00371, .820	−0.00198, .575
INS	0.207 (0.052, 0.258)	0.016 (0.005, 0.157)	0.005 (0.002, 0.052)	0.02849, .295	0.03022, .102	0.00173, .586
TPOL	0.008 (0.000, 0.079)	0.000 (0.000, 0.002)	0.000 (0.000, 0.013)	0.00120, .523	0.00120, .910	0.00000, 1.000
ENTH	0.004 (0.000, 0.038)	0.000 (0.000, 0.018)	0.049 (0.018, 0.050)	0.00042, .815	−0.00698, .820	**−0.00740, .004**
PHG	0.026 (0.001, 0.077)	0.009 (0.000, 0.091)	0.297 (0.131, 0.346)	0.00240, .815	−0.04126, .910	−0.04366, .078
TVTG	0.005 (0.001, 0.049)	0.000 (0.000, 0.010)	0.008 (0.002, 0.026)	0.00063, .815	−0.00054, .910	−0.00117, .491
ITG	0.041 (0.003, 0.111)	0.000 (0.000, 0.017)	0.086 (0.006, 0.173)	0.00625, .523	−0.00638, .910	−0.01263, .262
MTG	0.004 (0.000, 0.060)	0.012 (0.006, 0.062)	0.012 (0.004, 0.050)	−0.00128, .865	−0.00122, .910	0.00006, 1.000
STG	0.016 (0.001, 0.139)	0.024 (0.014, 0.063)	0.037 (0.017, 0.088)	−0.00135, .886	−0.00319, .910	−0.00184, .811
BANKS	0.017 (0.004, 0.065)	0.000 (0.000, 0.000)	0.001 (0.000, 0.002)	0.00222, .523	0.00213, .820	−0.00009, .262
FUSI	0.179 (0.025, 0.194)	0.076 (0.029, 0.197)	0.322 (0.157, 0.382)	0.01603, .523	−0.02193, .965	−0.03796, .262

POSG	0.064 (0.010, 0.153)	0.004 (0.001, 0.019)	0.013 (0.003, 0.035)	0.00928, .523	0.00791, .820	−0.00137, .576
PREC	0.006 (0.001, 0.064)	0.097 (0.032, 0.221)	0.255 (0.128, 0.336)	−0.01398, .523	−0.03659, .820	−0.02261, .262
SMG	0.055 (0.005, 0.113)	0.002 (0.000, 0.017)	0.011 (0.004, 0.027)	0.00790, .478	0.00656, .820	−0.00134, .262
IPL	0.008 (0.000, 0.061)	0.017 (0.008, 0.052)	0.029 (0.013, 0.111)	−0.00127, .865	−0.00311, .910	−0.00183, .785
SPL	0.008 (0.001, 0.061)	0.006 (0.000, 0.049)	0.019 (0.003, 0.045)	0.00031, .982	−0.00122, .910	−0.00153, .725
LING	0.016 (0.000, 0.108)	0.011 (0.002, 0.023)	0.010 (0.002, 0.056)	0.00084, .886	0.00133, .910	0.00049, .860
CUN	0.014 (0.000, 0.078)	0.027 (0.011, 0.050)	0.020 (0.005, 0.039)	−0.00193, .865	−0.00053, .934	0.00140, .725
PERI	0.000 (0.000, 0.039)	0.000 (0.000, 0.000)	0.000 (0.000, 0.001)	0.00006, .815	0.00003, .910	−0.00003, .262
LOC	0.037 (0.000, 0.090)	0.066 (0.029, 0.116)	0.051 (0.019, 0.100)	−0.00404, .875	−0.00247, .910	0.00157, .977
HIPPO	0.056 (0.006, 0.129)	0.029 (0.000, 0.126)	0.173 (0.054, 0.228)	0.00430, .815	−0.01783, .910	−0.02213, .350
AMYG	0.017 (0.000, 0.095)	0.015 (0.001, 0.128)	0.104 (0.015, 0.176)	0.00086, .904	−0.01203, .910	−0.01290, .540
CAUD	0.002 (0.000, 0.023)	0.001 (0.000, 0.017)	0.001 (0.001, 0.014)	0.00011, .982	0.00009, .955	−0.00003, 1.000
PUT	0.057 (0.008, 0.110)	0.023 (0.010, 0.087)	0.074 (0.042, 0.092)	0.00564, .732	−0.00240, .910	−0.00804, .346
NACC	0.070 (0.007, 0.129)	0.000 (0.000, 0.002)	0.013 (0.003, 0.061)	0.01047, .478	0.00855, .820	−0.00192, .540
PALL	0.007 (0.000, 0.043)	0.002 (0.000, 0.034)	0.000 (0.000, 0.000)	0.00063, .815	0.00089, .820	**0.00026, .018**
THAL	0.039 (0.003, 0.100)	0.010 (0.005, 0.149)	0.019 (0.002, 0.115)	0.00428, .815	0.00277, .910	−0.00151, 1.000

Means were calculated across a range of density thresholds (K = 0.10–0.25, ΔK = 0.01) and are reported with 95% bootstrap confidence intervals (CIs; 5000 resamples). Group differences in area under the curve (ΔAUC) were assessed using permutation testing (10 000 permutations). Bold values indicate statistically significant group differences after Benjamini–Hochberg correction for multiple comparisons ($p_{BH} < .05$). Abbreviations: SZ+A, schizophrenia with aggression; SZ−A, schizophrenia without aggression; HC, healthy controls; FPOL, frontal pole; POP, pars opercularis; PTRI, pars triangularis; PORB, pars orbitalis; MOFC, medial orbitofrontal cortex; LOFC, lateral orbitofrontal cortex; CMFG, caudal middle frontal gyrus; RMFG, rostral middle frontal gyrus; SFG, superior frontal gyrus; PARL, paracentral lobule; PREG, precentral gyrus; CACC, caudal anterior cingulate cortex; RACC, rostral anterior cingulate cortex; PCC, posterior cingulate cortex; ISTH, isthmus of the cingulate cortex; INS, insular cortex; TPOL, temporal pole; ENTH, entorhinal cortex; PHG, parahippocampal gyrus; TVTG, transverse temporal gyrus; ITG, inferior temporal gyrus; MTG, middle temporal gyrus; STG, superior temporal gyrus; BANKS, banks of the superior temporal sulcus; FUSI, fusiform gyrus; POSG, postcentral gyrus; PREC, precuneus; SMG, supramarginal gyrus; IPL, inferior parietal lobule; SPL, superior parietal lobule; LING, lingual gyrus; CUN, cuneus; PERI, pericalcarine cortex; LOC, lateral occipital cortex; HIPPO, hippocampus; AMYG, amygdala; CAUD, caudate nucleus; PUT, putamen; NACC, nucleus accumbens; PALL, globus pallidus; THAL, thalamus.

Table A5.11. Group differences in harmonic centrality using PANSS P7 'Hostility' scores only

	M (95% CI)			ΔAUC, p_{BH}		
Node	SZ+A (*N* = 97)	SZ-A (*N* = 1477)	HC (*N* = 3058)	SZ+A vs SZ-A	SZ+A vs HC	SZ-A vs HC
FPOL	0.405 (0.000, 0.561)	0.000 (0.000, 0.184)	0.204 (0.000, 0.377)	0.062, .366	0.032, .824	−0.030, .365
POP	0.498 (0.427, 0.592)	0.302 (0.259, 0.449)	0.412 (0.359, 0.472)	0.030, .404	0.013, .824	−0.017, .133
PTRI	0.505 (0.415, 0.592)	0.360 (0.314, 0.447)	0.441 (0.393, 0.469)	0.022, .366	0.010, .824	−0.012, .088
PORB	0.509 (0.391, 0.563)	0.401 (0.381, 0.452)	0.445 (0.423, 0.496)	0.016, .404	0.010, .842	−0.007, .175
MOFC	0.580 (0.460, 0.656)	0.514 (0.485, 0.585)	0.520 (0.480, 0.562)	0.010, .545	0.009, .824	−0.001, .890
LOFC	0.577 (0.508, 0.650)	0.604 (0.563, 0.678)	0.655 (0.590, 0.696)	−0.004, .849	−0.012, .816	−0.008, .333
CMFG	0.568 (0.450, 0.625)	0.457 (0.441, 0.517)	0.495 (0.479, 0.538)	0.017, .366	0.011, .816	−0.006, .218
RMFG	0.615 (0.467, 0.648)	0.603 (0.555, 0.674)	0.602 (0.539, 0.654)	0.002, .882	0.002, .899	0.000, .996
SFG	0.679 (0.560, 0.696)	0.620 (0.570, 0.715)	0.648 (0.592, 0.711)	0.009, .545	0.005, .845	−0.004, .754
PARL	0.500 (0.382, 0.595)	0.458 (0.431, 0.517)	0.508 (0.465, 0.547)	0.006, .729	−0.001, .899	−0.008, .251
PREG	0.488 (0.380, 0.613)	0.511 (0.470, 0.603)	0.527 (0.492, 0.580)	−0.003, .898	−0.006, .845	−0.002, .810
CACC	0.438 (0.293, 0.503)	0.350 (0.335, 0.408)	0.406 (0.370, 0.440)	0.014, .534	0.005, .845	−0.008, .080
RACC	0.455 (0.386, 0.573)	0.501 (0.480, 0.562)	0.508 (0.475, 0.565)	−0.007, .729	−0.008, .842	−0.001, .890
PCC	0.543 (0.367, 0.628)	0.516 (0.487, 0.582)	0.553 (0.505, 0.622)	0.004, .849	−0.002, .899	−0.006, .503
ISTH	0.516 (0.264, 0.598)	0.412 (0.377, 0.493)	0.448 (0.425, 0.498)	0.016, .575	0.011, .842	−0.005, .444
INS	0.726 (0.569, 0.737)	0.520 (0.484, 0.613)	0.505 (0.482, 0.560)	0.031, .119	0.033, .529	0.002, .810
TPOL	0.437 (0.138, 0.577)	0.000 (0.000, 0.137)	0.245 (0.226, 0.352)	0.066, .366	0.029, .816	**−0.037, .025**
ENTH	0.391 (0.165, 0.507)	0.055 (0.000, 0.248)	0.320 (0.300, 0.377)	0.052, .366	0.011, .845	**−0.041, .025**
PHG	0.480 (0.348, 0.576)	0.305 (0.093, 0.392)	0.434 (0.405, 0.481)	0.025, .445	0.007, .845	**−0.018, .031**
TVTG	0.491 (0.402, 0.582)	0.419 (0.390, 0.498)	0.517 (0.470, 0.547)	0.011, .729	−0.004, .847	**−0.015, .031**
ITG	0.552 (0.412, 0.629)	0.418 (0.388, 0.498)	0.529 (0.471, 0.589)	0.021, .366	0.003, .896	**−0.017, .031**
MTG	0.446 (0.363, 0.553)	0.487 (0.458, 0.556)	0.493 (0.457, 0.541)	−0.006, .849	−0.007, .845	−0.001, .890
STG	0.552 (0.407, 0.665)	0.523 (0.497, 0.590)	0.538 (0.506, 0.585)	0.004, .849	0.002, .899	−0.002, .766
BANKS	0.487 (0.396, 0.562)	0.354 (0.355, 0.427)	0.408 (0.390, 0.448)	0.020, .381	0.012, .816	−0.008, .098
FUSI	0.647 (0.482, 0.696)	0.528 (0.481, 0.614)	0.574 (0.516, 0.643)	0.018, .366	0.011, .824	−0.007, .503

POSG	0.595 (0.487, 0.675)	0.456 (0.427, 0.539)	0.520 (0.478, 0.567)	0.022, .366	0.012, .816	−0.010, .175
PREC	0.526 (0.392, 0.595)	0.611 (0.547, 0.691)	0.676 (0.632, 0.716)	−0.013, .418	−0.023, .816	−0.010, .161
SMG	0.599 (0.476, 0.650)	0.395 (0.278, 0.496)	0.520 (0.484, 0.557)	0.030, .366	0.012, .816	**−0.018, .008**
IPL	0.473 (0.352, 0.575)	0.499 (0.471, 0.570)	0.538 (0.507, 0.585)	−0.003, .871	−0.010, .824	−0.006, .303
SPL	0.505 (0.393, 0.570)	0.432 (0.391, 0.531)	0.490 (0.447, 0.545)	0.011, .729	0.003, .899	−0.009, .317
LING	0.350 (0.140, 0.592)	0.323 (0.306, 0.375)	0.401 (0.372, 0.467)	0.005, .871	−0.007, .845	−0.**012, .034**
CUN	0.337 (0.137, 0.513)	0.365 (0.321, 0.449)	0.446 (0.391, 0.480)	−0.003, .898	−0.016, .816	−0.012, .115
PERI	0.310 (0.131, 0.506)	0.267 (0.248, 0.328)	0.362 (0.338, 0.392)	0.007, .849	−0.007, .842	**−0.015, .014**
LOC	0.451 (0.205, 0.540)	0.443 (0.407, 0.516)	0.478 (0.417, 0.541)	0.001, .934	−0.005, .891	−0.006, .461
HIPPO	0.609 (0.455, 0.646)	0.174 (0.128, 0.421)	0.377 (0.335, 0.453)	0.067, .119	0.035, .543	**−0.032, .031**
AMYG	0.519 (0.358, 0.614)	0.175 (0.136, 0.423)	0.366 (0.310, 0.468)	0.053, .366	0.023, .816	**−0.030, .049**
CAUD	0.382 (0.000, 0.473)	0.149 (0.131, 0.316)	0.278 (0.254, 0.347)	0.036, .404	0.016, .845	**−0.020, .034**
PUT	0.540 (0.357, 0.607)	0.185 (0.154, 0.378)	0.346 (0.309, 0.406)	0.054, .119	0.029, .816	**−0.025, .031**
NACC	0.587 (0.442, 0.640)	0.129 (0.108, 0.274)	0.323 (0.286, 0.400)	**0.070, .025**	0.040, .543	**−0.030, .014**
PALL	0.392 (0.291, 0.514)	0.149 (0.132, 0.322)	0.258 (0.230, 0.291)	0.037, .366	0.020, .816	**−0.017, .031**
THAL	0.523 (0.416, 0.605)	0.170 (0.143, 0.442)	0.328 (0.290, 0.439)	0.054, .366	0.029, .816	−0.025, .127

Means were calculated across a range of density thresholds (K = 0.10–0.25, ΔK = 0.01) and are reported with 95% bootstrap confidence intervals (CIs; 5000 resamples). Group differences in area under the curve (ΔAUC) were assessed using permutation testing (10 000 permutations). Bold values indicate statistically significant group differences after Benjamini–Hochberg correction for multiple comparisons ($p_{BH} < .05$). Abbreviations: SZ+A, schizophrenia with aggression; SZ−A, schizophrenia without aggression; HC, healthy controls; FPOL, frontal pole; POP, pars opercularis; PTRI, pars triangularis; PORB, pars orbitalis; MOFC, medial orbitofrontal cortex; LOFC, lateral orbitofrontal cortex; CMFG, caudal middle frontal gyrus; RMFG, rostral middle frontal gyrus; SFG, superior frontal gyrus; PARL, paracentral lobule; PREG, precentral gyrus; CACC, caudal anterior cingulate cortex; RACC, rostral anterior cingulate cortex; PCC, posterior cingulate cortex; ISTH, isthmus of the cingulate cortex; INS, insular cortex; TPOL, temporal pole; ENTH, entorhinal cortex; PHG, parahippocampal gyrus; TVTG, transverse temporal gyrus; ITG, inferior temporal gyrus; MTG, middle temporal gyrus; STG, superior temporal gyrus; BANKS, banks of the superior temporal sulcus; FUSI, fusiform gyrus; POSG, postcentral gyrus; PREC, precuneus; SMG, supramarginal gyrus; IPL, inferior parietal lobule; SPL, superior parietal lobule; LING, lingual gyrus; CUN, cuneus; PERI, pericalcarine cortex; LOC, lateral occipital cortex; HIPPO, hippocampus; AMYG, amygdala; CAUD, caudate nucleus; PUT, putamen; NACC, nucleus accumbens; PALL, globus pallidus; THAL, thalamus.

Table A5.12. Group differences in within-module degree using PANSS P7 'Hostility' scores only

	M (95% CI)			ΔAUC, p_{BH}		
Node	SZ+A (*N* = 97)	SZ-A (*N* = 1477)	HC (*N* = 3058)	SZ+A vs SZ-A	SZ+A vs HC	SZ-A vs HC
FPOL	−1.21 (−1.77, 0.48)	0.00 (0.00, 0.00)	−1.76 (−1.87, 0.00)	−0.180, .594	0.083, .920	0.263, .123
POP	−0.41 (−0.99, 1.14)	−0.32 (−1.42, 0.00)	−1.07 (−1.46, −0.50)	−0.015, .998	0.096, .867	0.110, .312
PTRI	−0.34 (−1.12, 1.04)	0.65 (−1.20, 1.15)	−0.78 (−1.24, −0.50)	−0.149, .582	0.067, .867	0.215, .115
PORB	−0.30 (−1.32, 0.67)	−0.32 (−1.22, −0.11)	−0.87 (−1.20, −0.35)	0.003, .998	0.084, .867	0.081, .286
MOFC	1.05 (−0.59, 2.14)	0.37 (−0.19, 1.17)	0.08 (−0.50, 0.62)	0.100, .659	0.143, .867	0.043, .863
LOFC	0.69 (−0.44, 1.91)	1.42 (0.81, 2.28)	2.10 (1.13, 2.68)	−0.114, .622	−0.217, .867	−0.103, .576
CMFG	0.35 (−1.05, 1.41)	−0.20 (−0.66, 0.28)	−0.02 (−0.36, 0.37)	0.083, .646	0.061, .867	−0.023, .869
RMFG	1.23 (−0.62, 1.81)	1.25 (0.61, 2.12)	1.25 (0.38, 1.90)	0.000, .998	0.000, .998	0.000, .999
SFG	2.37 (0.50, 2.80)	1.83 (1.11, 2.49)	2.22 (1.31, 2.85)	0.082, .619	0.024, .997	−0.058, .863
PARL	−0.54 (−1.58, 0.78)	−0.21 (−0.70, 0.23)	−0.11 (−0.67, 0.46)	−0.052, .756	−0.064, .867	−0.012, .952
PREG	−0.60 (−1.37, 1.37)	0.44 (−0.30, 1.17)	0.18 (−0.36, 0.85)	−0.159, .606	−0.118, .867	0.041, .869
CACC	−0.85 (−1.63, 0.13)	−1.39 (−1.61, −1.04)	−1.24 (−1.53, −0.93)	0.084, .594	0.061, .867	−0.023, .836
RACC	−0.68 (−1.16, 0.89)	0.35 (−0.13, 0.83)	−0.02 (−0.49, 0.62)	−0.157, .582	−0.099, .867	0.058, .824
PCC	0.03 (−1.38, 1.49)	0.54 (−0.03, 1.11)	0.56 (−0.13, 1.33)	−0.082, .734	−0.082, .867	0.000, .999
ISTH	−0.66 (−1.49, 1.25)	−1.11 (−1.48, −0.38)	−1.38 (−1.63, −0.52)	0.067, .756	0.112, .867	0.045, .836
INS	3.11 (0.08, 3.24)	0.36 (−0.18, 1.16)	−0.11 (−0.52, 0.48)	**0.413, .012**	**0.483, .029**	0.070, .762
TPOL	−0.90 (−1.60, 0.67)	0.00 (0.00, 0.00)	−0.58 (−0.61, 0.00)	−0.136, .619	−0.049, .996	0.087, .392
ENTH	−1.15 (−1.75, 0.38)	0.00 (0.00, 0.00)	1.15 (0.00, 1.15)	−0.172, .594	−0.346, .867	−0.173, .286
PHG	−0.54 (−1.35, 1.05)	−1.71 (−1.79, 0.00)	−0.58 (−1.04, −0.43)	0.173, .622	0.004, .997	−0.169, .115
TVTG	−0.60 (−1.51, 0.82)	−0.85 (−1.24, −0.13)	−0.11 (−0.71, 0.27)	0.039, .855	−0.073, .867	−0.112, .282
ITG	0.27 (−1.04, 1.53)	−0.79 (−1.16, −0.16)	−0.20 (−0.77, 0.37)	0.161, .582	0.071, .867	−0.090, .448
MTG	−0.81 (−1.35, 0.68)	0.12 (−0.38, 0.66)	−0.36 (−0.71, 0.12)	−0.141, .594	−0.068, .867	0.073, .532
STG	0.20 (−1.32, 2.00)	0.64 (0.02, 1.15)	0.31 (−0.14, 0.88)	−0.070, .756	−0.016, .997	0.053, .810
BANKS	−0.45 (−1.21, 1.02)	−1.24 (−1.39, −0.81)	−1.12 (−1.33, −0.85)	0.118, .594	0.099, .867	−0.020, .845
FUSI	1.25 (−0.68, 2.54)	0.38 (−0.51, 1.02)	−0.01 (−0.77, 0.77)	0.136, .619	0.189, .867	0.053, .869

POSG	0.99 (−0.48, 2.25)	−0.30 (−0.79, 0.51)	−0.10 (−0.62, 0.54)	0.203, .582	0.170, .867	−0.033, .869
PREC	−0.25 (−1.15, 1.10)	1.61 (0.53, 2.14)	1.78 (1.11, 2.51)	−0.281, .582	−0.306, .867	−0.025, .947
SMG	0.94 (−0.62, 1.93)	−0.92 (−1.40, −0.00)	−0.01 (−0.51, 0.48)	0.279, .384	0.140, .867	−0.138, .115
IPL	−0.66 (−1.35, 0.93)	0.22 (−0.31, 0.73)	0.14 (−0.34, 0.68)	−0.129, .594	−0.120, .867	0.009, .952
SPL	−0.47 (−1.22, 0.99)	−0.82 (−1.28, 0.08)	−0.73 (−1.30, −0.01)	0.053, .844	0.041, .996	−0.012, .952
LING	−0.07 (−0.87, 1.42)	0.81 (0.00, 0.87)	0.92 (−0.74, 1.10)	−0.135, .619	−0.151, .867	−0.016, .952
CUN	1.02 (−1.38, 1.23)	0.81 (0.00, 0.91)	−0.20 (−0.63, 0.38)	0.031, .947	0.190, .738	0.159, .060
PERI	0.26 (−1.35, 0.84)	−0.81 (−0.92, 0.00)	0.40 (−1.07, 0.65)	0.160, .582	−0.029, .997	−0.189, .115
LOC	−1.21 (−1.61, 0.47)	−0.81 (−0.94, −0.02)	0.26 (−0.40, 0.62)	−0.056, .756	−0.222, .738	−0.166, .121
HIPPO	0.80 (−0.82, 1.88)	−0.70 (−1.01, 0.43)	−0.31 (−0.80, 0.37)	0.229, .582	0.168, .867	−0.061, .834
AMYG	−0.11 (−1.41, 1.44)	−0.09 (−0.68, 0.23)	−0.18 (−0.45, 0.46)	−0.003, .998	0.013, .997	0.017, .952
CAUD	−1.21 (−1.74, 0.05)	−0.03 (−0.46, 0.70)	−0.13 (−0.48, 0.36)	−0.179, .582	−0.159, .867	0.020, .947
PUT	0.10 (−0.86, 1.67)	1.63 (0.95, 1.90)	1.59 (1.27, 1.84)	−0.231, .594	−0.227, .867	0.005, .980
NACC	0.50 (−1.08, 1.62)	−1.33 (−1.53, −0.85)	0.46 (−0.05, 0.81)	0.273, .384	0.006, .997	**−0.267, .004**
PALL	−1.01 (−1.54, 1.08)	−0.09 (−0.65, 0.40)	−1.45 (−1.73, −1.03)	−0.139, .582	0.068, .878	**0.207, .004**
THAL	−0.13 (−1.14, 1.28)	0.60 (−0.25, 1.03)	0.02 (−0.82, 0.45)	−0.109, .756	−0.030, .997	0.079, .810

Means were calculated across a range of density thresholds (K = 0.10–0.25, ΔK = 0.01) and are reported with 95% bootstrap confidence intervals (CIs; 5000 resamples). Group differences in area under the curve (ΔAUC) were assessed using permutation testing (10 000 permutations). Bold values indicate statistically significant group differences after Benjamini–Hochberg correction for multiple comparisons ($p_{BH} < .05$). Abbreviations: SZ+A, schizophrenia with aggression; SZ−A, schizophrenia without aggression; HC, healthy controls; FPOL, frontal pole; POP, pars opercularis; PTRI, pars triangularis; PORB, pars orbitalis; MOFC, medial orbitofrontal cortex; LOFC, lateral orbitofrontal cortex; CMFG, caudal middle frontal gyrus; RMFG, rostral middle frontal gyrus; SFG, superior frontal gyrus; PARL, paracentral lobule; PREG, precentral gyrus; CACC, caudal anterior cingulate cortex; RACC, rostral anterior cingulate cortex; PCC, posterior cingulate cortex; ISTH, isthmus of the cingulate cortex; INS, insular cortex; TPOL, temporal pole; ENTH, entorhinal cortex; PHG, parahippocampal gyrus; TVTG, transverse temporal gyrus; ITG, inferior temporal gyrus; MTG, middle temporal gyrus; STG, superior temporal gyrus; BANKS, banks of the superior temporal sulcus; FUSI, fusiform gyrus; POSG, postcentral gyrus; PREC, precuneus; SMG, supramarginal gyrus; IPL, inferior parietal lobule; SPL, superior parietal lobule; LING, lingual gyrus; CUN, cuneus; PERI, pericalcarine cortex; LOC, lateral occipital cortex; HIPPO, hippocampus; AMYG, amygdala; CAUD, caudate nucleus; PUT, putamen; NACC, nucleus accumbens; PALL, globus pallidus; THAL, thalamus.

Table A5.13. Group differences in participation coefficient using PANSS P7 'Hostility' scores only

Node	M (95% CI)			ΔAUC, p_{BH}		
	SZ+A (*N* = 97)	SZ-A (*N* = 1477)	HC (*N* = 3058)	SZ+A vs SZ-A	SZ+A vs HC	SZ-A vs HC
FPOL	0.00 (0.00, 0.43)	0.00 (0.00, 0.00)	0.00 (0.00, 0.00)	0.000, 1.000	0.000, 1.000	0.000, 1.000
POP	0.09 (0.00, 0.44)	0.14 (0.00, 0.33)	0.00 (0.00, 0.00)	−0.007, .984	0.014, 1.000	0.020, .127
PTRI	0.00 (0.00, 0.45)	0.30 (0.00, 0.43)	0.00 (0.00, 0.00)	−0.045, .735	0.000, 1.000	0.045, .127
PORB	0.00 (0.00, 0.39)	0.44 (0.00, 0.49)	0.02 (0.00, 0.16)	−0.066, .735	−0.001, 1.000	0.065, .113
MOFC	0.00 (0.00, 0.46)	0.09 (0.00, 0.14)	0.01 (0.00, 0.11)	−0.014, .735	−0.001, 1.000	0.014, .113
LOFC	0.00 (0.00, 0.54)	0.19 (0.00, 0.22)	0.06 (0.00, 0.11)	−0.028, .735	−0.009, 1.000	0.019, .118
CMFG	0.00 (0.00, 0.55)	0.00 (0.00, 0.03)	0.00 (0.00, 0.07)	0.000, 1.000	0.000, 1.000	0.000, 1.000
RMFG	0.00 (0.00, 0.48)	0.23 (0.00, 0.26)	0.09 (0.00, 0.18)	−0.035, .735	−0.013, 1.000	0.022, .175
SFG	0.00 (0.00, 0.54)	0.11 (0.00, 0.20)	0.00 (0.00, 0.10)	−0.016, .735	0.000, 1.000	0.016, .371
PARL	0.00 (0.00, 0.50)	0.00 (0.00, 0.00)	0.00 (0.00, 0.00)	0.000, 1.000	0.000, 1.000	0.000, 1.000
PREG	0.20 (0.00, 0.51)	0.00 (0.00, 0.12)	0.00 (0.00, 0.08)	0.031, .735	0.031, 1.000	0.000, 1.000
CACC	0.00 (0.00, 0.42)	0.00 (0.00, 0.00)	0.00 (0.00, 0.00)	0.000, 1.000	0.000, 1.000	0.000, 1.000
RACC	0.00 (0.00, 0.52)	0.00 (0.00, 0.02)	0.02 (0.00, 0.12)	0.000, 1.000	−0.002, 1.000	−0.002, .565
PCC	0.00 (0.00, 0.53)	0.00 (0.00, 0.07)	0.00 (0.00, 0.10)	0.000, 1.000	0.000, 1.000	0.000, 1.000
ISTH	0.26 (0.00, 0.60)	0.14 (0.00, 0.39)	0.49 (0.00, 0.50)	0.019, 1.000	−0.033, 1.000	−0.051, .371
INS	0.00 (0.00, 0.63)	0.11 (0.00, 0.20)	0.00 (0.00, 0.09)	−0.017, .924	0.000, 1.000	0.017, .113
TPOL	0.00 (0.00, 0.58)	0.00 (0.00, 0.06)	0.00 (0.00, 0.38)	0.000, 1.000	0.000, 1.000	0.000, 1.000
ENTH	0.00 (0.00, 0.50)	0.00 (0.00, 0.22)	0.00 (0.00, 0.50)	0.000, 1.000	0.000, 1.000	0.000, 1.000
PHG	0.03 (0.00, 0.55)	0.10 (0.00, 0.46)	0.66 (0.60, 0.72)	−0.010, 1.000	−0.095, 1.000	**−0.085, .008**
TVTG	0.00 (0.00, 0.55)	0.00 (0.00, 0.09)	0.00 (0.00, 0.02)	0.000, 1.000	0.000, 1.000	0.000, 1.000
ITG	0.00 (0.00, 0.60)	0.00 (0.00, 0.06)	0.05 (0.00, 0.24)	0.000, 1.000	−0.007, 1.000	−0.007, .872
MTG	0.00 (0.00, 0.51)	0.00 (0.00, 0.08)	0.00 (0.00, 0.10)	0.000, 1.000	0.000, 1.000	0.000, 1.000
STG	0.00 (0.00, 0.56)	0.00 (0.00, 0.08)	0.00 (0.00, 0.08)	0.000, 1.000	0.000, 1.000	0.000, 1.000
BANKS	0.00 (0.00, 0.57)	0.00 (0.00, 0.00)	0.00 (0.00, 0.00)	0.000, 1.000	0.000, 1.000	0.000, 1.000
FUSI	0.14 (0.00, 0.63)	0.16 (0.00, 0.37)	0.33 (0.22, 0.45)	−0.002, 1.000	−0.028, 1.000	−0.026, .165

POSG	0.03 (0.00, 0.56)	0.00 (0.00, 0.03)	0.00 (0.00, 0.11)	0.004, .924	0.004, 1.000	0.000, 1.000
PREC	0.00 (0.00, 0.53)	0.13 (0.01, 0.24)	0.26 (0.00, 0.33)	−0.020, .735	−0.039, 1.000	−0.019, .669
SMG	0.00 (0.00, 0.53)	0.04 (0.00, 0.13)	0.00 (0.00, 0.05)	−0.006, .735	0.000, 1.000	0.006, .113
IPL	0.00 (0.00, 0.54)	0.01 (0.00, 0.10)	0.00 (0.00, 0.14)	−0.001, 1.000	0.000, 1.000	0.001, 1.000
SPL	0.00 (0.00, 0.54)	0.11 (0.00, 0.29)	0.32 (0.00, 0.43)	−0.016, .924	−0.049, 1.000	−0.033, .566
LING	0.36 (0.00, 0.57)	0.12 (0.00, 0.48)	0.11 (0.00, 0.52)	0.039, .735	0.040, 1.000	0.002, 1.000
CUN	0.19 (0.00, 0.50)	0.26 (0.00, 0.48)	0.46 (0.00, 0.48)	−0.011, 1.000	−0.042, 1.000	−0.031, .566
PERI	0.21 (0.00, 0.47)	0.00 (0.00, 0.14)	0.00 (0.00, 0.19)	0.032, .735	0.032, 1.000	0.000, 1.000
LOC	0.37 (0.00, 0.57)	0.43 (0.34, 0.49)	0.43 (0.00, 0.51)	−0.008, 1.000	−0.010, 1.000	−0.002, 1.000
HIPPO	0.00 (0.00, 0.59)	0.08 (0.00, 0.41)	0.35 (0.03, 0.48)	−0.010, 1.000	−0.053, 1.000	−0.043, .229
AMYG	0.00 (0.00, 0.55)	0.05 (0.00, 0.43)	0.18 (0.00, 0.42)	−0.005, 1.000	−0.026, 1.000	−0.020, 1.000
CAUD	0.00 (0.00, 0.43)	0.00 (0.00, 0.07)	0.00 (0.00, 0.00)	0.000, 1.000	0.000, 1.000	0.000, 1.000
PUT	0.00 (0.00, 0.57)	0.00 (0.00, 0.32)	0.00 (0.00, 0.11)	0.000, 1.000	0.000, 1.000	0.000, 1.000
NACC	0.06 (0.00, 0.60)	0.00 (0.00, 0.00)	0.00 (0.00, 0.15)	0.009, 1.000	0.009, 1.000	0.000, 1.000
PALL	0.00 (0.00, 0.46)	0.00 (0.00, 0.39)	0.00 (0.00, 0.00)	0.000, 1.000	0.000, 1.000	0.000, 1.000
THAL	0.00 (0.00, 0.57)	0.00 (0.00, 0.47)	0.00 (0.00, 0.26)	0.000, 1.000	0.000, 1.000	0.000, 1.000

Means were calculated across a range of density thresholds (K = 0.10–0.25, ΔK = 0.01) and are reported with 95% bootstrap confidence intervals (CIs; 5000 resamples). Group differences in area under the curve (ΔAUC) were assessed using permutation testing (10 000 permutations). Bold values indicate statistically significant group differences after Benjamini–Hochberg correction for multiple comparisons ($p_{BH} < .05$). Abbreviations: SZ+A, schizophrenia with aggression; SZ−A, schizophrenia without aggression; HC, healthy controls; FPOL, frontal pole; POP, pars opercularis; PTRI, pars triangularis; PORB, pars orbitalis; MOFC, medial orbitofrontal cortex; LOFC, lateral orbitofrontal cortex; CMFG, caudal middle frontal gyrus; RMFG, rostral middle frontal gyrus; SFG, superior frontal gyrus; PARL, paracentral lobule; PREG, precentral gyrus; CACC, caudal anterior cingulate cortex; RACC, rostral anterior cingulate cortex; PCC, posterior cingulate cortex; ISTH, isthmus of the cingulate cortex; INS, insular cortex; TPOL, temporal pole; ENTH, entorhinal cortex; PHG, parahippocampal gyrus; TVTG, transverse temporal gyrus; ITG, inferior temporal gyrus; MTG, middle temporal gyrus; STG, superior temporal gyrus; BANKS, banks of the superior temporal sulcus; FUSI, fusiform gyrus; POSG, postcentral gyrus; PREC, precuneus; SMG, supramarginal gyrus; IPL, inferior parietal lobule; SPL, superior parietal lobule; LING, lingual gyrus; CUN, cuneus; PERI, pericalcarine cortex; LOC, lateral occipital cortex; HIPPO, hippocampus; AMYG, amygdala; CAUD, caudate nucleus; PUT, putamen; NACC, nucleus accumbens; PALL, globus pallidus; THAL, thalamus.

Table A5.14. Group differences in canonical brain network density using PANSS P7 'Hostility' scores only

Metric	M (95% CI)			ΔAUC, p_{BH}		
	SZ+A (*N* = 97)	SZ-A (*N* = 1477)	HC (*N* = 3058)	SZ+A vs SZ-A	SZ+A vs HC	SZ-A vs HC
Salience network						
Network-rest	0.30 (0.16, 0.32)	0.19 (0.14, 0.24)	0.14 (0.12, 0.18)	0.017, .154	**0.024, .035**	0.007, .406
Central executive network						
Within-network	0.34 (0.00, 0.48)	0.52 (0.36, 0.66)	0.29 (0.17, 0.51)	−0.026, .735	0.010, 1.000	0.036, .322
Network-rest	0.18 (0.12, 0.24)	0.25 (0.21, 0.28)	0.24 (0.21, 0.26)	−0.010, .249	−0.009, .673	0.001, .737
Default mode network						
Within-network	0.19 (0.11, 0.27)	0.15 (0.13, 0.19)	0.18 (0.15, 0.20)	0.005, .735	0.001, 1.000	−0.003, .487
Network-rest	0.15 (0.13, 0.18)	0.16 (0.14, 0.17)	0.15 (0.14, 0.16)	−0.001, .776	0.001, .828	0.001, .472
Language network						
Within-network	0.28 (0.10, 0.55)	0.37 (0.30, 0.40)	0.31 (0.30, 0.38)	−0.014, .735	−0.005, 1.000	0.009, .487
Network-rest	0.13 (0.11, 0.20)	0.18 (0.15, 0.21)	0.16 (0.14, 0.18)	−0.007, .360	−0.004, .828	0.003, .472
Reward network						
Within-network	0.38 (0.18, 0.46)	0.31 (0.24, 0.36)	0.39 (0.33, 0.43)	0.009, .735	−0.003, 1.000	−0.012, .322
Network-rest	0.15 (0.12, 0.19)	0.16 (0.14, 0.17)	0.14 (0.12, 0.15)	−0.002, .776	0.001, .828	0.003, .407
Affective control network						
Within-network	0.38 (0.25, 0.42)	0.31 (0.27, 0.36)	0.32 (0.28, 0.34)	0.010, .735	0.009, 1.000	−0.001, 1.000
Network-rest	0.14 (0.12, 0.17)	0.17 (0.16, 0.18)	0.14 (0.14, 0.15)	−0.005, .098	−0.001, .828	**0.004, .007**

Means were calculated across a range of density thresholds (*K* = 0.10–0.25, Δ*K* = 0.01) and are reported with 95% bootstrap confidence intervals (CIs; 5000 resamples). Group differences in area under the curve (ΔAUC) were assessed using permutation testing (10 000 permutations). Bold values indicate statistically significant group differences after Benjamini–Hochberg correction for multiple comparisons (p_{BH} < .05). Abbreviations: SZ+A, schizophrenia with aggression; SZ−A, schizophrenia without aggression; HC, healthy controls.

Table A5.15. Group differences in global network topology using regional volumes residualized for CPZ-equivalent dose.

	M (95% CI)			ΔAUC, p_{BH}		
Metric	SZ+A (*N* = 212)	SZ-A (*N* = 1533)	HC (*N* = 3058)	SZ+A vs SZ-A	SZ+A vs HC	SZ-A vs HC
Transitivity	0.45 (0.39, 0.51)	0.55 (0.50, 0.58)	0.50 (0.46, 0.52)	−0.015, .166	−0.007, .893	**0.008, .037**
Modularity	0.39 (0.31, 0.42)	0.31 (0.28, 0.34)	0.39 (0.36, 0.42)	**0.012, .040**	−0.001, .964	**−0.013, .005**
Efficiency	0.47 (0.44, 0.51)	0.40 (0.38, 0.45)	0.45 (0.44, 0.48)	0.010, .166	0.003, .893	**−0.008, .010**
Small-worldness	1.58 (1.33, 1.83)	1.19 (1.15, 1.37)	1.47 (1.33, 1.65)	**0.058, .040**	0.018, .893	**−0.040, .037**

Means were calculated across a range of density thresholds (*K* = 0.10–0.25, Δ*K* = 0.01) and are reported with 95% bootstrap confidence intervals (CIs; 5000 resamples). Group differences in area under the curve (ΔAUC) were assessed using permutation testing (10 000 permutations). Bold values indicate statistically significant group differences after Benjamini–Hochberg correction for multiple comparisons (p_{BH} < .05). Abbreviations: SZ+A, schizophrenia with aggression; SZ−A, schizophrenia without aggression; HC, healthy controls.

Table A5.16. Group differences in degree using regional volumes residualized for CPZ-equivalent dose.

	M (95% CI)			ΔAUC, p_{BH}		
Node	SZ+A (*N* = 212)	SZ-A (*N* = 1533)	HC (*N* = 3058)	SZ+A vs SZ-A	SZ+A vs HC	SZ-A vs HC
FPOL	1.2 (0.0, 6.1)	0.1 (0.0, 1.2)	1.0 (0.0, 2.1)	0.185, .748	0.045, .882	−0.140, .640
POP	2.1 (1.0, 8.4)	3.4 (1.4, 6.9)	3.9 (2.4, 6.4)	−0.196, .899	−0.280, .860	−0.084, .956
PTRI	4.6 (1.9, 8.3)	5.5 (3.5, 6.9)	5.1 (3.3, 6.1)	−0.135, .899	−0.079, .882	0.055, .956
PORB	5.1 (2.4, 8.1)	4.8 (3.2, 6.1)	4.7 (3.4, 7.2)	0.050, .944	0.076, .882	0.026, .975
MOFC	12.5 (7.3, 16.9)	13.1 (9.5, 15.9)	9.0 (6.5, 11.2)	−0.070, .944	0.537, .860	0.606, .302
LOFC	9.6 (6.5, 13.9)	18.1 (13.8, 20.7)	18.2 (13.4, 20.4)	−1.305, .692	−1.326, .860	−0.020, .985
CMFG	6.1 (2.8, 10.6)	7.6 (5.3, 9.9)	8.2 (6.8, 10.0)	−0.239, .899	−0.304, .860	−0.065, .956
RMFG	12.8 (6.8, 16.2)	18.1 (13.4, 20.9)	14.9 (10.5, 17.8)	−0.801, .748	−0.305, .860	0.495, .649
SFG	15.2 (10.6, 18.0)	18.5 (13.8, 22.4)	17.9 (13.6, 21.3)	−0.497, .748	−0.430, .860	0.067, .975
PARL	5.6 (1.9, 9.9)	9.3 (6.6, 10.6)	8.1 (5.4, 10.4)	−0.566, .748	−0.361, .860	0.205, .854
PREG	7.5 (4.0, 12.7)	10.0 (6.3, 14.2)	9.3 (6.9, 12.4)	−0.376, .899	−0.270, .860	0.105, .956
CACC	4.4 (2.6, 9.2)	2.7 (2.0, 4.0)	3.1 (2.1, 4.4)	0.240, .794	0.180, .860	−0.060, .854
RACC	10.4 (6.7, 13.1)	11.7 (8.5, 13.6)	8.5 (6.4, 11.2)	−0.190, .899	0.305, .860	0.495, .383
PCC	8.2 (2.9, 12.9)	13.1 (9.6, 15.9)	10.8 (7.9, 14.8)	−0.726, .748	−0.379, .860	0.347, .759
ISTH	4.6 (1.0, 10.0)	3.6 (1.6, 6.8)	3.6 (2.6, 6.6)	0.155, .899	0.151, .860	−0.005, .985
INS	17.6 (9.8, 19.9)	12.2 (8.6, 16.0)	7.9 (6.2, 10.7)	0.816, .748	1.461, .076	0.645, .242
TPOL	1.6 (0.0, 5.2)	0.2 (0.0, 1.0)	1.0 (0.9, 1.9)	0.215, .748	0.090, .860	−0.125, .066
ENTH	2.0 (0.1, 4.3)	0.4 (0.0, 1.7)	2.0 (1.9, 2.6)	0.255, .692	0.005, .887	**−0.250, .004**
PHG	4.8 (1.6, 7.2)	1.9 (1.0, 3.3)	3.4 (2.9, 5.4)	0.450, .692	0.215, .860	−0.235, .069
TVTG	6.6 (4.4, 10.9)	5.0 (2.8, 8.7)	8.2 (5.6, 9.8)	0.245, .899	−0.240, .860	−0.485, .283
ITG	10.8 (5.8, 15.1)	5.1 (2.9, 8.1)	8.1 (5.4, 11.1)	0.865, .692	0.399, .860	−0.466, .380
MTG	8.1 (5.1, 13.0)	10.9 (7.8, 13.9)	6.9 (5.4, 9.1)	−0.430, .854	0.170, .860	0.600, .200
STG	14.8 (9.5, 19.2)	12.9 (9.4, 15.5)	9.8 (7.8, 12.4)	0.266, .899	0.741, .860	0.476, .380
BANKS	3.8 (2.0, 6.3)	3.0 (3.0, 4.0)	3.6 (3.0, 4.7)	0.115, .899	0.035, .882	−0.080, .649
FUSI	13.0 (6.6, 17.3)	12.8 (8.9, 16.4)	10.7 (6.8, 14.6)	0.051, .944	0.349, .860	0.299, .854

POSG	12.6 (7.0, 16.9)	8.8 (5.4, 12.9)	8.2 (5.9, 11.2)	0.604, .789	0.671, .860	0.066, .975
PREC	11.2 (5.8, 15.3)	16.6 (10.3, 20.1)	18.9 (15.2, 21.0)	−0.820, .755	−1.167, .860	−0.347, .709
SMG	7.2 (4.5, 11.3)	5.2 (2.6, 8.8)	8.6 (6.2, 10.6)	0.315, .899	−0.202, .860	−0.518, .242
IPL	5.8 (3.2, 10.4)	9.0 (6.0, 12.4)	9.2 (7.0, 11.7)	−0.495, .852	−0.524, .860	−0.029, .975
SPL	6.1 (3.0, 11.0)	5.4 (2.6, 9.9)	6.8 (4.2, 9.8)	0.079, .944	−0.101, .882	−0.180, .917
LING	3.5 (2.3, 7.0)	3.2 (2.8, 4.0)	4.4 (4.0, 5.8)	0.050, .942	−0.125, .860	−0.175, .051
CUN	3.9 (2.8, 7.9)	3.0 (3.0, 4.0)	5.1 (4.1, 6.0)	0.130, .854	−0.185, .860	−0.315, .058
PERI	3.0 (2.0, 4.8)	2.0 (2.0, 2.6)	3.4 (2.9, 3.9)	0.150, .692	−0.065, .860	**−0.215, .004**
LOC	4.4 (1.6, 10.2)	5.4 (3.9, 8.1)	6.1 (4.4, 8.8)	−0.150, .899	−0.251, .860	−0.101, .923
HIPPO	3.8 (2.2, 7.6)	3.6 (2.4, 6.3)	4.4 (3.4, 5.5)	0.030, .944	−0.105, .860	−0.136, .649
AMYG	6.9 (3.1, 10.9)	3.6 (2.6, 6.8)	4.2 (3.2, 6.2)	0.510, .748	0.399, .860	−0.111, .854
CAUD	3.0 (1.1, 4.2)	3.2 (2.4, 4.0)	3.6 (2.8, 4.4)	−0.040, .944	−0.095, .860	−0.055, .807
PUT	7.0 (3.6, 9.3)	5.2 (3.7, 6.1)	5.8 (4.9, 6.4)	0.276, .748	0.185, .860	−0.091, .648
NACC	6.0 (2.4, 10.8)	1.9 (1.2, 2.8)	4.4 (3.3, 5.2)	0.595, .692	0.230, .860	**−0.365, .004**
PALL	4.6 (2.2, 8.4)	2.9 (2.1, 3.2)	2.0 (1.6, 2.0)	0.234, .692	0.379, .076	**0.145, .007**
THAL	5.1 (2.2, 9.2)	4.1 (2.3, 6.2)	3.9 (2.4, 5.6)	0.151, .899	0.170, .860	0.020, .975

Means were calculated across a range of density thresholds (K = 0.10–0.25, ΔK = 0.01) and are reported with 95% bootstrap confidence intervals (CIs; 5000 resamples). Group differences in area under the curve (ΔAUC) were assessed using permutation testing (10 000 permutations). Bold values indicate statistically significant group differences after Benjamini–Hochberg correction for multiple comparisons ($p_{BH} < .05$). Abbreviations: SZ+A, schizophrenia with aggression; SZ−A, schizophrenia without aggression; HC, healthy controls; FPOL, frontal pole; POP, pars opercularis; PTRI, pars triangularis; PORB, pars orbitalis; MOFC, medial orbitofrontal cortex; LOFC, lateral orbitofrontal cortex; CMFG, caudal middle frontal gyrus; RMFG, rostral middle frontal gyrus; SFG, superior frontal gyrus; PARL, paracentral lobule; PREG, precentral gyrus; CACC, caudal anterior cingulate cortex; RACC, rostral anterior cingulate cortex; PCC, posterior cingulate cortex; ISTH, isthmus of the cingulate cortex; INS, insular cortex; TPOL, temporal pole; ENTH, entorhinal cortex; PHG, parahippocampal gyrus; TVTG, transverse temporal gyrus; ITG, inferior temporal gyrus; MTG, middle temporal gyrus; STG, superior temporal gyrus; BANKS, banks of the superior temporal sulcus; FUSI, fusiform gyrus; POSG, postcentral gyrus; PREC, precuneus; SMG, supramarginal gyrus; IPL, inferior parietal lobule; SPL, superior parietal lobule; LING, lingual gyrus; CUN, cuneus; PERI, pericalcarine cortex; LOC, lateral occipital cortex; HIPPO, hippocampus; AMYG, amygdala; CAUD, caudate nucleus; PUT, putamen; NACC, nucleus accumbens; PALL, globus pallidus; THAL, thalamus.

Table A5.17. Group differences in betweenness centrality using regional volumes residualized for CPZ-equivalent dose.

	M (95% CI)			ΔAUC, p_{BH}		
Node	SZ+A (*N* = 212)	SZ-A (*N* = 1533)	HC (*N* = 3058)	SZ+A vs SZ-A	SZ+A vs HC	SZ-A vs HC
FPOL	0.000 (0.000, 0.007)	0.000 (0.000, 0.000)	0.000 (0.000, 0.000)	0.00000, 1.000	0.00000, 1.000	0.00000, 1.000
POP	0.000 (0.000, 0.031)	0.000 (0.000, 0.006)	0.001 (0.000, 0.007)	0.00000, 1.000	−0.00007, .995	−0.00007, .896
PTRI	0.025 (0.001, 0.049)	0.015 (0.001, 0.035)	0.004 (0.001, 0.022)	0.00151, 1.000	0.00314, .835	0.00163, .885
PORB	0.003 (0.000, 0.039)	0.003 (0.000, 0.032)	0.002 (0.000, 0.027)	−0.00002, 1.000	0.00021, .995	0.00022, 1.000
MOFC	0.035 (0.009, 0.135)	0.017 (0.005, 0.055)	0.018 (0.002, 0.043)	0.00296, 1.000	0.00260, .893	−0.00035, 1.000
LOFC	0.017 (0.007, 0.106)	0.070 (0.028, 0.154)	0.117 (0.037, 0.192)	−0.00816, 1.000	−0.01520, .843	−0.00704, .885
CMFG	0.003 (0.000, 0.050)	0.001 (0.000, 0.007)	0.016 (0.002, 0.028)	0.00032, 1.000	−0.00179, .893	−0.00211, .266
RMFG	0.058 (0.014, 0.125)	0.091 (0.031, 0.185)	0.069 (0.022, 0.131)	−0.00507, 1.000	−0.00160, .995	0.00347, .896
SFG	0.070 (0.024, 0.135)	0.111 (0.020, 0.259)	0.081 (0.030, 0.210)	−0.00619, 1.000	−0.00127, .995	0.00492, .896
PARL	0.002 (0.000, 0.023)	0.003 (0.001, 0.008)	0.004 (0.001, 0.012)	−0.00008, 1.000	−0.00028, .995	−0.00020, .896
PREG	0.005 (0.001, 0.069)	0.005 (0.001, 0.033)	0.011 (0.004, 0.068)	−0.00001, 1.000	−0.00075, .995	−0.00074, .885
CACC	0.002 (0.000, 0.035)	0.000 (0.000, 0.000)	0.000 (0.000, 0.000)	0.00032, .984	0.00032, .893	0.00000, 1.000
RACC	0.050 (0.013, 0.120)	0.013 (0.007, 0.029)	0.011 (0.005, 0.040)	0.00542, .707	0.00578, .843	0.00036, 1.000
PCC	0.014 (0.002, 0.089)	0.022 (0.012, 0.065)	0.036 (0.012, 0.119)	−0.00120, 1.000	−0.00337, .979	−0.00217, .885
ISTH	0.034 (0.000, 0.125)	0.008 (0.000, 0.036)	0.022 (0.007, 0.056)	0.00413, .984	0.00222, .930	−0.00191, .718
INS	0.179 (0.033, 0.244)	0.028 (0.006, 0.157)	0.005 (0.002, 0.052)	0.02357, .707	0.02641, .369	0.00284, .588
TPOL	0.007 (0.000, 0.054)	0.000 (0.000, 0.006)	0.000 (0.000, 0.013)	0.00106, .984	0.00106, .893	0.00000, 1.000
ENTH	0.004 (0.000, 0.035)	0.009 (0.000, 0.030)	0.049 (0.018, 0.050)	−0.00058, 1.000	−0.00676, .843	−0.00618, .057
PHG	0.017 (0.000, 0.097)	0.071 (0.000, 0.160)	0.297 (0.131, 0.346)	−0.00842, 1.000	−0.04230, .835	−0.03388, .150
TVTG	0.002 (0.001, 0.031)	0.001 (0.000, 0.008)	0.008 (0.002, 0.026)	0.00022, 1.000	−0.00087, .930	−0.00109, .494
ITG	0.067 (0.011, 0.160)	0.000 (0.000, 0.011)	0.086 (0.006, 0.173)	0.01011, .707	−0.00250, .995	−0.01261, .266
MTG	0.026 (0.007, 0.107)	0.020 (0.009, 0.063)	0.012 (0.004, 0.050)	0.00092, 1.000	0.00206, .930	0.00114, .885
STG	0.062 (0.017, 0.197)	0.036 (0.021, 0.087)	0.037 (0.017, 0.088)	0.00365, 1.000	0.00347, .893	−0.00018, 1.000
BANKS	0.001 (0.000, 0.018)	0.000 (0.000, 0.001)	0.001 (0.000, 0.002)	0.00011, 1.000	0.00005, .995	−0.00006, .425
FUSI	0.175 (0.052, 0.264)	0.212 (0.066, 0.304)	0.322 (0.157, 0.382)	−0.00546, 1.000	−0.02216, .995	−0.01671, .688

POSG	0.051 (0.009, 0.144)	0.007 (0.002, 0.025)	0.013 (0.003, 0.035)	0.00690, .707	0.00589, .835	−0.00101, .885
PREC	0.067 (0.010, 0.163)	0.083 (0.031, 0.187)	0.255 (0.128, 0.336)	−0.00256, 1.000	−0.02752, .893	−0.02496, .266
SMG	0.007 (0.001, 0.045)	0.002 (0.000, 0.010)	0.011 (0.004, 0.027)	0.00075, 1.000	−0.00061, .995	−0.00137, .266
IPL	0.008 (0.001, 0.056)	0.016 (0.007, 0.045)	0.029 (0.013, 0.111)	−0.00118, 1.000	−0.00309, .893	−0.00192, .885
SPL	0.012 (0.000, 0.077)	0.002 (0.000, 0.034)	0.019 (0.003, 0.045)	0.00152, 1.000	−0.00055, .995	−0.00207, .722
LING	0.004 (0.000, 0.081)	0.023 (0.008, 0.031)	0.010 (0.002, 0.056)	−0.00282, 1.000	−0.00061, .995	0.00222, .328
CUN	0.008 (0.001, 0.096)	0.019 (0.008, 0.038)	0.020 (0.005, 0.039)	−0.00164, 1.000	−0.00152, .930	0.00012, 1.000
PERI	0.000 (0.000, 0.018)	0.000 (0.000, 0.000)	0.000 (0.000, 0.001)	0.00002, 1.000	−0.00001, .995	−0.00003, .150
LOC	0.054 (0.001, 0.134)	0.115 (0.070, 0.137)	0.051 (0.019, 0.100)	−0.00909, 1.000	0.00016, 1.000	0.00925, .150
HIPPO	0.003 (0.001, 0.064)	0.089 (0.002, 0.179)	0.173 (0.054, 0.228)	−0.01349, 1.000	−0.02585, .467	−0.01235, .718
AMYG	0.027 (0.003, 0.129)	0.056 (0.006, 0.164)	0.104 (0.015, 0.176)	−0.00470, 1.000	−0.01066, .930	−0.00596, .885
CAUD	0.001 (0.000, 0.015)	0.001 (0.000, 0.011)	0.001 (0.001, 0.014)	−0.00004, 1.000	−0.00004, .995	−0.00000, 1.000
PUT	0.030 (0.007, 0.094)	0.056 (0.016, 0.103)	0.074 (0.042, 0.092)	−0.00394, 1.000	−0.00648, .893	−0.00254, .885
NACC	0.042 (0.003, 0.127)	0.000 (0.000, 0.007)	0.013 (0.003, 0.061)	0.00610, .707	0.00418, .893	−0.00192, .300
PALL	0.006 (0.001, 0.055)	0.004 (0.000, 0.028)	0.000 (0.000, 0.000)	0.00027, 1.000	0.00084, .503	**0.00057, .025**
THAL	0.051 (0.001, 0.106)	0.041 (0.006, 0.115)	0.019 (0.002, 0.115)	0.00167, 1.000	0.00488, .893	0.00322, .885

Means were calculated across a range of density thresholds (K = 0.10–0.25, ΔK = 0.01) and are reported with 95% bootstrap confidence intervals (CIs; 5000 resamples). Group differences in area under the curve (ΔAUC) were assessed using permutation testing (10 000 permutations). Bold values indicate statistically significant group differences after Benjamini–Hochberg correction for multiple comparisons ($p_{BH} < .05$). Abbreviations: SZ+A, schizophrenia with aggression; SZ−A, schizophrenia without aggression; HC, healthy controls; FPOL, frontal pole; POP, pars opercularis; PTRI, pars triangularis; PORB, pars orbitalis; MOFC, medial orbitofrontal cortex; LOFC, lateral orbitofrontal cortex; CMFG, caudal middle frontal gyrus; RMFG, rostral middle frontal gyrus; SFG, superior frontal gyrus; PARL, paracentral lobule; PREG, precentral gyrus; CACC, caudal anterior cingulate cortex; RACC, rostral anterior cingulate cortex; PCC, posterior cingulate cortex; ISTH, isthmus of the cingulate cortex; INS, insular cortex; TPOL, temporal pole; ENTH, entorhinal cortex; PHG, parahippocampal gyrus; TVTG, transverse temporal gyrus; ITG, inferior temporal gyrus; MTG, middle temporal gyrus; STG, superior temporal gyrus; BANKS, banks of the superior temporal sulcus; FUSI, fusiform gyrus; POSG, postcentral gyrus; PREC, precuneus; SMG, supramarginal gyrus; IPL, inferior parietal lobule; SPL, superior parietal lobule; LING, lingual gyrus; CUN, cuneus; PERI, pericalcarine cortex; LOC, lateral occipital cortex; HIPPO, hippocampus; AMYG, amygdala; CAUD, caudate nucleus; PUT, putamen; NACC, nucleus accumbens; PALL, globus pallidus; THAL, thalamus.

Table A5.18. Group differences in harmonic centrality using regional volumes residualized for CPZ-equivalent dose.

	M (95% CI)			ΔAUC, p_{BH}		
Node	SZ+A (*N* = 212)	SZ-A (*N* = 1533)	HC (*N* = 3058)	SZ+A vs SZ-A	SZ+A vs HC	SZ-A vs HC
FPOL	0.309 (0.000, 0.492)	0.026 (0.000, 0.304)	0.204 (0.000, 0.377)	0.045, .749	0.017, .920	−0.028, .589
POP	0.369 (0.261, 0.527)	0.376 (0.301, 0.471)	0.412 (0.359, 0.472)	−0.001, .961	−0.007, .920	−0.006, .671
PTRI	0.461 (0.301, 0.527)	0.438 (0.381, 0.480)	0.441 (0.393, 0.469)	0.004, .908	0.003, .920	−0.001, .921
PORB	0.469 (0.393, 0.536)	0.428 (0.396, 0.467)	0.445 (0.423, 0.496)	0.007, .759	0.004, .920	−0.003, .624
MOFC	0.597 (0.513, 0.671)	0.551 (0.511, 0.608)	0.520 (0.480, 0.562)	0.007, .759	0.012, .730	0.004, .624
LOFC	0.560 (0.501, 0.631)	0.625 (0.572, 0.682)	0.655 (0.590, 0.696)	−0.010, .749	−0.015, .730	−0.005, .624
CMFG	0.482 (0.410, 0.562)	0.467 (0.442, 0.514)	0.495 (0.479, 0.538)	0.002, .908	−0.002, .932	−0.004, .558
RMFG	0.590 (0.501, 0.650)	0.629 (0.569, 0.687)	0.602 (0.539, 0.654)	−0.006, .875	−0.002, .932	0.004, .718
SFG	0.620 (0.557, 0.671)	0.637 (0.570, 0.714)	0.648 (0.592, 0.711)	−0.002, .908	−0.004, .920	−0.002, .892
PARL	0.469 (0.389, 0.550)	0.490 (0.453, 0.532)	0.508 (0.465, 0.547)	−0.003, .908	−0.006, .920	−0.003, .720
PREG	0.489 (0.432, 0.596)	0.506 (0.459, 0.585)	0.527 (0.492, 0.580)	−0.003, .946	−0.006, .920	−0.003, .717
CACC	0.444 (0.398, 0.546)	0.379 (0.350, 0.426)	0.406 (0.370, 0.440)	0.010, .749	0.005, .920	−0.004, .589
RACC	0.578 (0.504, 0.620)	0.530 (0.493, 0.577)	0.508 (0.475, 0.565)	0.007, .749	0.011, .730	0.003, .708
PCC	0.536 (0.395, 0.613)	0.552 (0.509, 0.606)	0.553 (0.505, 0.622)	−0.002, .908	−0.002, .932	−0.000, .959
ISTH	0.357 (0.180, 0.565)	0.406 (0.360, 0.480)	0.448 (0.425, 0.498)	−0.007, .908	−0.013, .730	−0.006, .526
INS	0.676 (0.555, 0.717)	0.543 (0.494, 0.626)	0.505 (0.482, 0.560)	0.020, .703	0.026, .389	0.006, .589
TPOL	0.302 (0.000, 0.478)	0.047 (0.000, 0.189)	0.245 (0.226, 0.352)	0.040, .749	0.009, .920	**−0.030, .025**
ENTH	0.311 (0.020, 0.451)	0.063 (0.000, 0.288)	0.320 (0.300, 0.377)	0.039, .749	−0.001, .959	**−0.040, .018**
PHG	0.442 (0.289, 0.506)	0.371 (0.310, 0.428)	0.434 (0.405, 0.481)	0.011, .749	0.002, .932	−0.010, .089
TVTG	0.493 (0.458, 0.570)	0.432 (0.395, 0.509)	0.517 (0.470, 0.547)	0.009, .759	−0.004, .920	−0.013, .089
ITG	0.569 (0.486, 0.649)	0.435 (0.392, 0.505)	0.529 (0.471, 0.589)	0.021, .703	0.006, .920	−0.015, .112
MTG	0.527 (0.462, 0.612)	0.525 (0.481, 0.587)	0.493 (0.457, 0.541)	0.000, .961	0.005, .920	0.005, .624
STG	0.626 (0.551, 0.703)	0.553 (0.513, 0.610)	0.538 (0.506, 0.585)	0.011, .749	0.013, .730	0.002, .720
BANKS	0.435 (0.370, 0.503)	0.372 (0.365, 0.419)	0.408 (0.390, 0.448)	0.010, .749	0.004, .920	−0.005, .343
FUSI	0.614 (0.498, 0.685)	0.574 (0.513, 0.646)	0.574 (0.516, 0.643)	0.006, .875	0.006, .920	−0.000, .959

POSG	0.588 (0.501, 0.660)	0.484 (0.436, 0.563)	0.520 (0.478, 0.567)	0.016, .749	0.011, .730	−0.006, .608
PREC	0.586 (0.478, 0.651)	0.612 (0.530, 0.677)	0.676 (0.632, 0.716)	−0.004, .908	−0.014, .730	−0.010, .308
SMG	0.501 (0.447, 0.578)	0.428 (0.342, 0.496)	0.520 (0.484, 0.557)	0.011, .749	−0.003, .920	**−0.014, .030**
IPL	0.484 (0.410, 0.574)	0.502 (0.459, 0.570)	0.538 (0.507, 0.585)	−0.003, .908	−0.008, .811	−0.006, .558
SPL	0.493 (0.406, 0.583)	0.440 (0.389, 0.523)	0.490 (0.447, 0.545)	0.008, .875	0.001, .959	−0.007, .589
LING	0.297 (0.218, 0.496)	0.333 (0.308, 0.377)	0.401 (0.372, 0.467)	−0.005, .908	−0.015, .730	−0.010, .070
CUN	0.316 (0.227, 0.518)	0.328 (0.309, 0.394)	0.446 (0.391, 0.480)	−0.001, .961	−0.019, .728	**−0.018, .025**
PERI	0.283 (0.198, 0.439)	0.254 (0.243, 0.321)	0.362 (0.338, 0.392)	0.005, .908	−0.012, .730	**−0.016, .012**
LOC	0.386 (0.234, 0.573)	0.452 (0.410, 0.520)	0.478 (0.417, 0.541)	−0.010, .759	−0.014, .730	−0.004, .624
HIPPO	0.405 (0.324, 0.526)	0.303 (0.190, 0.479)	0.377 (0.335, 0.453)	0.015, .875	0.004, .932	−0.011, .520
AMYG	0.483 (0.352, 0.581)	0.299 (0.201, 0.480)	0.366 (0.310, 0.468)	0.028, .749	0.018, .811	−0.010, .624
CAUD	0.359 (0.234, 0.425)	0.222 (0.160, 0.327)	0.278 (0.254, 0.347)	0.021, .749	0.012, .920	−0.009, .473
PUT	0.476 (0.358, 0.538)	0.279 (0.196, 0.406)	0.346 (0.309, 0.406)	0.030, .703	0.020, .730	−0.010, .456
NACC	0.482 (0.344, 0.571)	0.203 (0.142, 0.316)	0.323 (0.286, 0.400)	0.042, .652	0.024, .730	−0.018, .089
PALL	0.403 (0.306, 0.515)	0.225 (0.159, 0.327)	0.258 (0.230, 0.291)	0.027, .703	0.022, .730	−0.005, .589
THAL	0.479 (0.320, 0.556)	0.274 (0.189, 0.442)	0.328 (0.290, 0.439)	0.031, .749	0.023, .730	−0.008, .608

Means were calculated across a range of density thresholds (K = 0.10–0.25, ΔK = 0.01) and are reported with 95% bootstrap confidence intervals (CIs; 5000 resamples). Group differences in area under the curve (ΔAUC) were assessed using permutation testing (10 000 permutations). Bold values indicate statistically significant group differences after Benjamini–Hochberg correction for multiple comparisons ($p_{BH} < .05$). Abbreviations: SZ+A, schizophrenia with aggression; SZ−A, schizophrenia without aggression; HC, healthy controls; FPOL, frontal pole; POP, pars opercularis; PTRI, pars triangularis; PORB, pars orbitalis; MOFC, medial orbitofrontal cortex; LOFC, lateral orbitofrontal cortex; CMFG, caudal middle frontal gyrus; RMFG, rostral middle frontal gyrus; SFG, superior frontal gyrus; PARL, paracentral lobule; PREG, precentral gyrus; CACC, caudal anterior cingulate cortex; RACC, rostral anterior cingulate cortex; PCC, posterior cingulate cortex; ISTH, isthmus of the cingulate cortex; INS, insular cortex; TPOL, temporal pole; ENTH, entorhinal cortex; PHG, parahippocampal gyrus; TVTG, transverse temporal gyrus; ITG, inferior temporal gyrus; MTG, middle temporal gyrus; STG, superior temporal gyrus; BANKS, banks of the superior temporal sulcus; FUSI, fusiform gyrus; POSG, postcentral gyrus; PREC, precuneus; SMG, supramarginal gyrus; IPL, inferior parietal lobule; SPL, superior parietal lobule; LING, lingual gyrus; CUN, cuneus; PERI, pericalcarine cortex; LOC, lateral occipital cortex; HIPPO, hippocampus; AMYG, amygdala; CAUD, caudate nucleus; PUT, putamen; NACC, nucleus accumbens; PALL, globus pallidus; THAL, thalamus.

Table A5.19. Group differences in within-module degree using regional volumes residualized for CPZ-equivalent dose.

	M (95% CI)			ΔAUC, p_{BH}		
Node	SZ+A (*N* = 212)	SZ-A (*N* = 1533)	HC (*N* = 3058)	SZ+A vs SZ-A	SZ+A vs HC	SZ-A vs HC
FPOL	−1.73 (−1.94, 0.00)	0.00 (−1.63, 0.00)	−1.76 (−1.87, 0.00)	−0.258, .900	0.005, .968	0.263, .219
POP	−1.51 (−1.71, 0.13)	−1.20 (−1.48, −0.11)	−1.07 (−1.46, −0.50)	−0.046, .983	−0.068, .968	−0.022, .934
PTRI	−0.85 (−1.47, 0.56)	−0.74 (−1.04, 0.51)	−0.78 (−1.24, −0.50)	−0.017, .999	−0.012, .968	0.006, .971
PORB	−0.71 (−1.50, 0.12)	−0.88 (−1.16, −0.22)	−0.87 (−1.20, −0.35)	0.026, .983	0.026, .968	−0.001, .987
MOFC	1.13 (−0.26, 2.09)	0.73 (0.05, 1.37)	0.08 (−0.50, 0.62)	0.061, .983	0.158, .753	0.097, .604
LOFC	0.35 (−0.50, 1.33)	1.81 (0.89, 2.29)	2.10 (1.13, 2.68)	−0.220, .900	−0.266, .753	−0.045, .918
CMFG	−0.53 (−1.30, 0.60)	−0.32 (−0.76, 0.19)	−0.02 (−0.36, 0.37)	−0.034, .983	−0.074, .968	−0.040, .856
RMFG	1.17 (−0.31, 1.85)	1.66 (0.78, 2.19)	1.25 (0.38, 1.90)	−0.071, .983	−0.008, .968	0.063, .918
SFG	1.71 (0.71, 2.52)	1.74 (0.97, 2.40)	2.22 (1.31, 2.85)	−0.006, .999	−0.080, .968	−0.074, .867
PARL	−0.67 (−1.51, 0.45)	0.01 (−0.50, 0.37)	−0.11 (−0.67, 0.46)	−0.102, .983	−0.081, .968	0.021, .934
PREG	−0.11 (−0.96, 1.11)	0.14 (−0.52, 1.01)	0.18 (−0.36, 0.85)	−0.037, .999	−0.043, .968	−0.006, .971
CACC	−1.00 (−1.45, 0.20)	−1.30 (−1.49, −0.96)	−1.24 (−1.53, −0.93)	0.043, .983	0.034, .968	−0.010, .934
RACC	0.40 (−0.57, 1.14)	0.49 (−0.09, 0.94)	−0.02 (−0.49, 0.62)	−0.015, .999	0.064, .968	0.080, .655
PCC	0.03 (−1.36, 1.04)	0.77 (0.12, 1.38)	0.56 (−0.13, 1.33)	−0.111, .983	−0.077, .968	0.034, .934
ISTH	−0.36 (−1.56, 0.77)	−1.28 (−1.55, −0.67)	−1.38 (−1.63, −0.52)	0.140, .983	0.160, .830	0.019, .934
INS	1.93 (0.24, 2.50)	0.51 (−0.15, 1.16)	−0.11 (−0.52, 0.48)	0.215, .900	0.312, .096	0.097, .474
TPOL	0.00 (−1.78, 0.00)	0.00 (−0.51, 0.00)	−0.58 (−0.61, 0.00)	0.000, 1.000	0.087, .968	0.087, .134
ENTH	0.00 (−1.91, 0.72)	0.00 (−1.31, 0.79)	1.15 (0.00, 1.15)	0.000, 1.000	−0.173, .968	−0.173, .134
PHG	−0.75 (−1.63, 1.04)	0.00 (−1.78, 0.00)	−0.58 (−1.04, −0.43)	−0.111, .983	−0.024, .968	0.087, .134
TVTG	−0.34 (−0.88, 0.70)	−0.86 (−1.24, −0.10)	−0.11 (−0.71, 0.27)	0.078, .983	−0.037, .968	−0.115, .219
ITG	0.29 (−0.77, 1.58)	−0.87 (−1.27, −0.19)	−0.20 (−0.77, 0.37)	0.175, .900	0.072, .968	−0.103, .367
MTG	−0.15 (−0.79, 1.03)	0.34 (−0.24, 0.93)	−0.36 (−0.71, 0.12)	−0.075, .983	0.030, .968	0.104, .341
STG	1.52 (0.31, 2.51)	0.74 (0.10, 1.25)	0.31 (−0.14, 0.88)	0.115, .983	0.182, .753	0.067, .690
BANKS	−1.09 (−1.54, 0.26)	−1.22 (−1.33, −0.94)	−1.12 (−1.33, −0.85)	0.022, .983	0.006, .968	−0.016, .918
FUSI	0.09 (−0.97, 1.94)	0.19 (−0.48, 1.02)	−0.01 (−0.77, 0.77)	−0.010, .999	0.017, .968	0.027, .934

POSG	0.96 (−0.28, 2.01)	−0.13 (−0.74, 0.77)	−0.10 (−0.62, 0.54)	0.166, .983	0.161, .753	−0.005, .971
PREC	0.52 (−0.78, 1.55)	1.37 (0.18, 1.97)	1.78 (1.11, 2.51)	−0.129, .983	−0.191, .753	−0.062, .867
SMG	−0.16 (−0.89, 0.85)	−0.83 (−1.26, −0.10)	−0.01 (−0.51, 0.48)	0.102, .983	−0.025, .968	−0.127, .148
IPL	−0.57 (−1.13, 0.87)	−0.07 (−0.61, 0.63)	0.14 (−0.34, 0.68)	−0.078, .983	−0.110, .911	−0.032, .918
SPL	−0.69 (−1.34, 0.59)	−0.81 (−1.30, 0.05)	−0.73 (−1.30, −0.01)	0.013, .999	0.005, .968	−0.008, .971
LING	0.53 (−0.87, 1.25)	0.87 (0.00, 1.19)	0.92 (−0.74, 1.10)	−0.050, .983	−0.061, .968	−0.012, .934
CUN	0.59 (−0.70, 1.26)	0.87 (0.00, 0.93)	−0.20 (−0.63, 0.38)	−0.040, .983	0.123, .753	**0.163, .020**
PERI	−0.03 (−1.15, 0.79)	−0.87 (−0.87, 0.00)	0.40 (−1.07, 0.65)	0.125, .983	−0.068, .968	**−0.194, .023**
LOC	−0.73 (−1.55, 0.58)	−0.87 (−0.93, 0.16)	0.26 (−0.40, 0.62)	0.017, .996	−0.153, .753	−0.170, .075
HIPPO	−0.41 (−1.21, 1.05)	−0.79 (−1.14, 1.11)	−0.31 (−0.80, 0.37)	0.057, .983	−0.019, .968	−0.077, .474
AMYG	0.89 (−0.79, 1.41)	−0.16 (−0.57, 0.62)	−0.18 (−0.45, 0.46)	0.158, .900	0.161, .753	0.003, .971
CAUD	−0.94 (−1.67, 0.54)	0.00 (−0.37, 0.87)	−0.13 (−0.48, 0.36)	−0.143, .900	−0.119, .753	0.023, .918
PUT	1.55 (−0.50, 1.92)	1.74 (0.87, 1.92)	1.59 (1.27, 1.84)	−0.025, .999	−0.006, .968	0.020, .918
NACC	−0.50 (−1.40, 1.08)	−1.18 (−1.38, −0.49)	0.46 (−0.05, 0.81)	0.101, .983	−0.144, .753	**−0.245, .002**
PALL	0.32 (−0.96, 1.45)	−0.23 (−0.87, 0.24)	−1.45 (−1.73, −1.03)	0.080, .983	0.269, .096	**0.189, .002**
THAL	−0.16 (−1.32, 0.98)	0.62 (−0.57, 1.06)	0.02 (−0.82, 0.45)	−0.117, .983	−0.031, .968	0.086, .803

Means were calculated across a range of density thresholds (K = 0.10–0.25, ΔK = 0.01) and are reported with 95% bootstrap confidence intervals (CIs; 5000 resamples). Group differences in area under the curve (ΔAUC) were assessed using permutation testing (10 000 permutations). Bold values indicate statistically significant group differences after Benjamini–Hochberg correction for multiple comparisons ($p_{BH} < .05$). Abbreviations: SZ+A, schizophrenia with aggression; SZ−A, schizophrenia without aggression; HC, healthy controls; FPOL, frontal pole; POP, pars opercularis; PTRI, pars triangularis; PORB, pars orbitalis; MOFC, medial orbitofrontal cortex; LOFC, lateral orbitofrontal cortex; CMFG, caudal middle frontal gyrus; RMFG, rostral middle frontal gyrus; SFG, superior frontal gyrus; PARL, paracentral lobule; PREG, precentral gyrus; CACC, caudal anterior cingulate cortex; RACC, rostral anterior cingulate cortex; PCC, posterior cingulate cortex; ISTH, isthmus of the cingulate cortex; INS, insular cortex; TPOL, temporal pole; ENTH, entorhinal cortex; PHG, parahippocampal gyrus; TVTG, transverse temporal gyrus; ITG, inferior temporal gyrus; MTG, middle temporal gyrus; STG, superior temporal gyrus; BANKS, banks of the superior temporal sulcus; FUSI, fusiform gyrus; POSG, postcentral gyrus; PREC, precuneus; SMG, supramarginal gyrus; IPL, inferior parietal lobule; SPL, superior parietal lobule; LING, lingual gyrus; CUN, cuneus; PERI, pericalcarine cortex; LOC, lateral occipital cortex; HIPPO, hippocampus; AMYG, amygdala; CAUD, caudate nucleus; PUT, putamen; NACC, nucleus accumbens; PALL, globus pallidus; THAL, thalamus.

Table A5.20. Group differences in participation coefficient using regional volumes residualized for CPZ-equivalent dose.

	M (95% CI)			ΔAUC, p_{BH}		
Node	SZ+A (*N* = 212)	SZ-A (*N* = 1533)	HC (*N* = 3058)	SZ+A vs SZ-A	SZ+A vs HC	SZ-A vs HC
FPOL	0.00 (0.00, 0.18)	0.00 (0.00, 0.00)	0.00 (0.00, 0.00)	0.000, 1.000	0.000, 1.000	0.000, 1.000
POP	0.00 (0.00, 0.35)	0.00 (0.00, 0.18)	0.00 (0.00, 0.00)	0.000, 1.000	0.000, 1.000	0.000, 1.000
PTRI	0.00 (0.00, 0.46)	0.00 (0.00, 0.41)	0.00 (0.00, 0.00)	0.000, 1.000	0.000, 1.000	0.000, 1.000
PORB	0.00 (0.00, 0.37)	0.00 (0.00, 0.47)	0.02 (0.00, 0.16)	0.000, 1.000	−0.001, .899	−0.001, .921
MOFC	0.00 (0.00, 0.41)	0.01 (0.00, 0.09)	0.01 (0.00, 0.11)	−0.000, .998	−0.001, 1.000	−0.000, 1.000
LOFC	0.02 (0.00, 0.41)	0.00 (0.00, 0.20)	0.06 (0.00, 0.11)	0.003, 1.000	−0.006, 1.000	−0.009, 1.000
CMFG	0.04 (0.00, 0.28)	0.00 (0.00, 0.00)	0.00 (0.00, 0.07)	0.005, .538	0.005, .886	0.000, 1.000
RMFG	0.00 (0.00, 0.37)	0.06 (0.00, 0.22)	0.09 (0.00, 0.18)	−0.010, .867	−0.013, .899	−0.003, 1.000
SFG	0.04 (0.00, 0.21)	0.08 (0.00, 0.20)	0.00 (0.00, 0.10)	−0.006, 1.000	0.005, .935	0.011, .921
PARL	0.00 (0.00, 0.21)	0.00 (0.00, 0.00)	0.00 (0.00, 0.00)	0.000, 1.000	0.000, 1.000	0.000, 1.000
PREG	0.00 (0.00, 0.24)	0.00 (0.00, 0.06)	0.00 (0.00, 0.08)	0.000, 1.000	0.000, 1.000	0.000, 1.000
CACC	0.08 (0.00, 0.44)	0.00 (0.00, 0.00)	0.00 (0.00, 0.00)	0.011, .403	0.011, .886	0.000, 1.000
RACC	0.18 (0.00, 0.49)	0.00 (0.00, 0.02)	0.02 (0.00, 0.12)	0.026, .403	0.024, .886	−0.002, .921
PCC	0.01 (0.00, 0.45)	0.00 (0.00, 0.09)	0.00 (0.00, 0.10)	0.001, .867	0.001, 1.000	0.000, 1.000
ISTH	0.33 (0.00, 0.56)	0.12 (0.00, 0.40)	0.49 (0.00, 0.50)	0.032, .867	−0.023, 1.000	−0.055, .201
INS	0.18 (0.00, 0.41)	0.02 (0.00, 0.22)	0.00 (0.00, 0.09)	0.023, .844	0.026, .887	0.002, 1.000
TPOL	0.38 (0.00, 0.59)	0.00 (0.00, 0.12)	0.00 (0.00, 0.38)	0.057, .448	0.057, .886	0.000, 1.000
ENTH	0.41 (0.00, 0.57)	0.09 (0.00, 0.26)	0.00 (0.00, 0.50)	0.049, .448	0.062, .886	0.012, .921
PHG	0.47 (0.00, 0.60)	0.34 (0.00, 0.59)	0.66 (0.60, 0.72)	0.018, 1.000	−0.028, .936	**−0.047, .012**
TVTG	0.00 (0.00, 0.26)	0.00 (0.00, 0.08)	0.00 (0.00, 0.02)	0.000, 1.000	0.000, 1.000	0.000, 1.000
ITG	0.20 (0.00, 0.62)	0.00 (0.00, 0.07)	0.05 (0.00, 0.24)	0.030, .772	0.023, .899	−0.007, 1.000
MTG	0.12 (0.00, 0.59)	0.00 (0.00, 0.09)	0.00 (0.00, 0.10)	0.018, .772	0.018, .899	0.000, 1.000
STG	0.08 (0.00, 0.37)	0.00 (0.00, 0.10)	0.00 (0.00, 0.08)	0.012, .863	0.012, .899	0.000, 1.000
BANKS	0.00 (0.00, 0.46)	0.00 (0.00, 0.00)	0.00 (0.00, 0.00)	0.000, 1.000	0.000, 1.000	0.000, 1.000
FUSI	0.49 (0.00, 0.68)	0.35 (0.14, 0.47)	0.33 (0.22, 0.45)	0.022, .998	0.024, .899	0.001, 1.000

POSG	0.10 (0.00, 0.40)	0.00 (0.00, 0.02)	0.00 (0.00, 0.11)	0.014, .772	0.014, .887	0.000, 1.000
PREC	0.13 (0.00, 0.49)	0.08 (0.00, 0.22)	0.26 (0.00, 0.33)	0.007, 1.000	−0.020, 1.000	−0.027, .367
SMG	0.00 (0.00, 0.24)	0.00 (0.00, 0.00)	0.00 (0.00, 0.05)	0.000, 1.000	0.000, 1.000	0.000, 1.000
IPL	0.00 (0.00, 0.53)	0.00 (0.00, 0.10)	0.00 (0.00, 0.14)	0.000, 1.000	0.000, 1.000	0.000, 1.000
SPL	0.17 (0.00, 0.44)	0.00 (0.00, 0.19)	0.32 (0.00, 0.43)	0.027, .844	−0.022, 1.000	−0.049, .358
LING	0.00 (0.00, 0.55)	0.07 (0.00, 0.45)	0.11 (0.00, 0.52)	−0.009, 1.000	−0.015, 1.000	−0.006, 1.000
CUN	0.10 (0.00, 0.49)	0.00 (0.00, 0.45)	0.46 (0.00, 0.48)	0.014, 1.000	−0.056, .899	**−0.070, .014**
PERI	0.00 (0.00, 0.35)	0.00 (0.00, 0.00)	0.00 (0.00, 0.19)	0.000, 1.000	0.000, 1.000	0.000, 1.000
LOC	0.33 (0.00, 0.58)	0.44 (0.40, 0.52)	0.43 (0.00, 0.51)	−0.015, .867	−0.016, 1.000	−0.001, 1.000
HIPPO	0.05 (0.00, 0.51)	0.31 (0.04, 0.55)	0.35 (0.03, 0.48)	−0.040, .867	−0.046, .886	−0.006, 1.000
AMYG	0.27 (0.00, 0.59)	0.18 (0.02, 0.56)	0.18 (0.00, 0.42)	0.016, 1.000	0.016, 1.000	−0.000, 1.000
CAUD	0.00 (0.00, 0.38)	0.00 (0.00, 0.12)	0.00 (0.00, 0.00)	0.000, 1.000	0.000, 1.000	0.000, 1.000
PUT	0.27 (0.00, 0.54)	0.00 (0.00, 0.42)	0.00 (0.00, 0.11)	0.041, .867	0.041, .899	0.000, 1.000
NACC	0.48 (0.00, 0.61)	0.00 (0.00, 0.08)	0.00 (0.00, 0.15)	0.071, .403	0.071, .886	0.000, 1.000
PALL	0.03 (0.00, 0.48)	0.00 (0.00, 0.33)	0.00 (0.00, 0.00)	0.004, .867	0.004, .886	0.000, 1.000
THAL	0.35 (0.00, 0.57)	0.02 (0.00, 0.47)	0.00 (0.00, 0.26)	0.051, .867	0.052, .899	0.001, 1.000

Means were calculated across a range of density thresholds (K = 0.10–0.25, ΔK = 0.01) and are reported with 95% bootstrap confidence intervals (CIs; 5000 resamples). Group differences in area under the curve (ΔAUC) were assessed using permutation testing (10 000 permutations). Bold values indicate statistically significant group differences after Benjamini–Hochberg correction for multiple comparisons (p_{BH} < .05). Abbreviations: SZ+A, schizophrenia with aggression; SZ−A, schizophrenia without aggression; HC, healthy controls; FPOL, frontal pole; POP, pars opercularis; PTRI, pars triangularis; PORB, pars orbitalis; MOFC, medial orbitofrontal cortex; LOFC, lateral orbitofrontal cortex; CMFG, caudal middle frontal gyrus; RMFG, rostral middle frontal gyrus; SFG, superior frontal gyrus; PARL, paracentral lobule; PREG, precentral gyrus; CACC, caudal anterior cingulate cortex; RACC, rostral anterior cingulate cortex; PCC, posterior cingulate cortex; ISTH, isthmus of the cingulate cortex; INS, insular cortex; TPOL, temporal pole; ENTH, entorhinal cortex; PHG, parahippocampal gyrus; TVTG, transverse temporal gyrus; ITG, inferior temporal gyrus; MTG, middle temporal gyrus; STG, superior temporal gyrus; BANKS, banks of the superior temporal sulcus; FUSI, fusiform gyrus; POSG, postcentral gyrus; PREC, precuneus; SMG, supramarginal gyrus; IPL, inferior parietal lobule; SPL, superior parietal lobule; LING, lingual gyrus; CUN, cuneus; PERI, pericalcarine cortex; LOC, lateral occipital cortex; HIPPO, hippocampus; AMYG, amygdala; CAUD, caudate nucleus; PUT, putamen; NACC, nucleus accumbens; PALL, globus pallidus; THAL, thalamus.

Table A5.21. Group differences in canonical brain network density using regional volumes residualized for CPZ-equivalent dose.

	M (95% CI)			ΔAUC, p_{BH}		
Metric	SZ+A (*N* = 212)	SZ-A (*N* = 1533)	HC (*N* = 3058)	SZ+A vs SZ-A	SZ+A vs HC	SZ-A vs HC
Salience network						
Network-rest	0.28 (0.19, 0.33)	0.19 (0.15, 0.24)	0.14 (0.12, 0.18)	0.014, .292	**0.021, .027**	0.007, .172
Central executive network						
Within-network	0.34 (0.05, 0.55)	0.55 (0.35, 0.66)	0.29 (0.17, 0.51)	−0.032, .960	0.009, 1.000	0.041, .377
Network-rest	0.18 (0.14, 0.24)	0.23 (0.19, 0.26)	0.24 (0.21, 0.26)	−0.007, .403	−0.009, .358	−0.002, .608
Default mode network						
Within-network	0.14 (0.10, 0.21)	0.17 (0.14, 0.20)	0.18 (0.15, 0.20)	−0.004, .960	−0.006, 1.000	−0.002, .872
Network-rest	0.15 (0.13, 0.17)	0.15 (0.14, 0.17)	0.15 (0.14, 0.16)	0.000, .882	0.001, .970	0.001, .608
Language network						
Within-network	0.34 (0.24, 0.52)	0.35 (0.30, 0.40)	0.31 (0.30, 0.38)	−0.002, 1.000	0.004, 1.000	0.006, .872
Network-rest	0.16 (0.13, 0.21)	0.19 (0.16, 0.22)	0.16 (0.14, 0.18)	−0.005, .512	−0.000, .970	0.005, .172
Reward network						
Within-network	0.39 (0.26, 0.48)	0.32 (0.25, 0.38)	0.39 (0.33, 0.43)	0.011, .960	0.000, 1.000	−0.010, .377
Network-rest	0.15 (0.12, 0.17)	0.16 (0.14, 0.18)	0.14 (0.12, 0.15)	−0.003, .528	0.001, .970	0.004, .172
Affective control network						
Within-network	0.32 (0.25, 0.38)	0.33 (0.28, 0.37)	0.32 (0.28, 0.34)	−0.001, 1.000	0.000, 1.000	0.002, .872
Network-rest	0.14 (0.13, 0.17)	0.17 (0.15, 0.17)	0.14 (0.14, 0.15)	−0.003, .270	0.000, .970	**0.003, .026**

Means were calculated across a range of density thresholds (K = 0.10–0.25, ΔK = 0.01) and are reported with 95% bootstrap confidence intervals (CIs; 5000 resamples). Group differences in area under the curve (ΔAUC) were assessed using permutation testing (10 000 permutations). Bold values indicate statistically significant group differences after Benjamini–Hochberg correction for multiple comparisons (p_{BH} < .05). Abbreviations: SZ+A, schizophrenia with aggression; SZ−A, schizophrenia without aggression; HC, healthy controls.

Appendix 6
References

93. Menon V. 20 years of the default mode network: a review and synthesis. Neuron. 2023;111(16):2469-87.
94. Hickok G, Poeppel D. The cortical organization of speech processing. Nat Rev Neurosci. 2007;8(5):393-402.
95. Bartra O, McGuire JT, Kable JW. The valuation system: a coordinate-based meta-analysis of BOLD fMRI experiments examining neural correlates of subjective value. Neuroimage. 2013;76:412-27.
96. Kesby JP, Murray GK, Knolle F. Neural circuitry of salience and reward processing in psychosis. Biol Psychiatry Glob Open Sci. 2023;3(1):33-46.
